# Quantitative Proteomic Profiling of Early and Late Responses to Salicylic Acid in Cucumber Leaves

**DOI:** 10.1371/journal.pone.0161395

**Published:** 2016-08-23

**Authors:** Chun-Juan Dong, Ning Cao, Liang Li, Qing-Mao Shang

**Affiliations:** Institute of Vegetables and Flowers, Chinese Academy of Agricultural Sciences,Key Laboratory of Horticultural Crop Biology and Germplasm Innovation, Ministry of Agriculture, Beijing, 100081, P.R.China; Institute of Botany Chinese Academy of Sciences, CHINA

## Abstract

Salicylic acid (SA) is an important phytohormone that plays vital regulatory roles in plant growth, development, and stress responses. However, studies on the molecular mechanism of SA, especially during the early SA responses, are lagging behind. In this study, we initiated a comprehensive isobaric tag for relative and absolute quantitation (iTRAQ)-based proteomic analysis to explore the early and late SA-responsive proteins in leaves of cucumber (*Cucumis sativus* L.) seedlings. Upon SA application through the roots, endogenous SA accumulated in cucumber leaves. By assaying the changes in marker gene expression and photosynthetic rate, we collected samples at 12 h and 72 h post treatment (hpt) to profile the early and late SA responsiveness, respectively. The iTRAQ assay followed by tandem mass spectrometry revealed 135 differentially expressed proteins (DEPs) at 12 hpt and 301 DEPs at 72 hpt. The functional categories for these SA-responsive proteins included in a variety of biochemical processes, including photosynthesis, redox homeostasis, carbohydrate and energy metabolism, lipid metabolism, transport, protein folding and modification, proteolysis, cell wall organization, and the secondary phenylpropanoid pathway. Conclusively, based on the abundant changes of these DEPs, together with their putative functions, we proposed a possible SA-responsive protein network. It appears that SA could elicit reactive oxygen species (ROS) production via enhancing the photosynthetic electron transferring, and then confer some growth-promoting and stress-priming effects on cells during the late phase, including enhanced photosynthesis and ROS scavenging, altered carbon metabolic flux for the biosynthesis of amino acids and nucleotides, and cell wall reorganization. Overall, the present iTRAQ assay provides higher proteome coverage and deepened our understanding of the molecular basis of SA-responses.

## Introduction

Salicylic acid (SA) is a small phenolic phytohormone that plays vital roles in various plant developmental processes, as well as in resistance to multiple biotic and abiotic stresses [1–3]. In the context of biotic stress, SA acts as a crucial signalling molecule to confer onto plants both local and systemic immunity responses [3]. Pathogen-induced SA is mainly synthesized via the isochorismate synthase (ICS) and phenylalanine ammonia-lyase (PAL) pathways, which are localized in chloroplasts and cytosol, respectively [4]. Then, the produced SA triggers extensive transcriptional reprogramming in which Non-expressor of pathogenesis-related 1 (NPR1) functions as a central coactivator of TGA transcription factors [1, 4]. Recently, two alternative SA receptors, NPR3 and NPR4, have also been proposed. NPR3 and NPR4 perceive SA, thereby regulating the accumulation of NPR1 protein [5, 6]. The accumulated NPR1 can trigger the expression of a series of *Pathogenesis-Related* (*PR*) genes and promote SA-mediated resistance [4]. Moreover, exogenously sourced SA can also induce plant resistance to pathogens [7, 8].

In addition to pathogen resistance, SA also functions as a regulatory signal mediating several physiological processes, such as seed production and germination, vegetative growth, thermogenesis, flower formation, and senescence [2]. In addition, recent physiological studies have established that SA can contribute to the regulation of stomatal closure, photosynthesis, proline metabolism, and antioxidant system activation, thereby improving plant tolerance to multiple abiotic stresses, including salinity, drought, cold, heat, heavy metals, and ozone [9, 10]. However, the global biochemical and molecular mechanisms that potentially underpin SA functions are rarely discussed.

In recent years, high-throughput transcriptomic or proteomic methods have provided powerful tools to analyze the integral changes in SA responsiveness. Salzman *et al*. performed a microarray study of SA-induced transcriptional changes in sorghum seedlings, wherein many SA-responsive genes, including numerous *PR* genes, and enzymes that are involved in the phenylpropanoid pathway, redox regulation, and cell wall fortification were identified [11]. Additionally, proteomics-based techniques have been successfully applied to analyze the SA-induced changes in the proteome patterns. By traditional two-dimensional electrophoresis (2-DE), the mechanisms of SA-induced pathogen resistance in rice [12], peach [13] and *Vigna Mungo* [14], and SA-enhanced tolerance to drought and salinity stress in wheat seedlings [15, 16], grape berries [17] and Arabidopsis seeds [18] has been explored. An increasing number of diverse proteins have been found to bind SA. These proteins mainly function in photosynthesis, carbohydrate metabolism, redox signalling, and ion homeostasis. However, not all types of proteins are amenable to gel-based proteomic techniques [19]. To overcome the limitations of 2-DE and to improve the throughput of proteomic studies [20], isobaric tag for relative and absolute quantitation (iTRAQ) has been successfully used. Recently, an SA-responsive iTRAQ assay was performed to detect the extracellular matrix proteomic changes in Arabidopsis suspension cells that had been treated with 200 μM SA, a cell-death-causing dose [21]. This proteomic assay revealed a staggering 55.3% of extracellular matrix proteins to be responsive to SA and indicated the powerful utility of iTRAQ labelling for SA responsiveness in plants.

Cucumber (*Cucumis sativus* L.) is an economically important crop and a model system for studies of sex determination and vascular transport [22]. Young cucumber seedlings are susceptible to pathogen infections, cold, drought, and salinity stresses due to their low biomass, undeveloped protective structure, and large water and nutrient demands for growth and expansion. Exogenous SA can not only enhance seedling growth but also function as an “effective therapeutic agent” to protect cucumber against subsequent environmental stresses [23–25]. In our previous report, a 2-DE assay was carries out to reveal the SA-induced proteomic changes in cotyledons, and 59 SA-responsive proteins were successfully identified [26]. These SA-responsive proteins mainly function in signal transduction, photosynthesis, energy metabolism, ROS scavenging and ion homeostasis. However, this assay, as well as the proteomic studies mentioned for other species, only focused on the late SA responses, which occur three or five days after SA treatment. The molecular changes during early SA responses remain obscure. Consequently, it is desirable to propose a comprehensive and dynamic protein network behind SA application.

In this study, using the high-throughput iTRAQ technique, the SA-responsive proteomic changes were successfully addressed. The iTRAQ assay offered better protein coverage and made full use of the cucumber genome-sequencing initiatives [22]. Furthermore, both the early and late SA-responsive phases were included in this study and thus complement the proteomic analysis that was performed in the previous 2-DE experiment [26]. The results indicate that SA treatment led to a dramatic proteomic response involving 381 proteins, with 135 and 301 proteins in the early and late stages, respectively. The functional annotation of these proteins combined and their abundant changes revealed distinct biochemical and physiological changes during early and late SA responses. Finally, a possible protein network supporting the SA responsiveness in cucumber leaves was drawn. Our comprehensive and deep proteomic survey not only reveals new insight into the molecular basis of SA responsiveness but also provides a framework for further detailed studies of each metabolic pathway.

## Materials and Methods

### Plant growth and treatment

Cucumber (*C*. *sativus* L. cv. Zhongnong 203) seeds were surface sterilized in 5% NaClO and sown in vermiculite. After 7 days, seedlings with cotyledons completely outspread were transferred to a hydroponic-culture system containing 1/2-strength Hoagland solution. The growth conditions were as follows: 12-h photoperiod, 28°C/18°C day/night temperature, a relative humidity of 75%-85% and a light intensity of 300 μmol m^-2^ s^-1^. The nutrient solution was replaced every 3 days. After 14 days, 50 μM SA was added to the nutrient solution. Seedlings without SA treatment were used as control. At 12 and 72 hpt, the first true leaves were harvested for the proteomic assays. The experiments were performed in three biological replicates.

### Protein extraction, in-solute digestion, and iTRAQ-labelling

Proteins were extracted using the trichloroacetic acid (TCA)-acetone method as previously reported [26]. The extracted protein pellets were dissolved in the buffer containing 8 M urea, 30 mM HEPES, 1 mM PMSF, 2 mM EDTA, and 10 mM DTT. To remove the major leaf protein Rubisco, 60% PEG-4000 (w/v) was added to a final concentration of 16%. After 30 min of stirring at 4°C, the solution was centrifuged at 12,000 *g* for 45 min. The supernatant was retained for analysis. The protein concentrations were determined with the BCA Protein Assay (Pierce, Rockford, IL).

The total protein (100 μg) was reduced by adding DTT to a final concentration of 10 mM and incubating for 1 h at room temperature. Subsequently, iodoacetamide was added to a final concentration of 55 mM, and the mixture was incubated for 1 h in the dark. Then, DTT (10 mM) was added again to the mixture to remove any free iodoacetamide. Proteins were then diluted using 50% triethylammonium bicarbonate and 1 mM CaCl_2_ to reduce the urea concentration to less than 0.6 M and digested with 4.3 μg of trypsin (1 μg μl^-1^, Promega, Madison, WI, USA) for 24 h at 37°C. The resulting peptide solution was acidified with 10% trifluoroacetic acid, desalted on a C18 solid-phase extraction cartridge (Sigma-Aldrich, St Louis, MO, USA), and quantified by spectral density with UV light at 280 nm.

Approximately 100 μg of peptides from each sample were labelled with iTRAQ reagents following the manufacturer’s instructions (Applied Biosystems). The peptides that were extracted from the control samples that were harvested at 12 h and 72 h were labelled with iTRAQ-113 and -114 reagents, respectively, and the peptides that were harvested from SA-treated samples at 12 hpt and 72 hpt were labelled with iTRAQ-115 and -116 reagents, respectively. The labelling reaction was stopped after 2 h of incubation by adding 1 ml of buffer containing 10 mM K_2_HPO_4_ and 25% acetonitrile (ACN) to each vial. Then, all of the samples were pooled and adjusted to pH 3.0 with concentrated phosphoric acid. Each of the three biological replicateswas labelled separately. The performance of iTRAQ labelling was relatively constant (> 99%) based on the MS detection as previously described [27].

### Peptide separation by strong cation exchange (SCX) and mass spectrometric analysis

The pool of labelled peptides was fractioned using a high-performance liquid chromatography (HPLC, Shimazu, Nakagyo-ku, Tokyo, Japan) system that was equipped with a PhenomenexLuna SCX column (5 μm, 4.6 × 250 mm, and 100 Å pores) at a flow rate of 0.3 ml min^-1^. First, samples were loaded onto the column after being diluted 10-fold in solvent A (10 mM K_2_HPO_4_/25% ACN, pH 3.0). Then, the column was washed with solvent A for 20 min, and the peptides were eluted using the following gradient: 0–50 min, 0% solvent B (2 M KCl in 10 mM K_2_HPO_4_/25% ACN pH 3.0); 50–51 min, 0–5% B; 51–66 min, 5–30% B; 66–71 min, 30–50% B; 71–76 min, 50% B; 76–81 min, 50–100% B; and 81–91 min, 100% B. The collected fractions were pooled into 12 final fractions. Each fraction was desalted with a Strata-X C18 column (Phenomenex) and vacuum dried.

Then, the labelled peptides were separated and identified using a nano-HPLC system (Waters Corporation, Milford, MA, USA) that was equipped with a Q-Exactive hybrid quadrupole-Orbitrap MS (Thermo Fisher Scientific Inc. Rockford, IL., USA). Peptides of each fraction (5 μl injections) were resolved in solvent A (0.1% formic acid) and loaded into the nano-HPLC system at a flow rate of 400 nl min^-1^. The peptides were eluted by the application of a linear gradient from 5% solvent B (95% ACN, 0.1% formic acid) to 30% B over 40 min, followed by increasing to 60% B in 5 min, to 80% in 3 min and holding for 7 min; the chromatographic conditions (5%) were restored in 3 min and equilibrated in solvent A for 10 min. The eluates were transferred to Q-Exactive MS, which was run in positive ion mode and in a data-dependent manner with a full MS scan from 350–6000 *m*/*z* with a resolution of 70,000, an MS/MS scan with a minimum signal threshold of 17,500, and isolation at 2 Da. An ion spray voltage of 1.8 kV was applied directly to the LC buffer distal to the chromatography column. The ion transfer tube temperature was set to 300°C. For the MS/MS acquisition mode, a higher collision energy dissociation (HCD) was employed, and a normalized collision energy (NCE) of 28% was adopted for the optimized HCD acquisition efficiency.

### Database search and quantitative data analysis

The raw MS/MS data were converted into MGF format using Proteome Discoverer 1.3 (Thermo Fisher Scientific). The exported MGF files were searched by Mascot 2.3.0 (Matrix Science, Boston, MA, USA) against the cucumber protein database (Cucumber_v2, 25,600 sequences, http://www.icugi.org/cgi-bin/ICuGI/genome/index.cgi?organism=cucumber) with a precursor mass tolerance set at 15 ppm and product ion tolerance of 20 mmu. An automatic decoy database search was performed. The carbamidomethylation of cysteines was set as a fixed modification (C), and oxidation of methionines (M), Gln to pyro-Glu (N-term Q), and 8-plex iTRAQ modifications of N-term, K, and Y were considered variable modifications. A maximum of one miscleavage was accepted.

A protein with at least one unique peptide and a false discovery rate (FDR) < 0.05 qualified for further quantification analysis. The fold change in protein abundance was defined as the median ratio of all of the significantly matched spectra with tag signals. Spectra that were assigned to more than one protein were not used for quantification. The average iTRAQ ratios and standard deviations were calculated for each protein using all of the available treatment/control iTRAQ pairs. In this study, we used *P* < 0.05 (Student *t*-test) and fold change ≥ 1.50 or ≤ 0.67 as the thresholds to judge the significance level of differential protein expression.

### SA assay by UPLC

Endogenous SA contents were analyzed using an ACQUITY ultra-performance liquid chromatography (UPLC, Waters, Milford, MA, USA) as described previously [13]. *O*-anisic acid (Sigma-Aldrich) added before the SA extraction was used as an internal standard.

### Measurement of photosynthesis

*P*_n_ was measured using a LI-COR 6400 portable gas analysis system with a light-emitting diode light source (LI-COR Inc., Lin-coln, NE, USA). At least five seedlings from each treatment were selected.

### Quantitative determination and histochemical staining of H_2_O_2_

The quantitative determination and diaminobenzidine (DAB) histochemical staining of H_2_O_2_ in cucumber leaves was performed as described previously [25].

Intact chloroplasts were isolated by a modified method as previously described [28]. The leaves (10 g FW) were homogenized in buffer A at 4°C. The homogenate was squeezed through four layers of gauze, after which the filtrate was centrifuged at 2,000×*g* for 30 s. The pellets were rinsed twice with buffer Band then subjected to discontinuous density gradient centrifugation using Percoll gradients of 10%, 40% and 80%. After centrifugation at 6,000×*g* for 20 min, the intact chloroplast layer between the 40% and 80% fractions was collected. Throughout the isolation, no H_2_O_2_ was added to the buffer. The intactness of the chloroplasts was approximately 85%, as shown by the Hill reaction [29]. The H_2_O_2_ content was assayed in intact chloroplasts that were diluted 2-fold with 80% acetone and was measured by monitoring the *A*_410_ of a titanium-peroxide complex as previously described [25]. The H_2_O_2_ content was expressed in nanomoles per milligram of chlorophyll (nmol mg^-1^ Chl).

### RNA extraction and real-time PCR analysis

Total RNA in cucumber leaves was isolated using TRIzol reagent (Invitrogen, Carlsbad, CA, USA) according to the manufacturer’s protocol. Residual DNA was digested by DNase I (Sigma-Aldrich). 2.0 μg of total RNA was used as the template for first-strand cDNA synthesis using M-MLV Reverse Transcription System (Promega). Real-time PCR was then performed using gene-specific primers (S1 Table) and Roche Power SYBR^®^ Green Real-time PCR Master Mix with a Light Cycler^®^ 96 system (Roche Diagnostics, Basel, Switzerland). The relative transcript abundance was calculated using the comparative *C*_t_ method. The house-keeping gene *Actin* was used as a standard reference. Each sample was run in triplicate.

## Results

### Physiological responses to SA in cucumber leaves

**Fig 1:**
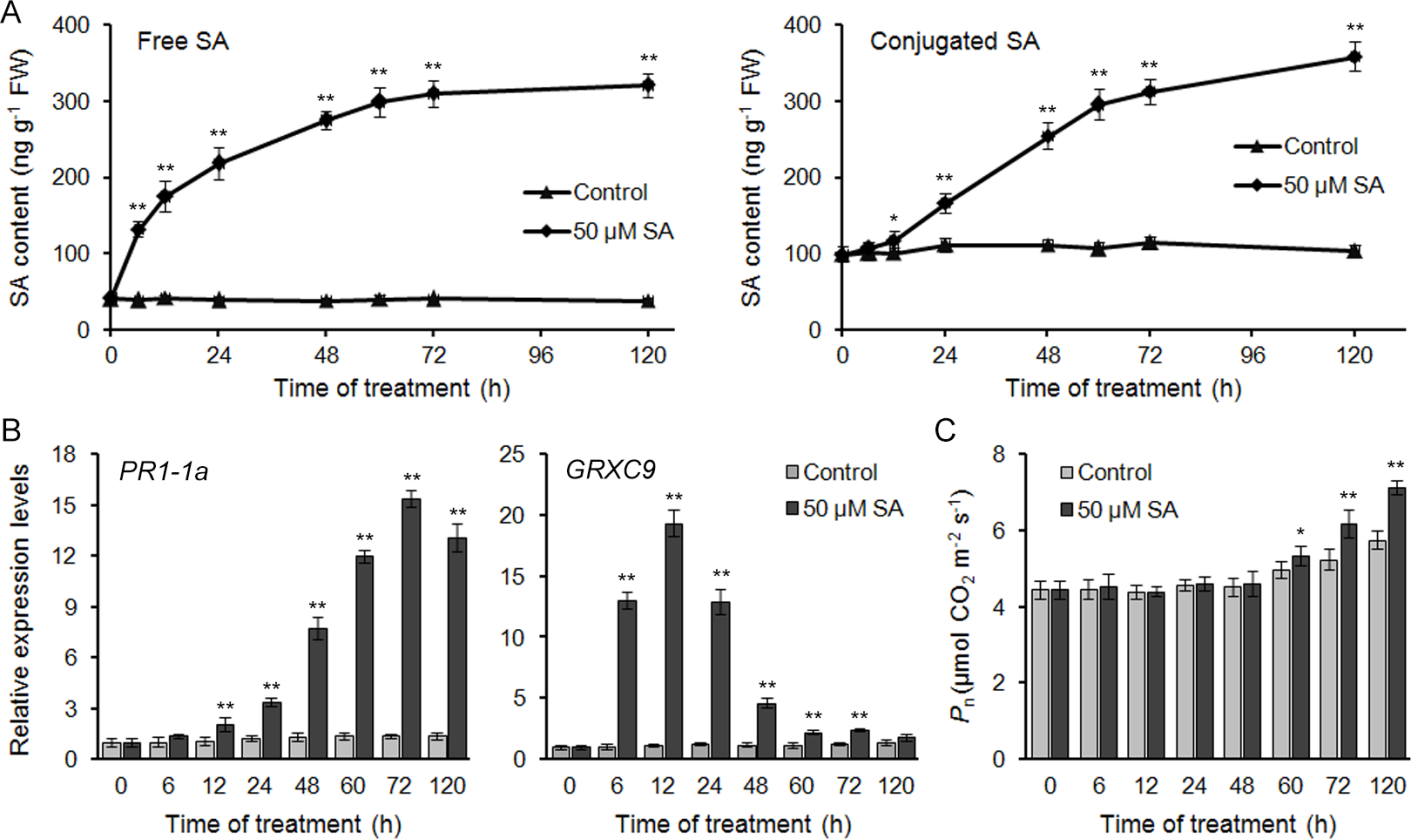
Time courses of endogenous SA accumulation, *PR1-1a*and *GRXC9* expression, and net photosynthetic rate (*P*_n_) in the leaves of cucumber seedlings upon SA treatment. (A) The contents of endogenous SA, in both free and conjugated forms. (B, C) The relative expression levels of *PR1-1a* and *GRXC9*(B) and *P*_n_ (C) at different times after SA treatment. The seedlings that were treated without SA were used as controls. Data represent the means of three replicates ± SD.*P < 0.05 and **P < 0.01 compared to the corresponding value from the control.

The application of 50 μM SA through roots indeed triggered the accumulation of endogenous SA in cucumber leaves in both free and conjugated forms (Fig 1A). Upon SA treatment, the free SA increased over time within 48 h post treatment (hpt) and then plateaued (approximately 7.5-fold compared to the control) at 72 hpt. For the conjugated SA, the accumulation occurred over different time courses, with a discernible increase until 12 h after SA treatment, followed by a rapid increase. It should be noted that in the control sample, both free and conjugated SA levels were relatively constant in seedlings over the same time period (Fig 1A), excluding the effects of development on the SA accumulation.

The *PR1-1a* gene is a classic SA marker. Consistent with the endogenous SA contents, *PR1-1a* transcript levels showed a time-dependent induction after SA treatment (Fig 1B). *PR1-1a* expression was substantially induced at 12 hpt and increased gradually to a peak at 72 hpt. Then, the expression of *PR1-1 a*s lightly decreased. *GRXC9* is an early SA-induced gene in Arabidopsis [30, 31]. In cucumber, the expression of the *GRXC9* homologue gene preceded that of *PR1-1a*. The transcript was induced rapidly within 6 h and peaked at 12 hpt (Fig 1B).

We further evaluated the photosynthetic responses of cucumber seedlings to SA treatment. As shown in Fig 1C, until 60 hpt, a slight SA-induced increase in the net photosynthetic rate (*P*_n_) was detected. At 72 hpt and 120 hpt, the *P*_n_ increased more, to approximately 1.18- and 1.24-fold compared to the control, respectively. Taken together, these results suggest that the application of 50 μM SA could indeed induce the accumulation of endogenous SA in cucumber leaves. Then, some early molecular and later physiological responses were caused at 12 hpt and 72 hpt, respectively. In our following iTRAQ studies, the samples that were harvested at 12 hpt and 72 hpt were used to profile the early and late SA-responsive proteins, respectively.

### SA-induced proteomic changes in the leaves of cucumber seedlings

Using iTRAQ combined with nano-LC-MS/MS technology, the SA-induced proteome changes in cucumber leaves were quantitatively assayed. A total of 241,623 MS/MS spectra were obtained. By searching the cucumber_v2 protein database, 40,043 of 241,623 MS/MS spectra were matched to 9,168 unique peptides, which finally corresponded to 1,821 unique proteins. All of the identified proteins are listed in S2 Table.

**Fig 2:**
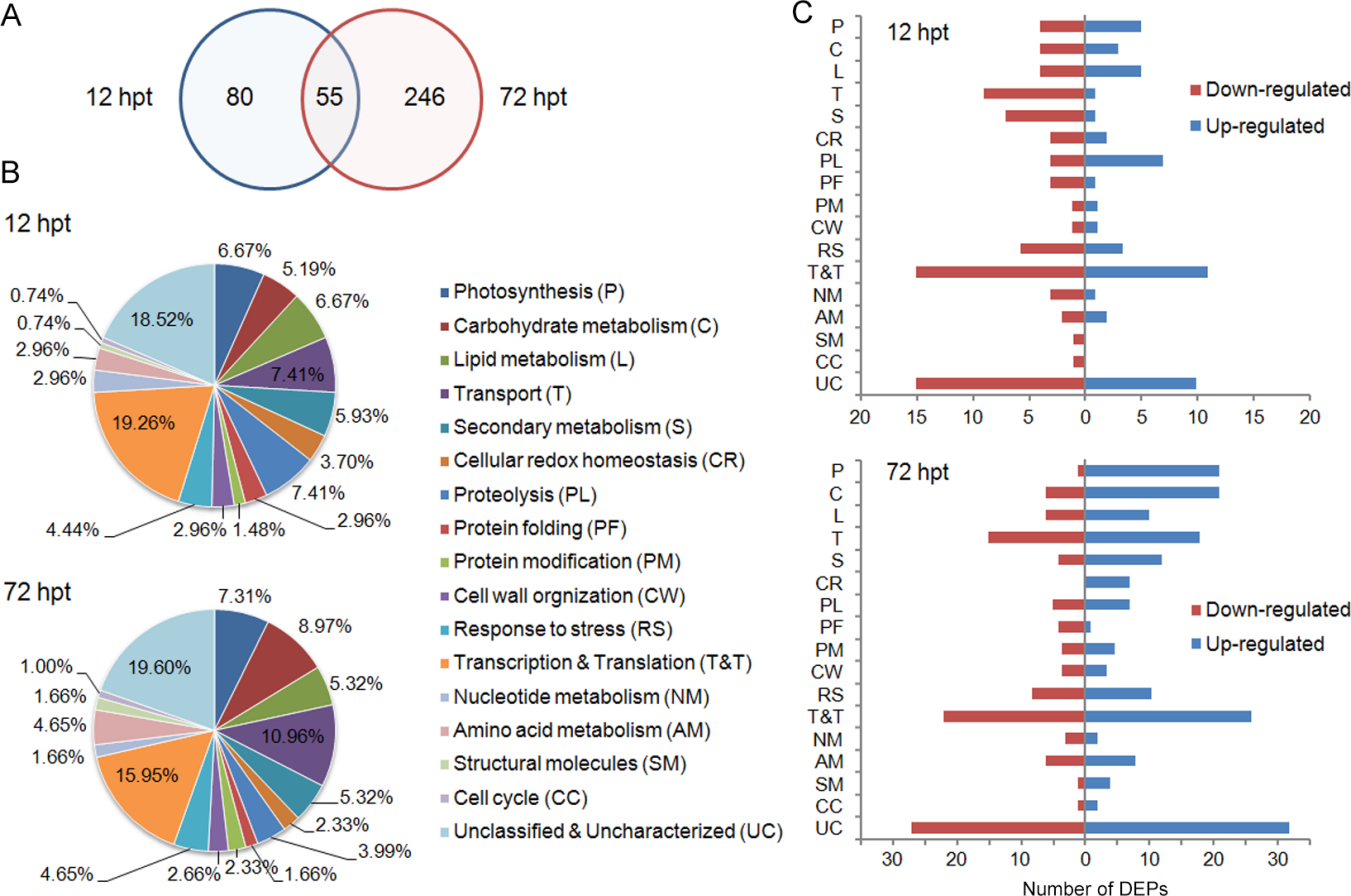
Distribution and functional classification of the 381 SA-responsive DEPs. (A) The numbers of SA-responsive DEPs that were identified at 12 hpt, 72 hpt or both time points are shown in the Venn diagram. (B) Functional classification and distribution of the DEPs via the GO and KEGG databases. (C) The number of DEPs that were up-regulated or down-regulated is also given for each category. The abbreviation for each functional category is indicated in (B). hpt, hours post treatment.

Among the 1,821 identified proteins, 1,810 identities were successfully quantified (S2 Table). Although the direction of protein change is correct, the iTRAQ assay always leads to fold-change underestimation (for both up- and down-regulation) [32]. Therefore, in this study, we used 1.5-fold and *P* < 0.05 cut-offs to assess the SA-induced changes in protein abundance, as mentioned in most previous reports [33–36]. Based on these criteria, 381 proteins were defined as differentially expressed proteins (DEPs) upon SA treatment (as listed in S3 and S4 Tables). Of these DEPs, 135 and 301 DEPs were identified at 12 hpt and 72 hpt, respectively (Fig 2A).

Based on the Gene Ontology (GO, http://www.geneontology.org) and the Kyoto Encyclopedia of Genes and Genomes (KEGG, http://www.genome.jp/kegg/kegg2.html) pathway analysis, the 381 DEPs fell into 17 major categories according to their biological functions and associated biochemical pathways (Fig 2B; S3 and S4 Tables). The largest category included proteins that are related to the transcriptional and translational processes; 26 and 48 proteins were identified at 12 hpt and 72 hpt, respectively. This result was partially due to the transcriptional and translational apparatus being highly dynamic and possibly affected by environmental signals. In addition to proteins that are related to transcription and translation, at 12 hpt, the five most abundant categories included proteins that are involved in proteolysis (7.41%), transport (7.41%), lipid metabolism (6.67%), photosynthesis (6.67%), and secondary metabolism (5.93%). At 72 hpt, the five largest categories were transport (10.96%), carbohydrate metabolism (8.97), photosynthesis (7.31%), secondary metabolism (5.32%), and lipid metabolism (5.32%) (Fig 2B).

For the DEPs that were identified at 12 hpt, the number of proteins that were down-regulated (81 DEPs) was larger than that of proteins that were up-regulated (54 DEPs). In particular, for the proteins that are involved in transport, secondary metabolism, protein folding, and cell wall organization, more than 80% of the DEPs were down-regulated. However, in the category of proteolysis, seven of ten DEPs were up-regulated (Fig 2C). At 72 hpt, of the 301 identified DEPs, 189 were up-regulated, and 112 were down-regulated. Of note, in the categories of photosynthesis, carbohydrate metabolism and lipid metabolism, more than half of the DEPs were up-regulated (Fig 2C).

### Transcriptional expression analysis of DEPs by qRT-PCR

**Fig 3:**
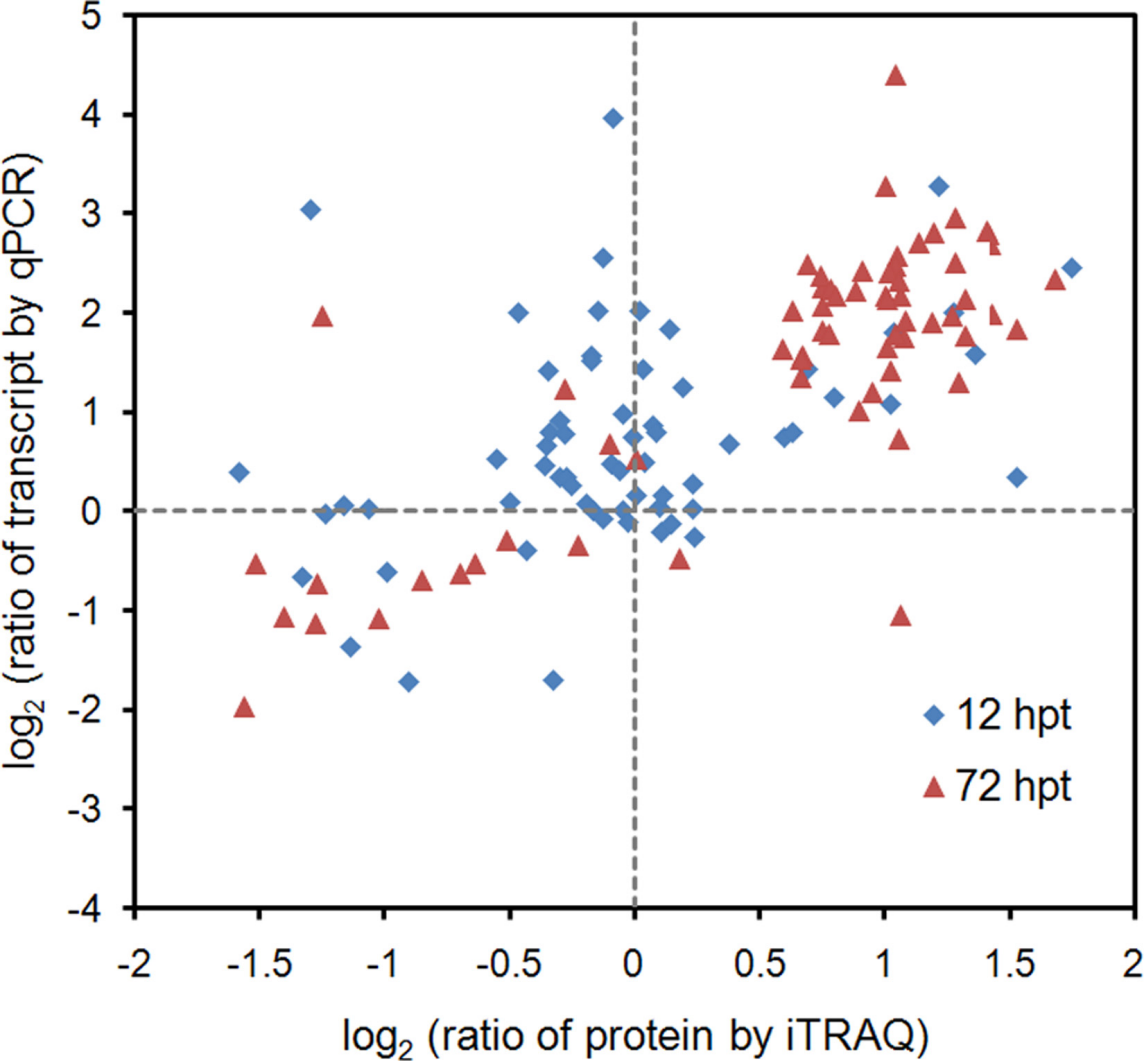
Comparison of the expression ratios from qRT-PCR (y-axis) and iTRAQ (x-axis) profiling. Log_2_ expression ratios were calculated from SA-treated samples vs control. hpt, hours post treatment. The data represent the means of three replicates.

In order to further understand the correspondence between proteins and the mRNA expression patterns of the SA-responsive DEPs,64 proteins were selected to assay their time courses of SA-induced transcriptional changes by qRT-PCR (Fig 3;). The selected DEPs were involved in photosynthesis and chloroplastic redox homeostasis, carbohydrate metabolism, lipid metabolism, and secondary metabolism.

By comparing the expression profiles from qRT-PCR and iTRAQ, the results indicated that most of the identified DEPs displayed similar changing profiles at the protein and mRNA levels (Fig 3), further confirming the SA-induced changes at the protein level in our iTRAQ assay. However, the expression ratios from qRT-PCR and iTRAQ showed relatively poor agreement at 12 hpt, especially for some down-regulated DEPs (Fig 3), such as 14 kDa thylakoid membrane phosphoprotein (TMP14), chloroplastic 3-oxoacyl-ACP synthase II (KARII), chloroplastic enolase 1 (chENO1), and mitochondrial electron transfer flavoprotein subunit alpha (ETFα). These DEPs did not show any significant change at the transcript level (S1–S4 Figs). The possible reason for this discrepancy might be brought by the increased proteolysis, where 70% DEPs were up-regulated at 12 hpt with SA (Fig 2C).

Based on the results from iTRAQ and qRT-PCR, SA might have a significant impact on biochemical processes in cucumber seedlings; the effects were quite distinct at 12 hpt and 72 hpt. The functional protein groups of “photosynthesis”, “redox homeostasis”, “lipid metabolism”, and “secondary metabolism” are described herein. The remaining functional groups, including “Transporter”, “Proteolysis, protein folding and modification”, “Cell wall organization”, “Stress responses”, “Nucleotide and amino acid metabolism”, and “Unclassified and uncharacterized proteins”, are described and discussed in the S1 Text. It should be mentioned that the potential functionality of the DEPs described here is based on homology to proteins from other plants with known function. The actual function of these proteins in cucumber is yet to be determined.

### Effects of SA on photosynthesis and chloroplastic redox homeostasis

At 12 hpt, most of the DEPs that are involved in photosynthetic electron transfer, such as NAD(P)H-quinone oxidoreductase subunit N (NDHN), ferredoxin-2 (Fd-2), oxygen-evolving enhancer proteins (OEEs), cytochrome b_6_f complex iron-sulfur subunit (Fe-S), and plastocyanin (PC), were up-regulated. However, the enzymes that are involved in CO_2_ fixation did not show any significant change at this time point. Up-regulation was detected until 72 hpt for Calvin cycle enzymes, including Rubisco large-subunit N-methyltransferase (LSMT), Rubisco activase 1 (RA1), Rubisco small chain (RbcS), Calvin cycle protein CP12-2, and fructose-1,6-bisphosphatase (FBP) (S1 Fig). The iTRAQ results were in agreement with our finding that *P*_n_ was significantly elevated in cucumber seedlings until 60 h after SA treatment (Fig 1C).

**Fig 4:**
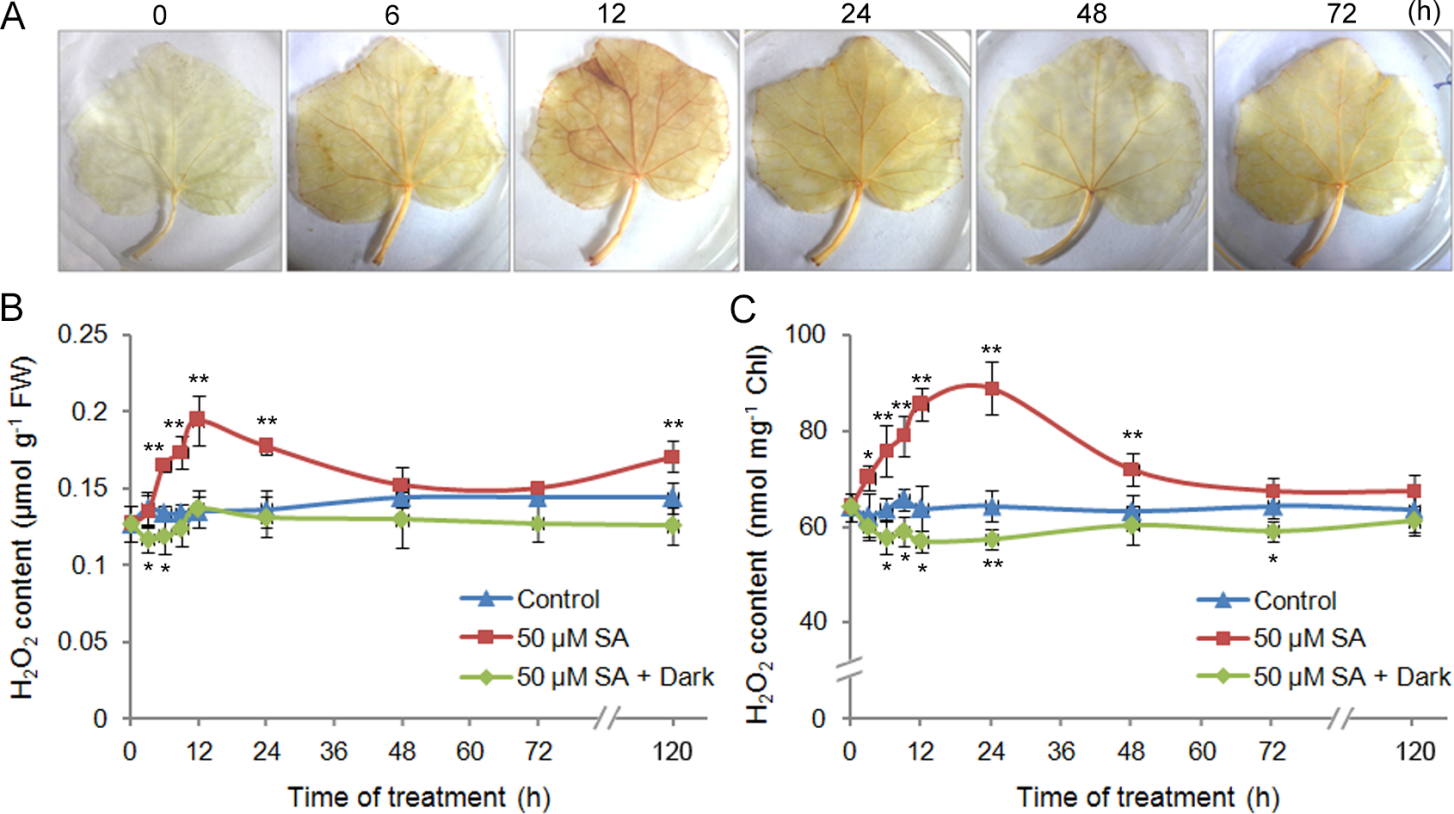
Changes in the H_2_O_2_ contents in response to SA in the first leaves of cucumber seedlings. (A) *In situ* detection of H_2_O_2_ by DAB staining. (B, C) Quantitative determination of total (B) and chloroplastic (C) H_2_O_2_ levels under light and dark conditions. The data represent the means of three replicates ± SD. *P < 0.05 and **P < 0.01 compared to the corresponding value from the control.

The mismatch between the light reaction and photosynthetic CO_2_ fixation at 12 hpt indicated that the absorbed light energy was mainly converted into some other forms of energy rather than being used for chemical fixation. In plants, the reaction centres of photosystem I (PSI) and PSII in chloroplast thylakoids are the major generation sites of reactive oxygen species (ROS) [37]. Oxygen in PSI can be reduced to superoxide anion radical (^-^O_2_), which is spontaneously disproportionated or enzymatically converted to H_2_O_2_ by superoxide dismutase (SOD). In addition, in PSII, oxygen in the ground state can also be excited to the singlet state (^1^O_2_) [37]. During the early SA responses, excess light energy might lead to the formation of ROS. Consistent with this hypothesis, we detected a transient H_2_O_2_ elevation in cucumber leaves at 12 h after SA treatment (Fig 4A and 4B); in chloroplasts in particular, H_2_O_2_ accumulated during 12 h~24 h of SA treatment (Fig 4C). Furthermore, the SA-induced H_2_O_2_ accumulationwas abolished under dark condition, indicating the light-dependency of ROS production upon SA treatment (Fig 4B and 4C).

At 72 hpt, the SA-induced ROS were scavenged to prevent oxidative damage (Fig 4). Two probable glutathione S-transferases and one selenoprotein T protein were up-regulated (S1 Fig). These proteins are involved in ROS scavenging. In particular, a chloroplastic SOD, peroxidase 2 (POD2), and two thioredoxins (TrxM4 and TrxY1) also increased at 72 hpt, suggesting that the accumulated ROS were scavenged in chloroplasts during this time, consistent with the results of the H_2_O_2_ assay (Fig 4C).

### Effects of SA on carbohydrate and energy metabolism

At 12 hpt, only seven DEPs involved in carbohydrate metabolism were identified, and these proteins were dispersed across different metabolic steps. However, at 72 hpt, 27 DEPs were identified, and three proteins related to sucrose metabolism showed increased levels. One of these proteins was sucrose phosphate synthase 2 (SPS2), a key enzyme for sucrose synthesis via sucrose-6′-phosphate. The other two DEPs were raffinose synthases (RFS) (S2 Fig). RFS can transform sucrose to raffinose, the synthetic precursor of stachyose. In cucumber, stachyose is the most common form of carbohydrate that is transported in the phloem [38]. Consistent with the iTRAQ findings, a stachyose synthase (STS) was up-regulated, and the contents of both raffinose and stachyose increased in cucumber leaves after 3 d of 50 μM SA treatment [23, 26]. These results suggest that SA might facilitate stachyose synthesis and thereby sugar transport in cucumber seedlings.

Different from that of sucrose, starch metabolism was segregated in the plastids (S2 Fig) [39]. Two enzymes that are responsible for starch synthesis from hexose phosphates were up-regulated at 72 hpt, glucose-1-phosphate adenylyltransferase small subunit (AGPS) and 1,4-alpha-glucan-branching enzyme 2–2 (SBE2.2), indicating that SA might increase starch synthesis at 72 hpt. Starch degradation was triggered by removal of the phosphate groups at the surface of starch granule by dual-specificity protein phosphatase 4 (DSP4, also designated as starch-excess 4, SEX4) [40]. DSP4 increased at 72 h after SA treatment. Two additional enzymes that are involved in starch degradation, disproportionating enzyme (DPE) and pullulanase 1 (PU1), were also up-regulated by SA (S2 Fig). However, α-1,4 glucan phosphorylase (α-GP), which catalyzes the reversible phosphorolysis of glucan chains and releases glucose-1-phosphate for starch resynthesis [41], was down-regulated. Taken together, these results suggest that SA might enhance starch synthesis and subsequent degradation, but it does not do so for storage.

Hexoses can be converted into organic acids by the glycolysis and tricarboxylic acid (TCA) cycle. In this study, we identified nine DEPs that are involved in glycolysis, including hexokinase-1 (HK1), pyrophosphate-fructose 6-phosphate 1-phosphotransferase subunit alpha (PFP-α), glyceraldehyde-3-phosphate dehydrogenase (GAPDH), chloroplastic glucose-6-phosphate isomerase 1 (chGPI1), chloroplastic phosphoglycerate mutase (chPGM), chloroplastic enolase 1 (chENO1), NADP-dependent malic enzyme (NME) and two phosphoenolpyruvate carboxylases (PEPC and PEPC4). In addition to chENO1, these enzymes were up-regulated at 72 hpt (S2 Fig). Discordantly, two enzymes that are involved in the TCA cycle, malate dehydrogenase (MDH) and mitochondrial isocitrate dehydrogenase catalytic subunit 5 (ICDH), were down-regulated at 72 hpt. Consistent with this pattern, two components of mitochondrial respiratory electron transport, quinone oxidoreductase (QOR) and ATP synthase subunit alpha 2 (ATPase-α2), were also down-regulated at 72 hpt (S2 Fig). The above results indicate that SA might enhance glycolysis but inhibit respiration by uncoupling glycolysis, the TCA cycle and respiratory electron transport.

Additionally, we identified some components that are involved in the glucuronate pathway and pentose-phosphate shunt. UDP-glucuronate 4-epimerase 6 (GAE6) is the key point of the glucuronate pathway; it was up-regulated at both 12 hpt and 72 hpt (S2 Fig). The pentose-phosphate shunt is an alternative pathway for glucose oxidation and can provide a large amount of reducing equivalent NADPH to maintain the cellular redox potential [42]. Consistent with the SA-induced changes of ROS (Fig 4), two enzymes in the pentose-phosphate shunt, ribose-5-phosphate isomerase (RPI) and xylulose kinase (XK), were down- and up-regulated at 12 hpt and 72 hpt, respectively (S2 Fig).

### Effects of SA on lipid metabolism

Upon SA treatment, nine and sixteen proteins that are involved in fatty acid and lipid metabolism were significantly changed at 12 hpt and 72 hpt, respectively. In the biosynthesis of fatty acids, two 3-oxoacyl-[acyl-carrier-protein] reductases (KAR and KARII) were up-regulated at 12 hpt. At 72 hpt, in addition to the two KARs, another three proteins, including acyl carrier protein 1 (ACP1), enoyl-ACP reductase [NADH] (ENR), and 10-kDa acyl-CoA-binding protein-like isoform 1 (ACBP1), were up-regulated by SA (S3 Fig). These results indicate that SA might enhance the *de novo* synthesis of fatty acids.

In chloroplasts, the synthesized acyl-ACPs can be assembled with glycerol-3-phosphate to form lipids, including phospholipids, sulfolipids, and galactolipids. This reaction is catalyzed by glycerol-3-phosphate acyltransferase (GPAT), which was down-regulated by SA at 12 hpt (S3 Fig). The cucumber GPAT showed higher homology to the squash GPAT than to those from Arabidopsis and spinach (S5 Fig). The squash GPAT could selectively incorporate the saturated acyl-ACPs into chloroplast membranes and hence affect the membrane fluidity of aerial tissues and make plants sensitive to chilling [43, 44]. In cucumber, the down-regulation of GPAT by SA could ensure the incorporation of unsaturated fatty acids into the chloroplastic lipids, thereby maintaining the degree of unsaturation of chloroplast membranes. GDP-mannose-dependent alpha-mannosyltransferase (also termed sulfoquinovosyl transferase, SQD2), which is responsible for the synthesis of sulfolipids, was down-regulated at both transcript and protein levels at 72 hpt (S3 Fig). However, the protein trigalactosyldiacylglycerol 4 (TGDG4), which is involved in galactolipid synthesis, was up-regulated at 72 hpt. Changes in these proteins indicate that SA might modulate the assembly of lipids in chloroplastic membranes.

Furthermore, chloroplastic lipids are a rich resource of linolenic acid (18:3), which is the major synthetic precursor of jasmonate (JA). Allene oxide synthase (AOS) is the first specific enzyme and the major control point of the JA biosynthetic pathway [45]. AOS was up-regulated by SA at 12 hpt but down-regulated at 72 hpt. A similar SA-responsive pattern was also found at the transcription level (S3 Fig). The changes in AOS suggest a transient induction and subsequent reduction of JA in response to SA in cucumber leaves. In contrast, another protein that is potentially associated with JA biosynthesis, linoleate 13S-lipoxygenase 2–1 (LOX2.1), was markedly induced at 72 hpt (S3 Fig). SA-induced LOX activity might potentially be involved in defence mechanisms but not JA synthesis.

In the cytosol, acyl-ACPs can be converted into acyl-CoAs and then loaded into peroxisomes for β-oxidation. In this study, acyl-CoA oxidase (ACX), the first enzymethat is involved in peroxisomal β-oxidation [46], was up-regulated by SA at 72 hpt (S3 Fig). Similarly, the peroxisomal fatty acid β-oxidation multifunctional protein (MFP) was also up-regulated at 72 hpt. Consistent with these findings, trans-2-enoyl-CoA reductase (TER), which catalyzed the irreversible ACX reaction [47], was down-regulated at 12 hpt (S3 Fig). The changes in these proteins indicate that peroxisomal fatty acid β-oxidation could be enhanced by SA in cucumber leaves.

Moreover, in the plasma membrane, SA could up-regulate a phosphoinositide phospholipase C2 (PI-PLC2) and thus potentially enhance the hydrolyzation of phosphatidylinositol-4,5-bisphosphate (PIP_2_) into inositol-1,4,5-triphosphate [Ins(1,4,5)P_3_] and diacylglycerol (DAG) (S3 Fig), both of which can function as the secondary signals in plant cells.

### Effects of SA on secondary metabolism

In addition to primary metabolism, SA also had an impact on secondary phenylpropanoid metabolism in cucumber leaves. Lignin, as a phenylpropanoid product, is composed of three basic subunits: *p*-hydroxyphenyl (H), guaiacyl (G), and syringyl (S) monolignols [48]. In this study, four enzymes that are required for monolignol synthesis were identified, including cinnamoyl-CoA reductase 2 (CCR2), caffeic acid 3-*O*-methyltransferase (COMT), caffeoyl-CoA *O*-methyltransferase (CCOMT), and aldehyde dehydrogenase family 2 member C4 (ALDH2C4). All of these enzymes were substantially up-regulated at both the transcript and protein levels at 72 hpt (S4 Fig), indicating enhanced lignification upon SA. In particular, ALDH2C4 was identified as a potential branch-point enzyme catalyzing the back oxidation of coniferaldehyde and sinapaldehyde to yield the corresponding hydroxycinnamates [49, 50]. The closest homologue of cucumber ALDH2C4 was REF1 from Arabidopsis (72.03%, S6 Fig), which has a substrate preference for sinapaldehyde over coniferaldehyde [51]. Additionally, two key Gly residues (G158 and G422) in the sinapaldehyde dehydrogenase activity of REF1 were also conserved in cucumber ALDH2C4 (S6 Fig). These findings indicate that SA might lead to the enhanced back conversion of sinapaldehyde to sinapate and subsequently result in a reduction of the relative proportion of the S-monolignol subunit.

Alternatively, *p*-coumaroyl CoA produced in the phenylpropanoid pathway can also be used as a substrate for flavonoid biosynthesis. Chalcone isomerase 2 (CHI2), which catalyzes the stereospecific isomerization of chalcones into the corresponding flavanones, was up-regulated at 72 hpt at both the mRNA and protein levels (S4 Fig). Additionally, two enzymes that are involved in the degradation of phenylalanine and its precursor chorismate were identified, including primary amine oxidase (AOC) and pterin-4-alpha-carbinolamine dehydratase (PCD). These enzymes were down-regulated at 12 hpt and 72 hpt, respectively (S4 Fig), consistent with the conclusion that SA might enhance the phenylpropanoid pathway.

Additionally, some SA-responsive DEPs were involved in the metabolism of chlorophylls (S3 and S4 Tables). At 12 hpt, chloroplastic chlorophyll synthase was up-regulated, while the enzyme that was involved in chlorophyll degradation, protoporphyrinogen oxidase, was down-regulated. At 72 hpt, the three identified DEPs that are involved in the chlorophyll biosynthesis, including carotene ε-monooxygenase (LUT1), chromoplast-specific carotenoid-associated protein (CHRC), and red chlorophyll catabolite reductase (RCCR), were up-regulated. These results indicate that SA might increase the chlorophyll levels, in agreement with the enhanced photosynthesis.

Concerning hormone metabolism, five DEPs were identified. One DEP was 1-aminocyclopropane-1-carboxylate oxidase homologue 3 (ACO3), an ethylene-forming enzyme. ACO3 was down-regulated at 12 hpt with SA. The other two DEPs were putative IAA-amino acid hydrolases (ILR1 and ILR1-4) and were repressed at both 12 hpt and 72 hpt. Consistent with this result, two putative UDP-glycosyltransferases (UGT92A1 and UGT85A7), which might convert IAA to its inactive glucosides, were up-regulated by SA at 72 hpt (S4 Table), indicating an inhibitory effect of SA on auxin signalling in cucumber leaves.

## Discussion

SA is an important plant hormone and has great agronomic potential in promoting the growth and enhancing the stress tolerance of agricultural crops; however, studies on its working mechanisms are rare. In the present study, using a high-throughput iTRAQ assay, 135 and 301 SA-responsive DEPs were identified in the leaves of cucumber seedlings, and revealed distinct physiological changes during the early- and late-responsive phases (Figs 1 and 2). In the previous 2-DE assays, no more than 70 DEPs were identified from cucumber or other plant species [14, 16, 26] (S7 Fig). By comparing to these gel-based reports, our present iTRAQ assay provided higher proteome coverage. Also, these previous reports only focused on a single time point after SA treatment, while in this study, the proteome data at 12 hpt and 72 hpt could explain the dynamic changes that occur during SA responses.

### Early responses to SA in cucumber leaves

During the early responsive phase, SA exerted its major effects on photosynthetic systems. Upon SA treatment, in spite of enhanced electron transport via the up-regulation of the related proteins, no significant increase in photosynthesis was detected (Fig 1C). In plants, the dissipation of excess excitation energy occurs through thermal irradiation. However, prolonged exposure to such conditions can result in an increase in the generation of ROS [52, 53]. Here, such excess energy led to a significant increase of ROS in chloroplasts (Fig 4). The promoted ROS production by SA has been reported in several stress models. How can SA promote ROS production? Early reports showed the SA-mediated inhibition of ROS-scavenging enzymes, including catalase and cytosolic ascorbate peroxidase [54–56]. Another important ROS resource is NADPH oxidase. However, accumulating evidence has suggested that SA-mediated ROS production is independent of NADPH oxidase [25, 57]. Here, the SA-induced ROS was concluded to be generated from the imbalance between the photosynthetic electron transport and carbon fixation reaction, and this conclusion was confirmed by the light-dependency of ROS generation (Fig 4). The similar ROS origination was also reported in tobacco plants infected with TMV, wherein a rapid shutdown of carbon fixation preceded the ROS generation in chloroplasts under illumination, while the plants that were kept in the dark did not accumulate H_2_O_2_ in chloroplasts [53]. Actually, many abiotic stresses, such as salinity, drought, and temperature, trigger ROS production mainly at the photosynthetic electron chains in chloroplasts [58].

During the early phase, SA also affected fatty acid biosynthesis and lipid assembly. SA could up-regulate the enzymes that are involved in fatty acid synthesis. Fatty acids can serve as major energy reserves by being activated to form CoA derivates and oxidized in peroxisomes. Fatty acid oxidation would be up-regulated during the late phase. Additionally, linolenic acid (18:3), the most abundant fatty acid component, can function as the precursor for JA biosynthesis in chloroplasts. During the early responsive phase, via the modulation on AOS protein, SA might transiently increase the synthesis of JA in cucumber leaves. Consistent with this conclusion, transient JA accumulation had been detected by GC-MS in SA-treated sorghum seedlings [11]. The modest and transient increase in JA induced by SA might be a mechanism to activate a broader range of genes than by SA alone. Fatty acids can be assembled into lipids. In the context of lipid assembly, the SA-induced down-regulation of GPAT was found. GPAT can selectively incorporate saturated fatty-acyl chains into chloroplastic lipids [43]. The down-regulation of GPAT allows plants to maintain the unsaturation degree of membrane lipids, consistent with the GC-MS result that relative contents of unsaturated fatty acids were increased in the flax seedlings treated with SA [59]. This might be an adaptive mechanism for subsequent environmental stresses [43, 44].

It should be noted that, by comparing our results from iTRAQ and qRT-PCR, the SA-induced changes in DEPs during the early phase, especially in the down-regulated DEPs, mainly occurred at the protein level. SA down-regulated the proteins that are involved in protein processing but up-regulated the proteins that are related to proteolysis (S1 Text, “Proteolysis, protein folding and modification”). Pathogen infection or exogenous SA treatment can induce the expression of genes that are involved in intracellular proteolysis [60]. Additionally, SA-induced ROS might function as the second signal for the proteolysis [61].

### Late responses to SA in cucumber leaves

Based on the iTRAQ data, most of the cellular metabolic processes presented significant changes during the late-responsive phase. SA could enhance photosynthesis by up-regulating both light and dark reactions at 72 hpt (Fig 1C; S1 Fig). Consistent with our findings, SA has been established as an enhancer in photosynthesis [62]. Enhanced photosynthesis increases plant growth [26]. Meanwhile, enhanced CO_2_ fixation can avoid the excess excitation energy to be converted into ROS. Consistent with this, the SA-induced ROS burst was scavenged during the late-responsive phase (Fig 4). SA also induced antioxidant enzymes in the chloroplast for ROS scavenging, including SOD, POD, Trx and GST proteins (S1 Fig), consistent with the previous reports [25, 63]. In addition, SA could up-regulate the enzyme for lutein biosynthesis, LUT1 (S4 Table, “5.2 Chlorophyll metabolism”). Lutein is the most abundant carotenoid in plant leaves and can effectively quench ^1^O_2_ produced in PSII [64, 65]. Although the dual redox effects of SA have been well reviewed [58], our iTRAQ data would enrich the relations between SA and ROS.

Consistent with the enhanced photosynthetic CO_2_ fixation, SPS2-mediated sucrose synthesis was induced by SA until 72 hpt. There are three main metabolic fates for the synthesized sucrose. First, sucrose can be transformed to raffinose and stachyose for phloem loading and can be transported to sink tissues [38]. SA can up-regulate the enzymes that are involved in this process, thereby facilitating sugar transport in cucumber seedlings (S2 Fig). The second fate of sucrose is for storage as starch. After SA treatment, two enzymes related to starch synthesis (AGPS and SBE2.2) were up-regulated. However, the subsequent starch degradation was also enhanced by SA, indicating that the fate of starch was not for storage but for degradation. Actually, a detailed biochemical assay for the effects of SA on carbohydrate metabolism was performed in our previous report, and the results confirm our iTRAQ findings [23]. The third fate is to be broken into hexoses for glycolysis [66]. Based on the iTRAQ results, SA could up-regulate glycolysis but down-regulate the subsequent TCA cycle and respiratory electron transport (S2 Fig), indicating that SA can inhibit respiration by uncoupling glycolysis, the TCA cycle and mitochondrial electron transport. The impact of SA on mitochondrial function is not a cucumber-specific phenomenon. Similar uncoupling and inhibitory effects have also been reported in tobacco [67], soybean [68], Arabidopsis [69], and even in mammalian cells [70]. In Arabidopsis, SA directly acted on mitochondrial respiratory complex III and inhibited its activity [69].

It would be interesting to note what happens for the carbon flux originated from the glycolysis. In addition to energy production, the intermediate organic acids that are produced during glycolysis can serve as precursors to generate primary metabolites for amino acid, nucleotide and fatty acid biosynthesis [66, 71]. Consistent with this, proteins that are involved in the biosynthesis of amino acids and nucleotides were up-regulated after SA treatment (S1 Text, “Nucleotide and amino acid metabolism”). These results suggest that SA might alter the carbon metabolic flux towards amino acid and nucleotide biosynthesis but not for respiration.

Additionally, SA could up-regulate the proteins involved in fatty acid biosynthesis and peroxisomal β-oxidation during the late phase. The energy that is generated during β-oxidation might be used to enhance plant growth or to physiologically prime for stress resistance. Moreover, the acetyl-CoA produced in β-oxidation can be converted into hexoses via gluconeogenesis and provides a wide variety of biosynthetic precursors for amino acids, nucleotides, polysaccharides and so on [72]. Additionally, the enzymes that are responsible for sulfolipid synthesis were down-regulated by SA during the late phase, while those for galactolipid synthesis were up-regulated. Sulfolipids are the least prevalent components of the photosynthetic membrane lipids and are dispensable under normal growth conditions [73]. However, for galactolipids, they constitute up to 80% of thylakoid lipids and play critical roles in photosystem structure and function [74]. The up-regulation of galactolipids biosynthesis might contribute to the enhanced photosynthesis upon SA treatment (Fig 1). Certainly, further biochemical evidences would be necessary to fully elucidate the effects of SA on the changes in lipid components.

SA also affects cell wall organization during the late phase (S1 Text, “Cell wall organization”). As the front line for attacking pathogens, multiple changes in the cell wall triggered by microbial attack have been well described [75]. However, the changes in response to SA treatment remain obscure. In this study, proteins for callose synthesis and deposition were up-regulated by SA. The deposition of callose into cell walls can protect cells against water loss and inhibit the development of pathogen haustoria by preventing the pathogen from obtaining nutrients from the host cells [76]. Callose deposition might be one of the priming mechanisms for SA to activate the broad-spectrum stress tolerance. Consistent with our conclusion, Lee *et al*. (2011) identified a plasmodesmata-localized protein (PDLP5) that functions at the interface between SA and callose deposition. The expression of *PDLP5* was highly induced by exogenous SA and eventually induced the accumulation of callose at plasmodesmata and isolated the stressed cells from adjacent cells [77].

Lignin can deposit in the cell walls through covalent cross-linkage to polysaccharides. By up-regulating the enzymes in the phenylpropanoid pathway, SA could enhance the lignification of cell walls, making cell walls more resistant to mechanical pressure and to water and, thus, less accessible to cell-wall-degrading enzymes from pathogens [78]. Furthermore, the greater abundance of ALDH2C4 upon SA treatment might lead to a selective reduction in the relative proportion of the S-monolignol subunit (S4 Fig). This result was quite different from that observed in the leaves that were treated with fungal elicitors, where a specific increase in S-lignin was found [79]. The methoxyl groups in the 5-position of syringyl units in S-monolignol eliminate the potential coupling site, making S-ligninmore linear and less crosslinked [80]. One possibility to decrease the proportions of linear S-lignin upon SA treatment was to cause the cell walls to expand more rapidly, in agreement with the growth-promoting effects of SA in cucumber seedlings as reported previously [26].

Another family of late SA-responsive protein is the proteins involved in stress responses. Unlike the dramatic induction of *PR1-1a* in the cucumber leaves with pathogen infection, the expression of *PR1-1a* gene peaked at 72 hpt, which was consistant with the moderate accumulation of endogenous SA (Fig 1). Similar to PR1-1a, the levels of a putative serine-type endopeptidase (PR-7) was identified to be up-regulated (1.513-fold) until 72 hpt. By induction of these proteins, including dehydrin-like proteins, universal stress proteins, and pyrroline-5-carboxylate reductase (S1 Text, “Stress responses”), SA could regulate the plant-water relations and thereby provide protections in plants against multiple stresses [10].

### A possible SA-responsive network in cucumber leaves

**Fig 5:**
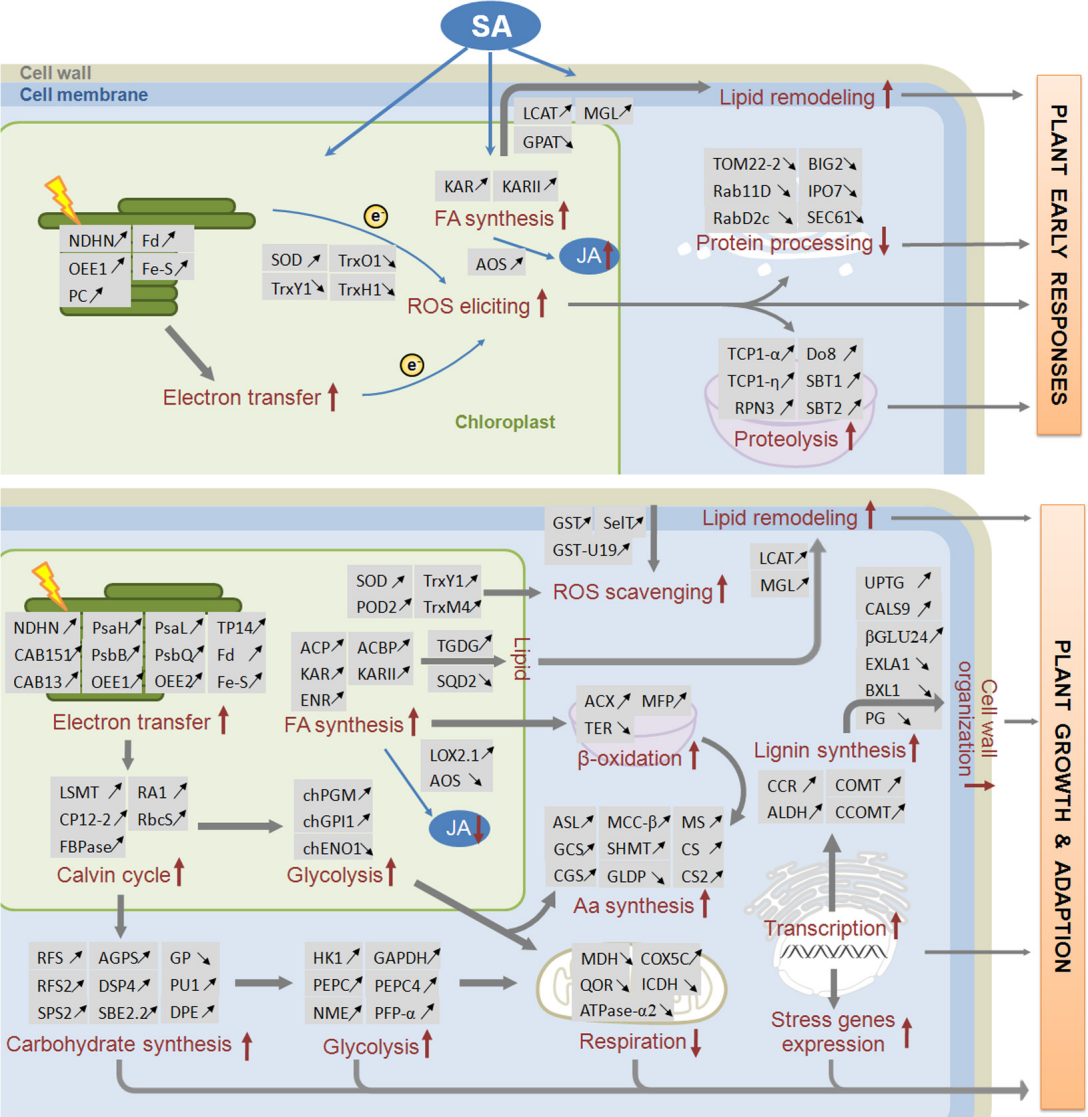
A putative SA-responsive protein network in the leaves of cucumber seedlings. Up- and down-regulation are marked as up and down arrows, respectively. Detailed descriptions are given in the text, and the full name for each abbreviation is available in S3 and S4 Tables.

By combing our proteomic, qRT-PCR, and biochemical evidences, we present a possible SA-responsive network with different molecular events occurring during the early and late SA-responsive phases (Fig 5). During the early phase, SA enhanced the photosynthetic electron transferring in chloroplast but had no effect on CO_2_ fixation. The electrons escaped from the photosynthetic systems to form ROS and thereby induced a cellular ROS burst. Accordingly, the enzymes that are involved in ROS scavenging were repressed by SA. The ROS then functioned as secondary signals. SA also regulated the enzymes that are related to fatty acid biosynthesis and lipid assembly to maintain the unsaturation degree of cellular membranes. By up-regulating AOS, SA could induce a transient increase of JA to broaden the resistance responses. Via the regulation of protein processing and proteolysis, the SA-induced changes of DEPs during the early phase mainly occurred at the protein level. During the late phase, multiple cellular metabolic pathways presented significant changes. By up-regulating both photosynthetic electron transfer and CO_2_ fixation, SA enhanced photosynthesis. Meanwhile, the cytosolic and chloroplastic excess ROS were scavenged by the SA-induced enzymes, including SOD, POD, Trx, and GSTs. SA also increased carbohydrate synthesis and glycolysis but repressed mitochondrial respiration by uncoupling glycolysis, the TCA cycle and oxidative phosphorylation. The enhanced glycolysis and TCA cycle served as the sources of organic acids and supplied the precursors for amino acid and nucleotide biosynthesis. The synthesized fatty acids were also loaded into peroxisomes for β-oxidation, serving as another resource for the intermediate organic acids and energy that are required for enhanced plant growth. SA also enhanced the biosynthesis of lipids, especially galactolipids. Moreover, SA regulated the cell wall organization by increasing polysaccharide synthesis, enhancing lignin and callose deposition, and reducing the relative contents of S-lignin, finally making the cell walls more rigid. The expression of some stress-responsive genes was also induced by SA during the late phase as a preadaptive mechanism.

In summary, the dynamic changes in these SA-responsive DEPs indicate that cells can sense SA signals and initiate cellular responses by modulating the amount of the proteins and the corresponding molecular events. The protein network depicted here could help us to systemically understand the SA-responsive molecular changes, whereas a further biochemical exploration and in-depth functional characterization of these DEPs would be necessary to fully elucidate the molecular mechanisms behind SA responses.

## Conclusions

Applying iTRAQ to study the SA responsiveness allowed the identification and quantification of 1,820 proteins in leaves of cucumber seedlings, and a total of 135 and 301 DEPs were identified at 12 h and 72 h after SA treatment, respectively. These longer lists of proteins complement a previous gel-based proteomic study with better protein coverage and better connect the early- and late-responsive phases by analyzing two time points after SA application. Based on the putative functions and abundance changes of the identified SA-responsive DEPs, we propose a network for SA responsiveness. This network covers a broad series of metabolic processes. During early SA responsiveness, SA elicits ROS production via the regulation of photosynthesis and leads to membrane lipidre modelling. During the late responses, SA confers growth-promoting and adaptive effects on the cell by regulating multiple processes that include photosynthesis, ROS scavenging, carbohydrate/energy metabolism, fatty acid biosynthesis and β-oxidation, lipid assembly and remodelling, amino acid synthesis, and cell wall reorganization. In association with the prior evidence, our study presents an important data resource integrating the molecular events that occurred during SA responsiveness and provides a solid basis for further functional research of single nodes of this SA-responsive network.

## Supporting Information

S1 Fig Overview of SA-responsive DEPs that are associated with photosynthesis and the related ROS homeostasis. The photosynthetic electron transfer (A) and CO_2_ fixation (B) are illustrated. The solid and dashed gray arrows indicate the flow of H^+^ and electron in the thylakoid membranes, respectively. The black arrows indicate the generation and scavenging of ROS. The SA-responsive DEPs identified in iTRAQ assay are marked in red, and the SA-induced folds at mRNA and protein levels are shown in (C).
(DOCX)

S2 Fig Overview of SA-responsive DEPs that are associated with carbohydrate and energy metabolism. The sucrose and starch metabolism (A), glycolysis, pentose-phosphate shunt, glucuronate pathway and TCA cycle (B), and oxidative phosphorylation (C) are included. The DEPs identified in iTRAQ assays are highlighted in red, with the SA-induced folds at mRNA and protein levels are shown in (D).
(DOCX)

S3 Fig Overview of SA-responsive DEPs that are associated with lipid metabolism. (A) Metabolic pathways of fatty acids and lipids with the SA-regulated DEPs being highlighted in red. (B) The relative mRNA and protein changing folds of DEPs in responsive to SA by qRT-PCR and iTRAQ, respectively.
(DOCX)

S4 Fig Overview of SA-responsive DEPs that are associated with phenylapropaniod pathway. (A) The phenylapropaniod pathway responsive to SA, with the identified DEPs being highlighted in red. (B) The relative mRNA and protein changing folds of DEPs in responsive to SA by qRT-PCR and iTRAQ, respectively.
(DOCX)

S5 Fig The cucumber GPAT shows higher homology to the chilling sensitive form in squash. (A) Sequence alignment was performed among the GPAT proteins from cucumber (*Cucumis sativus*, CsGPAT), squash (*Cucurbita moschata*, CmGPAT), Arabidopsis (*A*. *thaliana*, AtGPAT), and spinach (*Spinacia oleracea*, SoGPAT), using Clustal X 1.81, followed by shading with Boxshade 3.21. The gaps are indicated as dashes. (B) Phylogenetic tree of multiple GPAT proteins was constructed using the Neighbor-Joining method with the program MEGA 5.0. The NCBI accession numbers are as follows: SoGPAT (CAA88913), AtGPAT (AEE31448), and CmGPAT (BAB17755).
(DOCX)

S6 Fig Cucumber ALDH2C4 shows high similarity with Arabidopsis REF1 protein. The sequence alignment was performed using Clustal X 1.81 and colored by Boxshade 3.21. The gaps are indicated as dashes. Two conserved Gly residues (G158 and G422) are highlighted in red.
(DOCX)

S7 Fig Venn diagram analyses of the late SA-responsive DEPs from the present study and three other independent studies, including Hao *et al*. (59 DEPs, A), Kundu *et al*. (29 DEPs, B) and Kang *et al*. (62 DEPs, C).
(DOCX)

S1 Table Primer sequences of the genes used in the qRT-PCR. (DOCX)

S2 Table The 1,821 proteins identified in the iTRAQ assay. (XLSX)

S3 Table The 135 SA-responsive DEPs identified at 12 hpt in the iTRAQ assay and their functional classification. (DOCX)

S4 Table The 301 SA-responsive DEPs identified at 72 hpt in the iTRAQ assay and their functional classification. (DOCX)

S1 Text Results and discussion about the functional protein groups “Transporter”, “Protein folding and modification”, “Cell wall organization”, “Stress responses”, “Nucleotide and amino acid metabolism”, and “Unclassified and unidentified proteins”. (DOCX)
