# Supplementary material for: Quantitative Proteomic Profiling of Early and Late Responses to Salicylic Acid in Cucumber Leaves
Source: PLoS One. 2016 Aug 23;11(8):e0161395. doi: 10.1371/journal.pone.0161395 (PMC4995040; doi:10.1371/journal.pone.0161395)
Supplement: S1 Fig — The photosynthetic electron transfer (A) and CO2 fixation (B) are illustrated. The solid and dashed gray arrows indicate the flow of H+ and electron in the thylakoid membranes, respectively. The black arrows indicate the generation and scavenging of ROS. The SA-responsive DEPs identified in iTRAQ assay are marked in red, and the SA-induced folds at mRNA and protein levels are shown in (C). (DOCX) [file pone.0161395.s001.docx]

**Supporting Information**


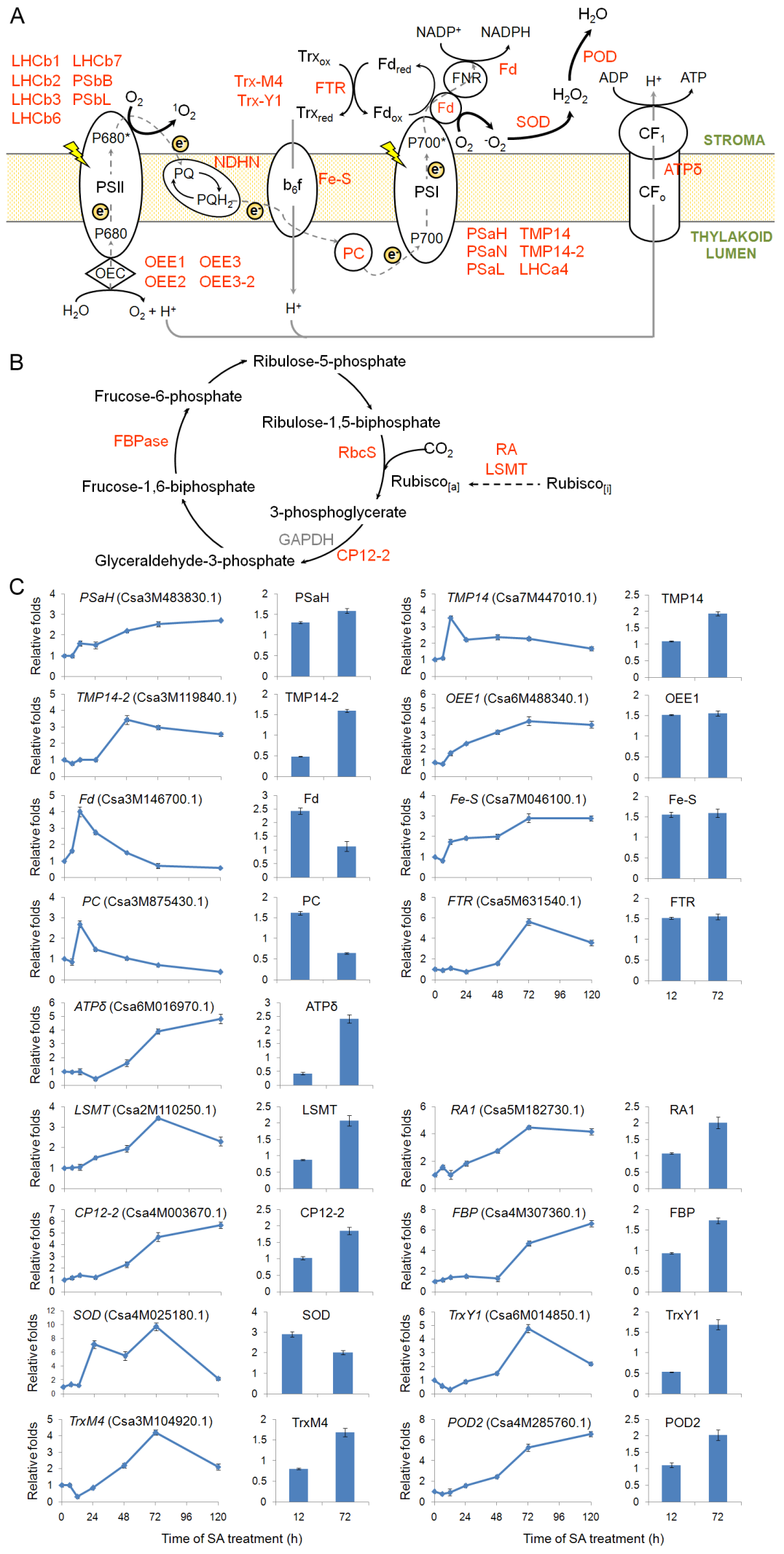


**S1 Fig. Overview of SA-responsive DEPs that are associated with photosynthesis and the related ROS homeostasis.** The photosynthetic electron transfer (A) and CO_2_ fixation (B) are illustrated. The solid and dashed gray arrows indicate the flow of H^+^ and electron in the thylakoid membranes, respectively. The black arrows indicate the generation and scavenging of ROS. The SA-responsive DEPs identified in iTRAQ assay are marked in red, and the SA-induced folds at mRNA and protein levels are shown in (C).
