# Supplementary material for: Quantitative Proteomic Profiling of Early and Late Responses to Salicylic Acid in Cucumber Leaves
Source: PLoS One. 2016 Aug 23;11(8):e0161395. doi: 10.1371/journal.pone.0161395 (PMC4995040; doi:10.1371/journal.pone.0161395)
Supplement: S2 Fig — The sucrose and starch metabolism (A), glycolysis, pentose-phosphate shunt, glucuronate pathway and TCA cycle (B), and oxidative phosphorylation (C) are included. The DEPs identified in iTRAQ assays are highlighted in red, with the SA-induced folds at mRNA and protein levels are shown in (D). (DOCX) [file pone.0161395.s002.docx]

**Supporting Information**


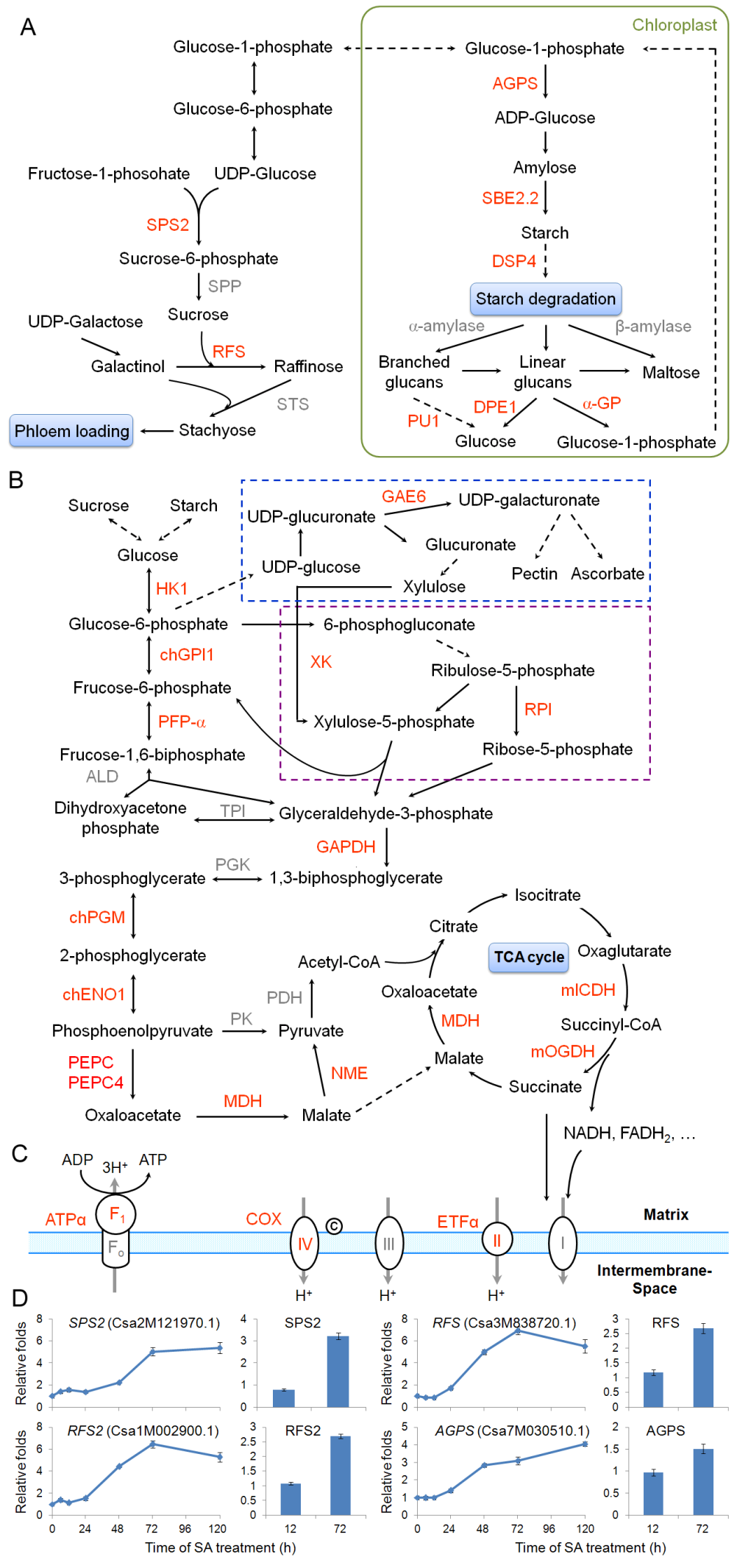


*(S2 Fig continued)*


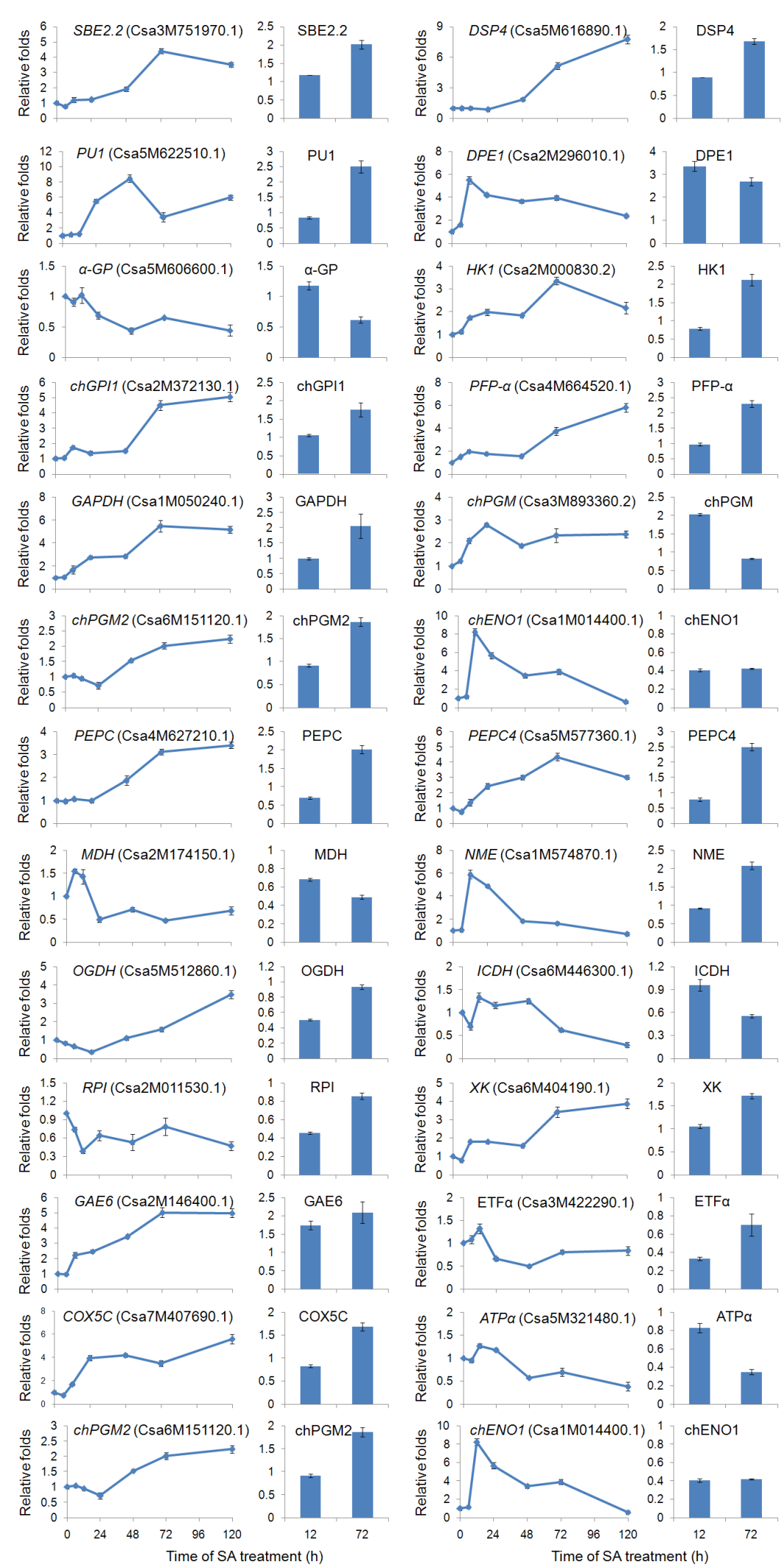


**S2 Fig. Overview of SA-responsive DEPs that are associated with carbohydrate and energy metabolism.** The sucrose and starch metabolism (A), glycolysis, pentose-phosphate shunt, glucuronate pathway and TCA cycle (B), and oxidative phosphorylation (C) are included. The DEPs identified in iTRAQ assays are highlighted in red, with the SA-induced folds at mRNA and protein levels are shown in (D).
