# Supplementary material for: Quantitative Proteomic Profiling of Early and Late Responses to Salicylic Acid in Cucumber Leaves
Source: PLoS One. 2016 Aug 23;11(8):e0161395. doi: 10.1371/journal.pone.0161395 (PMC4995040; doi:10.1371/journal.pone.0161395)
Supplement: S3 Fig — (A) Metabolic pathways of fatty acids and lipids with the SA-regulated DEPs being highlighted in red. (B) The relative mRNA and protein changing folds of DEPs in responsive to SA by qRT-PCR and iTRAQ, respectively. (DOCX) [file pone.0161395.s003.docx]

**Supporting Information**


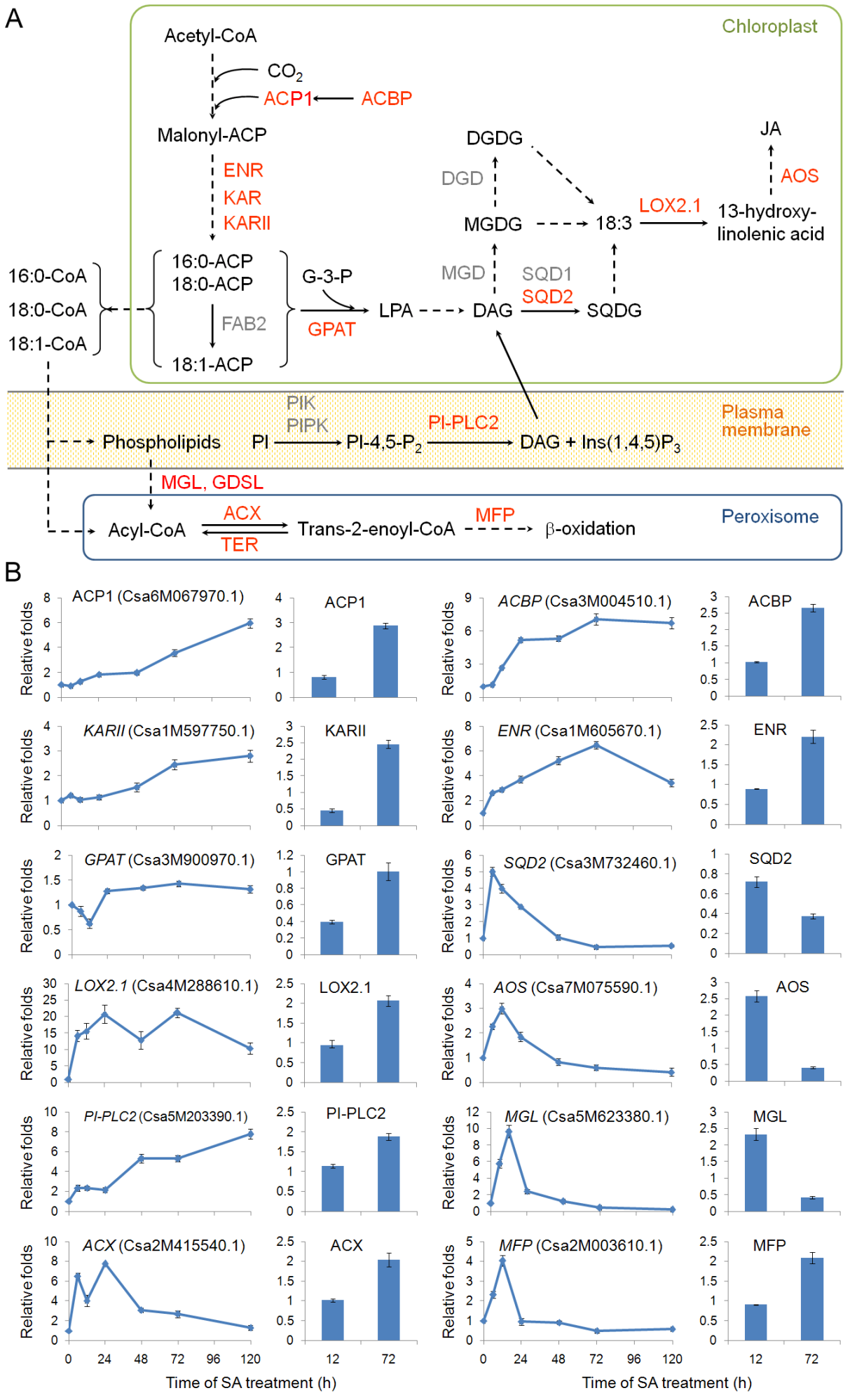


**S3 Fig. Overview of SA-responsive DEPs that are associated with lipid metabolism.** (A) Metabolic pathways of fatty acids and lipids with the SA-regulated DEPs being highlighted in red. (B) The relative mRNA and protein changing folds of DEPs in responsive to SA by qRT-PCR and iTRAQ, respectively.
