# Supplementary material for: Quantitative Proteomic Profiling of Early and Late Responses to Salicylic Acid in Cucumber Leaves
Source: PLoS One. 2016 Aug 23;11(8):e0161395. doi: 10.1371/journal.pone.0161395 (PMC4995040; doi:10.1371/journal.pone.0161395)
Supplement: S4 Fig — (A) The phenylapropaniod pathway responsive to SA, with the identified DEPs being highlighted in red. (B) The relative mRNA and protein changing folds of DEPs in responsive to SA by qRT-PCR and iTRAQ, respectively. (DOCX) [file pone.0161395.s004.docx]

**Supporting Information**


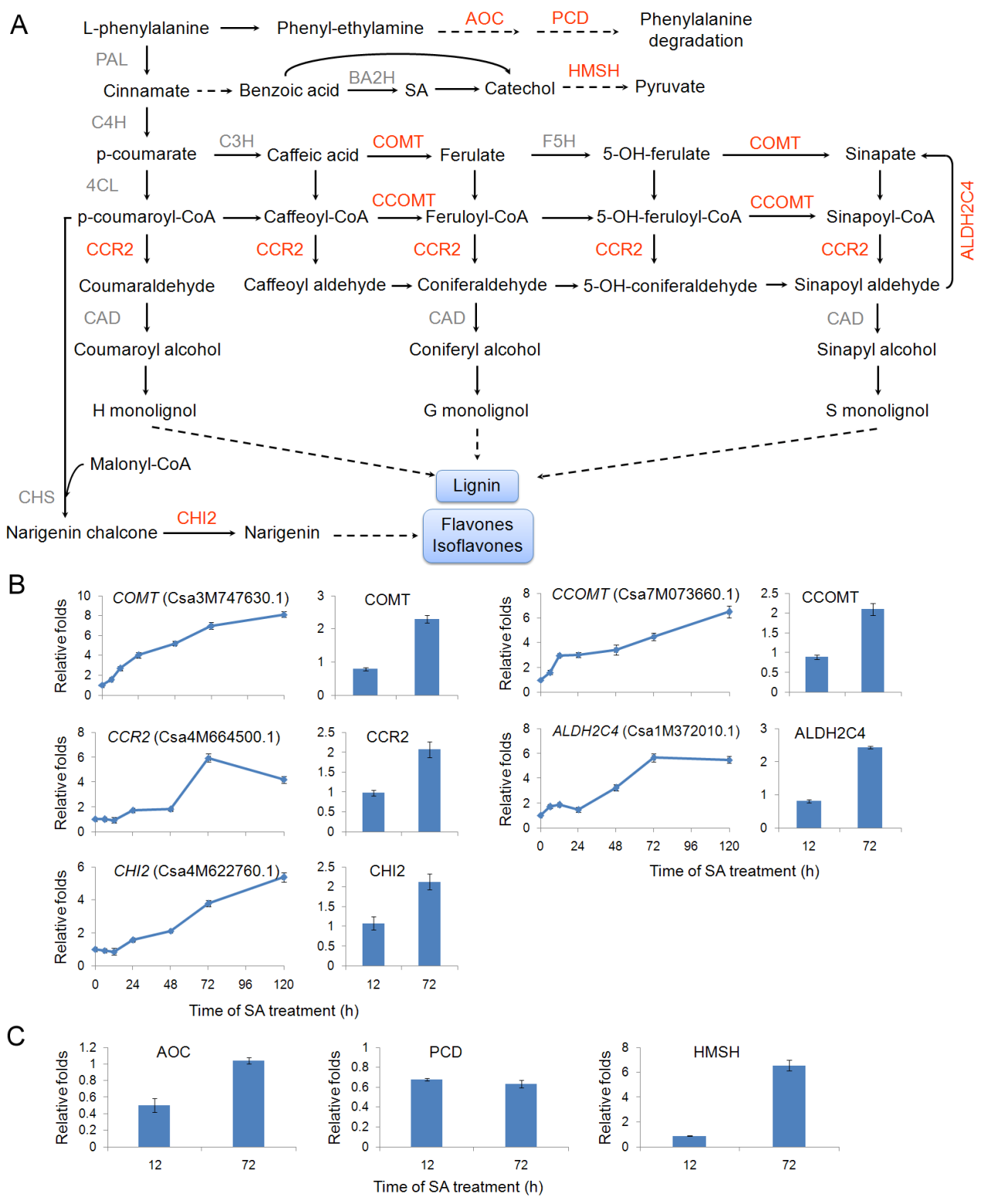


**S4 Fig. Overview of SA-responsive DEPs that are associated with phenylapropaniod pathway.** (A) The phenylapropaniod pathway responsive to SA, with the identified DEPs being highlighted in red. (B, C) The relative mRNA and protein changing folds of DEPs in responsive to SA by qRT-PCR and iTRAQ, respectively.
