# Supplementary material for: Quantitative Proteomic Profiling of Early and Late Responses to Salicylic Acid in Cucumber Leaves
Source: PLoS One. 2016 Aug 23;11(8):e0161395. doi: 10.1371/journal.pone.0161395 (PMC4995040; doi:10.1371/journal.pone.0161395)
Supplement: S5 Fig — (A) Sequence alignment was performed among the GPAT proteins from cucumber (Cucumis sativus, CsGPAT), squash (Cucurbita moschata, CmGPAT), Arabidopsis (A. thaliana, AtGPAT), and spinach (Spinacia oleracea, SoGPAT), using Clustal X 1.81, followed by shading with Boxshade 3.21. The gaps are indicated as dashes. (B) Phylogenetic tree of multiple GPAT proteins was constructed using the Neighbor-Joining method with the program MEGA 5.0. The NCBI accession numbers are as follows: SoGPAT (CAA88913), AtGPAT (AEE31448), and CmGPAT (BAB17755). (DOCX) [file pone.0161395.s005.docx]

**Supporting Information**


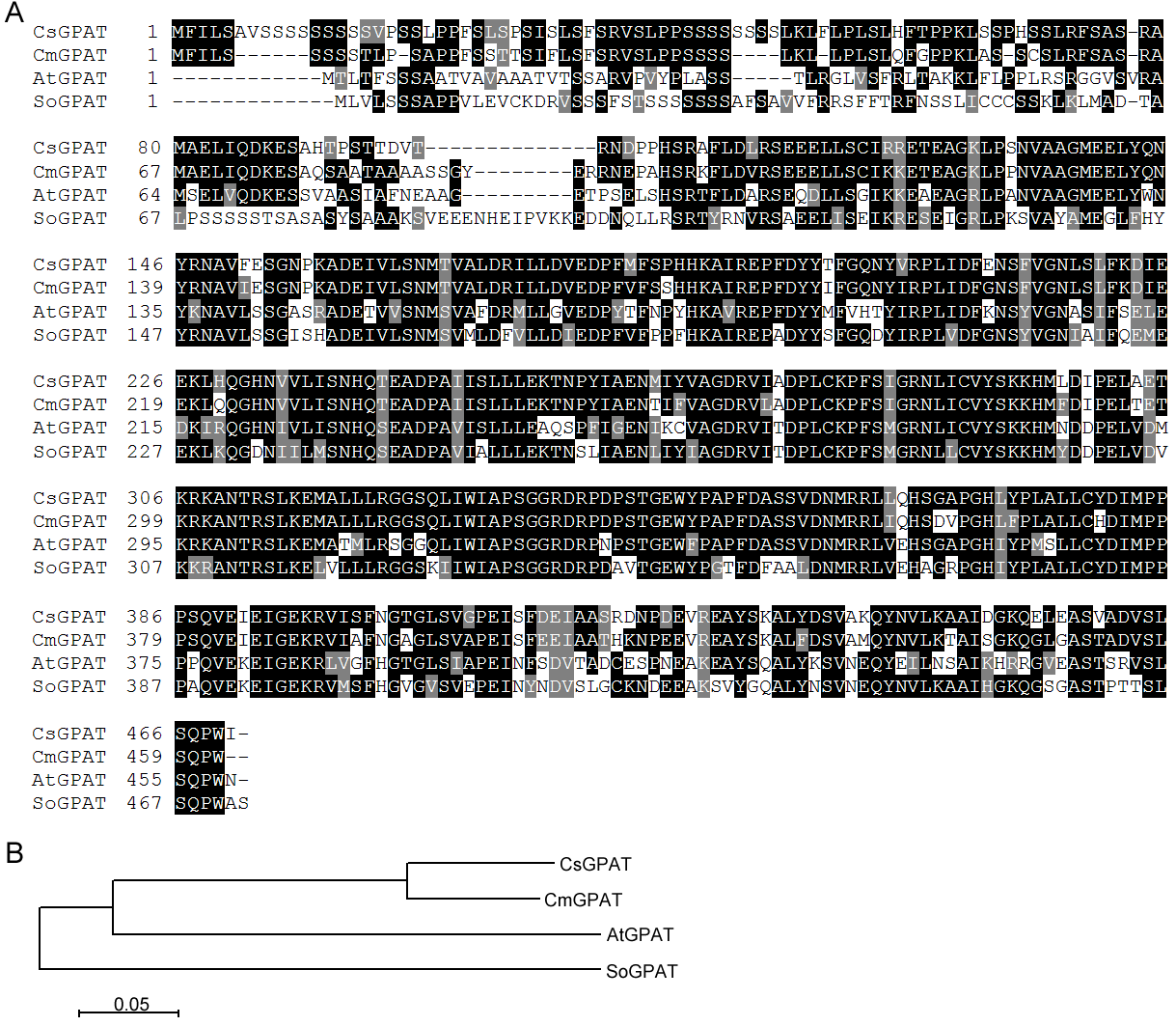


**S5 Fig. Cucumber GPAT shows higher homology to the chilling sensitive form in squash. (A)** Sequence alignment was performed among the GPAT proteins from cucumber (*C. sativus*, CsGPAT), squash (*Cucurbita moschata*, CmGPAT), Arabidopsis (*A. thaliana*, AtGPAT), and spinach (*Spinacia oleracea*, SoGPAT), using Clustal X 1.81, followed by shading with Boxshade 3.21. The gaps are indicated as dashes. **(B)** Phylogenetic tree of multiple GPAT proteins was constructed using the Neighbor-Joining method with the program MEGA 5.0. The NCBI accession numbers are as follows: SoGPAT (CAA88913), AtGPAT (AEE31448), and CmGPAT (BAB17755).
