# Supplementary material for: Quantitative Proteomic Profiling of Early and Late Responses to Salicylic Acid in Cucumber Leaves
Source: PLoS One. 2016 Aug 23;11(8):e0161395. doi: 10.1371/journal.pone.0161395 (PMC4995040; doi:10.1371/journal.pone.0161395)
Supplement: S6 Fig — The sequence alignment was performed using Clustal X 1.81 and colored by Boxshade 3.21. The gaps are indicated as dashes. Two conserved Gly residues (G158 and G422) are highlighted in red. (DOCX) [file pone.0161395.s006.docx]

**Supporting Information**


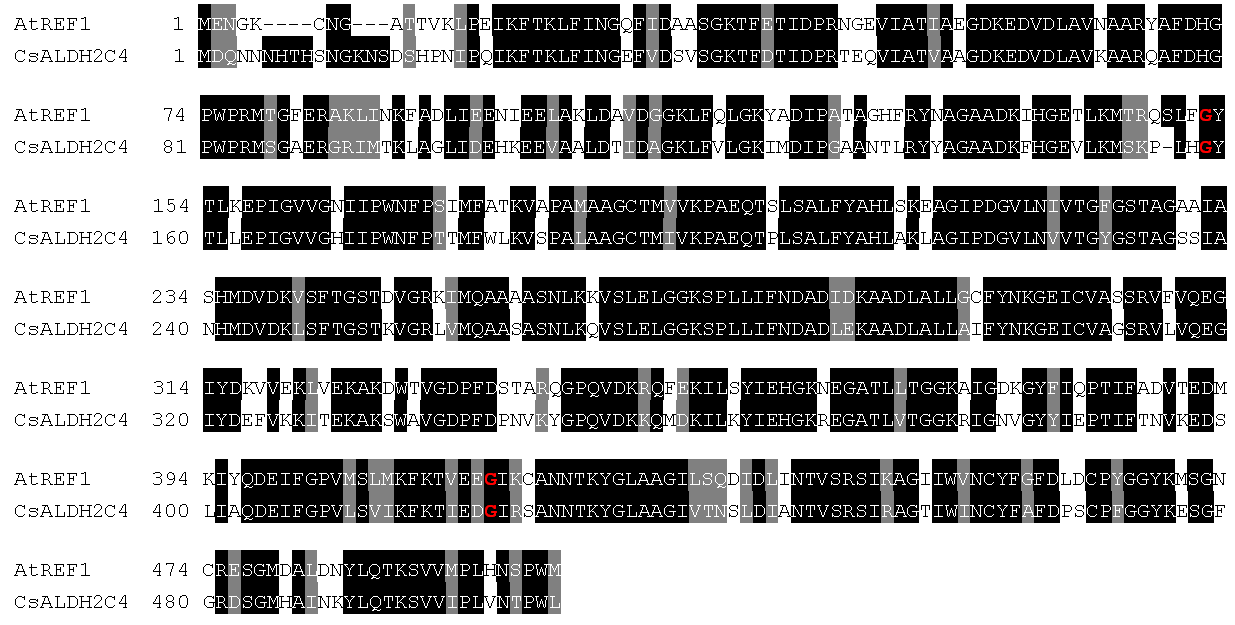


**S6 Fig. Cucumber ALDH2C4 shows high similarity with Arabidopsis REF1 protein.** The sequence alignment was performed using Clustal X 1.81 and colored by Boxshade 3.21. The gaps are indicated as dashes. Two conserved Gly residues (G158 and G422) are highlighted in red.
