# Supplementary material for: Quantitative Proteomic Profiling of Early and Late Responses to Salicylic Acid in Cucumber Leaves
Source: PLoS One. 2016 Aug 23;11(8):e0161395. doi: 10.1371/journal.pone.0161395 (PMC4995040; doi:10.1371/journal.pone.0161395)
Supplement: S7 Fig — Venn diagram analyses of the late SA-responsive DEPs from the present study and three other independent studies, including Hao et al. (59 DEPs, A), Kundu et al. (29 DEPs, B) and Kang et al. (62 DEPs, C). (DOCX) [file pone.0161395.s007.docx]

**Supporting Information**


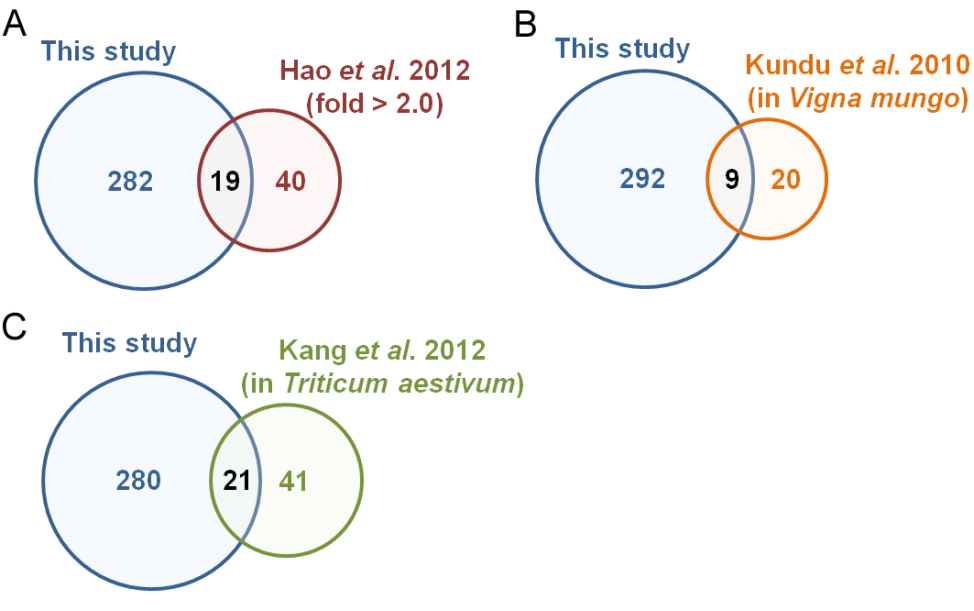


**S7 Fig. Venn diagram analyses of the late SA-responsive DEPs from the present study and three other independent studies, including Hao *et al.* (59 DEPs, A), Kundu *et al.* (29 DEPs, B) and Kang *et al.* (62 DEPs, C).**
