# Supplementary material for: Quantitative Proteomic Profiling of Early and Late Responses to Salicylic Acid in Cucumber Leaves
Source: PLoS One. 2016 Aug 23;11(8):e0161395. doi: 10.1371/journal.pone.0161395 (PMC4995040; doi:10.1371/journal.pone.0161395)
Supplement: S1 Table — (DOCX) [file pone.0161395.s008.docx]

**Supporting Information**

**S1 Table. Primer sequences used in the qRT-PCR.**

| **Gene** | **ICuGI Acc. No.** | **Primer Sequence** | |
| --- | --- | --- | --- |
| *PR1-1a* | DQ641122.1^*^ | PR1a-F  PR1a-R | 5’**-**AACTCTGGCGGACCTTAC-3’  5’**-**TCAATATGGCCTTTGGTATAAG-3’ |
| *GRXC9* | NM_001287209.1^*^ | GRXC9-F  GRXC9-R | 5’**-**GCAATCCCTTACCGATCATGGCA-3’  5’**-**TCGGAAACTGACGCAACGACGT-3’ |
| *ALDH* | Csa1M372010.1 | ALDH-F  ALDH-R | 5’**-**TGGCAGCGTTGGATACGATTGA-3’  5’**-**CTGACTTTGAGCCAGAACATGGT-3’ |
| *COMT* | Csa3M747630.1 | COMT-F  COMT-R | 5’**-**AAGCGGCGTACAGTTATTATGGGA-3’  5’**-**ACCTCCGGCAGATCGAAGTTAA-3’ |
| *CCOMT* | Csa7M073660.1 | CCOMT-F  CCOMT-R | 5’**-**GTGGATGCTGATAAAGACAACTACA-3’  5’**-**CAATCCTAGGGTCAGCAGCAA-3’ |
| *CHI2* | Csa4M622760.1 | CHI2-F  CHI2-R | 5’**-**ACAGATTGTTCTTGTCCGAGAC-3’  5’**-**GCATCTATTCCTGTTGGTGAGC-3’ |
| *CCR2* | Csa4M664500.1 | CCR2-F  CCR2-R | 5’**-**AGAACCGGCAACGGATGTTAGT-3’  5’**-**TCTCCTCCAATGGCCGATACT-3’ |
| *RFS* | Csa3M838720.1 | RFS-F  RFS-R | 5’**-**CATCACCAAAGAAGGAATGAACC-3’  5’**-**ATCCACACAAAGCATGCCAAACAT-3’ |
| *RFS2* | Csa1M002900.1 | RFS2-F  RFS2-R | 5’**-**TCAGGAGGAGCTATTGAAACTCT-3’  5’**-**GAACCATCCTCAAGTTTGACTGT-3’ |
| *SBE2.2* | Csa3M751970.1 | SBE2.2-F  SBE2.2-R | 5’**-**TAGAAGCTCATGAGAAGACAAAGGA-3’  5’**-**AAGATGTCCTCTGTGACTCAACA-3’ |
| *SPS2* | Csa2M121970.1 | SPS2-F  SPS2-R | 5’**-**CAACCTGTGTGGCCATATGTCA-3’  5’**-**TCACAAGCTCTGCAGCATCAAG-3’ |
| *DSP4* | Csa5M616890.1 | DSP4-F  DSP4-R | 5’**-**AGCTTCAAATGCCGACAGAGAAA-3’  5’**-**AGCACCCATAGCTTCTGTCAT-3’ |
| *DPE1* | Csa2M296010.1 | DPE1-F  DPE1-R | 5’**-**ATGGCGATCTCAACCTCACTATT-3’  5’**-**ATCCGATTCTGGAAACCATTCTC-3’ |
| *PEPC* | Csa4M627210.1 | PEPC-F  PEPC-R | 5’**-**TCGAAGAGCTTGGAAATGTGCTG-3’  5’**-**AGCTTCTTCAGAGTTTCCTCAATG-3’ |
| *PEPC4* | Csa5M577360.1 | PEPC4-F  PEPC4-R | 5’**-**TGGGAATTCACCTACTGGAAGTG-3’  5’**-**AATTCCAGGACGTTGAGGGAG-3’ |
| *FBP* | Csa4M307360.1 | FBP-F  FBP-R | 5’**-**ATGGTTCGTACGATTTCATCTTCG-3’  5’**-**TCTTCGATCGGATTTCCGGTTC-3’ |
| *TrxM4* | Csa3M104920.1 | TrxM4-F  TrxM4-R | 5’**-**ATGGCGACGGTTCTCGATTC-3’  5’**-**TGCAGAACGGTGAGGAAATCTAG-3’ |
| *TrxY1* | Csa6M014850.1 | TrxY1-F  TrxY1-R | 5’**-**ATGGCGGCCTCCTTTTCAAC-3’  5’**-**AAGGATGGAGAGGAGGATGGA-3’ |
| *SOD* | Csa4M025180.1 | SOD-F  SOD-R | 5’**-**ATCTGGAACCACGATTTTCTCTG-3’  5’**-**TGGATGGTCAACCGTGTTATC-3’ |
| *POD2* | Csa4M285760.1 | POD2-F  POD2-R | 5’**-**TACATTTGGACGATCGAGATGCA-3’  5’**-**TTCCCCACTTGAAGATTTGTGTAG-3’ |
| *LSMT* | Csa2M110250.1 | LSMT-F  LSMT-R | 5’**-**ATGGCCTACTCGAAACTTGAAACT-3’  5’**-**TGAAGGCAACGATGGATCTAAATC-3’ |
| *RA1* | Csa5M182730.1 | RA1-F  RA1-R | 5’**-**AAGTTCAGTCTTCTTTGGGAACAG-3’  5’**-**TCCTGTTGGTCATCTGATATATCG-3’ |
| *LUT1* | Csa6M448700.1 | LUT1  LUT1 | 5’**-**ATGGCTTCCTCTCTCTGCTTTC-3’  5’**-**AGGAACGGGATTTTGGAGCG-3’ |
| *CP12-2* | Csa4M003670.1 | CP12-2  CP12-2 | 5’**-**ATGGCTACCATTTCTGGGGTG-3’  5’**-**TGATATCTTATCCGGTGCAGCG-3’ |
| *α-GP* | Csa5M606600.1 | α-GP  α-GP | 5’**-**AGACTCGGTAATGGAACTGCTA-3’  5’**-**TCATCTTTGGCTGCTTTGGATCA-3’ |
| *NADP-ME* | Csa1M574870.1 | NADP-ME  NADP-ME | 5’**-**ATGGAGAGTACTTTGAAGGAGATC-3’  5’**-**TGTAAATGCCAACCCCTTGTTATG-3’ |
| *RPI* | Csa2M011530.1 | RPI  RPI | 5’**-**ACTCAAGACGACCTCAAGAAACT-3’  5’**-**TGACCTAGCTTGTTCTTCCGTT-3’ |
| *Fe-S* | Csa7M046100.1 | Fe-S  Fe-S | 5’**-**ATGTGCCAGCAGAAGTGCTTTG-3’  5’**-**TGTAAGGATACAACATAGTAGCAGTG-3’ |
| *Fd* | Csa3M146700.1 | Fd  Fd | 5’**-**TCCGGTCGTACAAAGTAGTGATTG-3’  5’**-**ACAACGTCATCGCTCAACATACC-3’ |
| *PC* | Csa3M875430.1 | PC  PC | 5’**-**CATGGCCATCGAGATCTTGCT-3’  5’**-**GTTCAAAAGATTCTCCTCATCCATC-3’ |
| *XK* | Csa6M404190.1 | XK  XK | 5’**-**TCGCTAATATTGCTGCAGTCTCT-3’  5’**-**TCCTCGATCTGCCGACATTGT-3’ |
| *PSaH* | Csa3M483830.1 | PsaH  PSaH | 5’**-**ACAGTGGGATTTGTATGGATCTGA-3’  5’**-**TGAGGTCCTTTCTTAATGGGAAG-3’ |
| *OEE1* | Csa6M488340.1 | OEE1  OEE1 | 5’**-**TACCCTTATGCAACCCTCCAAG-3’  5’**-**AAGAGCGGAAGTGGCAAGAG-3’ |
| *FTR* | Csa5M631540.1 | FTR  FTR | 5’**-**AGCTTCATCCTTCAATATCGCCG-3’  5’**-**ACAACAGCAGTAACCCCCTTGT-3’ |
| *TMP14-2* | Csa3M119840.1 | TMP14-2  TMP14-2 | 5’**-**TTCTCAACCCACTCGCCTCT-3’  5’**-**CTGTGAAGAGCTCAGATGCATC-3’ |
| *TMP14* | Csa7M447010.1 | TMP14  TMP14 | 5’**-**ACTTCCCTTGATATTGTTAAGTCTGTTC-3’  5’**-**CGATTTGGCTTGAACAGGAGGTA-3’ |
| *AGPS* | Csa7M030510.1 | AGPS  AGPS | 5’**-**TCGGCTCTATCCGCTCACTAA-3’  5’**-**TTGTAACCACCCATATTACTCGCA-3’ |
| *HK1* | Csa2M000830.2 | HK1  HK1 | 5’**-**ATAGTGGAGCTCTGTGACGTAG-3’  5’**-**ACCTCATCTCCAAGTAGCTCC-3’ |
| *COX5C* | Csa7M407690.1 | COX5C  COX5C | 5’**-**TCATGTCACACTACAAGGACCG-3’  5’**-**CTATTCTTCATCAACAACGACACTG-3’ |
| *chENO1* | Csa1M014400.1 | chENO1  chENO1 | 5’**-**AGTCGGAACTTGGGGCAAATG-3’  5’**-**TCTGCGAAAGATGAAGCACCTAC-3’ |
| *GPI1* | Csa2M372130.1 | GPI1-F  GPI1-R | 5’**-**TGGAGTAGATACGTTGACTGGC-3’  5’**-**TCAGAGTTCCTCAACCAATAATGC-3’ |
| *PU1* | Csa5M622510.1 | PU1-F  PU1-R | 5’**-**ATCAGCTTTCCCCACTTCCCTT-3’  5’**-**AAGCTCTCGAGTACAACAAGCTAT-3’ |
| *MDH* | Csa2M174150.1 | MDH-F  MDH-R | 5’**-**AATGGCGTAAAGATGGAGTTGGT-3’  5’**-**AGCCTGAGCCTTGTAAATTGAGA-3’ |
| *ICDH* | Csa6M446300.1 | ICDH-F  ICDH-R | 5’**-**AGATCCTCGAACTCAAAGTTTCCT-3’  5’**-**ATCGAGTTTTATAACCAGGTAGGCT-3’ |
| *OGDH* | Csa5M512860.1 | OGDH-F  OGDH-R | 5’**-**TAGCTTCTTAGATGGAACTAGCAG-3’  5’**-**TCCAACTTGGCCTTCATGTGAC-3’ |
| *GAPDH* | Csa1M050240.1 | GAPDH-F  GAPDH-R | 5’**-**AGATGATGTTGAACTCGTTGCTG-3’  5’**-**TGGACTCTACAATGTATTCCGCA-3’ |
| *GAE6* | Csa2M146400.1 | GAE6-F  GAE6-R | 5’**-**ATGTCCGTATTGGTCACCGGT-3’  5’**-**TTAGAGAGCAATGCGGCGTC-3’ |
| *PFPα* | Csa4M664520.1 | PFPα-F  PFPα-R | 5’**-**CTCTTGAGATCACCGATGACATTC-3’  5’**-**CAGCAAGTTGAGCAGCATCAGT-3’ |
| *ETFα* | Csa3M422290.1 | ETFα-F  ETFα-R | 5’**-**ATGCCAATTTCGTCTTGTGACTATG-3’  5’**-**AGAGATCAACCTGAGATATAGCG-3’ |
| *chPGM* | Csa3M893360.2 | chPGM-F  chPGM-R | 5’**-**GTTTGTATCCATCGCACCGTACT-3’  5’**-**CAATGGGGATGTGATGACTAACTC-3’ |
| *chPGM2* | Csa6M151120.1 | chPGM2-F  chPGM2-R | 5’**-**AGGTCGAGAAGTCTTCGGAATC-3’  5’**-**AGCCTCCCCTTTCTTAGTGAGA-3’ |
| *ATPδ* | Csa6M016970.1 | ATPδ-F  ATPδ-R | 5’**-**TCTTCAGGCCAAGCTCCTTC-3’  5’**-**CTTGTGGCGTCAAGTGTGTTG-3’ |
| *ATPα* | Csa5M321480.1 | ATPα-F  ATPα-R | 5’**-**CTGCGAGTGAGACATTGTATTGTG-3’  5’**-**ATGCGTGCATTCCATTATCGCG-3’ |
| *ACP1* | Csa6M067970.1 | ACP1-F  ACP1-R | 5’**-**ATGTTCTCCATCGCCGGATCT-3’  5’**-**CACCTTGTCCACTGTCTCTGG-3’ |
| *ACX* | Csa2M415540.1 | ACX-F  ACX-R | 5’**-**ATGGAGCGTGTTTCTTGGAGAAC-3’  5’**-**CACCATGAGCCCAAAAATCCAG-3’ |
| *AOS* | Csa7M075590.1 | AOS-F  AOS-R | 5’**-**AAGGGGAGAAGCTTTTGAAGTATGT-3’  5’**-**AGTGAACGTATCATAACGGAGGAA-3’ |
| *LOX2.1* | Csa4M288610.1 | LOX2.1-F  LOX2.1-R | TCACAAAACATCCTGTGTTGCTTC-3’  TGACCTTGTATTTAACCGGCTTC-3’ |
| *SQD2* | Csa3M732460.1 | SQD2-F  SQD2-R | ATGATGACCTCCACTTCTCTCTC-3’  5’**-**TACTCCTGAATGAACTTTTCAAGCAAG-3’ |
| *ACT* | Csa3M900970.1 | ACT-F  ACT-R | 5’**-**ATCATCAAACTGAAGCAGATCCAG-3’  5’**-**TCTGCAAGTTCAGGAATATCGAG-3’ |
| *FAB1* | Csa1M597750.1 | FAB1-F  FAB1-R | 5’**-**ATGGCTTCCTTACCCGTCTCTTC-3’  5’**-**TCCGTCTAGAGGTGGATATCGATC-3’ |
| *ENR* | Csa1M605670.1 | ENR-F  ENR-R | 5’**-**CTTTCACATATCAAGAACCCTATCCTAG-3’  5’**-**GCCCATTCTGTTGCGGTACAAC-3’ |
| *LCAT1* | Csa6M014730.1 | LCAT1-F  LCAT1-R | 5’**-**GTTTCAATGGCTTTGGTGCATCAG-3’  5’**-**CCTTCATGATTGGATTCCAACGG-3’ |
| *MFP* | Csa2M003610.1 | MFP-F  MFP-R | 5’**-**CTACCTGATGTATCTGTTGATATCGC-3’  5’**-**GGTAAACCAAGCTGAACTTTAGGTAC-3’ |
| *PI-PLC2* | Csa5M203390.1 | PI-PLC2-F  PI-PLC2-R | 5’**-**GCTTCTGCTTCCGCCGGCGT-3’  5’**-**CCTGGTGGTATTAGCCTCTTTCTG-3’ |
| *MGL* | Csa5M623380.1 | MGL-F  MGL-R | 5’**-**CGGTTGAAGACGCCGATGAG-3’  5’**-**ACGATGCCGATGATACCTATTGG-3’ |
| *ACBP* | Csa3M004510.1 | ACBP-F  ACBP-R | 5’**-**CTCATTCTCTATGGACTATACAAGCAG-3’  5’**-**GCTAGCAGCAGCTGCCTCT-3’ |
| *Actin* | Csa6M484600.1 | Actin-F  Actin-R | 5’**-**AGTATTGTTGGTCGTCCCCG-3’  5’**-**TCAGTGAGAAGAACTGGGTGTTC-3’ |

*, the gene sequences for PR1-1a and GRXC9 were obtained from NCBI database.
