# Supplementary material for: Quantitative Proteomic Profiling of Early and Late Responses to Salicylic Acid in Cucumber Leaves
Source: PLoS One. 2016 Aug 23;11(8):e0161395. doi: 10.1371/journal.pone.0161395 (PMC4995040; doi:10.1371/journal.pone.0161395)
Supplement: S3 Table — (DOCX) [file pone.0161395.s010.docx]

**Supporting Information**

**S3 Table. The 135 SA-responsive DEPs identified at 12 hpt in the iTRAQ assay and their functional classification.**

| **ICuGI Acc. No.** | **Abbreviation** | **Protein Description** | **Subcellular Loc.** | **Fold** | ***P*-Value** |
| --- | --- | --- | --- | --- | --- |
| **1. Photosynthesis (9)** | | | | | |
| **1.1 Light harvesting and electron transfer (9)** | | | | | |
| Csa6M133810.1 | NDHN | NAD(P)H-quinone oxidoreductase subunit N (ndhN)-like | Chloroplast | 1.561 | 9.658E-04 |
| Csa6M016970.1 | ATPδ | ATP synthase delta chain, chloroplastic-like | Chloroplast | 0.424 | 4.416E-05 |
| Csa3M119840.1 | TMP14 | Thylakoid membrane phosphoprotein 14 kDa, chloroplast precursor, putative | Chloroplast | 0.479 | 1.962E-03 |
| Csa3M146700.1 | Fd | Ferredoxin-2-like | Chloroplast | 2.419 | 1.612E-19 |
| Csa6M488340.1 | OEE1 | Oxygen-evolving enhancer protein 1, chloroplastic-like | Chloroplast | 1.517 | 1.518E-05 |
| Csa5M589390.1 |  | Chlorophyll a-b binding protein 7, chloroplastic-like | Chloroplast | 0.545 | 1.477E-06 |
| Csa1M009810.1 |  | Chlorophyll a-b binding protein P4, chloroplastic-like | Chloroplast | 0.600 | 4.627E-07 |
| **2. Carbohydrate metabolic process (7)** | | | | | |
| **2.1 Carbohydrate metabolism (1)** | | | | | |
| Csa2M296010.1 | DPE | 4-alpha-glucanotransferase, chloroplastic/amyloplastic; Amylomaltase; Disproportionating enzyme; D-enzyme; | Chloroplast | 3.355 | 2.624E-03 |
| **2.2 Glycolysis and TCA cycle (3)** | | | | | |
| Csa3M893360.2 | PGM | Phosphoglycerate mutase, putative | Chloroplast | 2.037 | 1.116E-04 |
| Csa1M014400.1 | chENO1 | Enolase, phosphopyruvate hydratase, putative; Enolase 1, chloroplastic-like | Chloroplast | 0.406 | 2.474E-07 |
| Csa5M512860.1 | OGDH | 2-oxoglutarate dehydrogenase, mitochondrial-like | Mitochondrion | 0.503 | 1.513E-04 |
| **2.3 Oxidative phosphorylation (1)** | | | | | |
| Csa3M422290.1 | ETFα | Electron transfer flavoprotein subunit alpha, mitochondrial-like | Mitochondrion | 0.334 | 8.878E-10 |
| **2.4 Pentose-phosphate shunt and Glucuronate pathway (2)** | | | | | |
| Csa2M011530.1 | RPI | Probable ribose-5-phosphate isomerase-like | Chloroplast | 0.454 | 2.681E-04 |
| Csa2M146400.1 | GAE6 | UDP-glucuronate 4-epimerase 6-like | Golgi apparatus | 1.741 | 5.606E-08 |
| **3. Lipid metablism (9)** | | | | | |
| Csa3M900970.1 | GPAT | Glycerol-3-phosphate acyltransferase (GPAT), chloroplastic precursor | Chloroplast | 0.397 | 2.230E-05 |
| Csa6M014730.1 | LCAT | Lecithin-cholesterol acyltransferase 1-like | Vacuole | 2.053 | 7.083E-05 |
| Csa5M623380.1 | MGL | Monoglyceride lipase-like protein |  | 2.325 | 1.426E-21 |
| Csa5M077190.1 | KAR | 3-oxoacyl-[acyl-carrier-protein] reductase (KAR)-like | Chloroplast | 1.774 | 2.496E-03 |
| Csa1M597750.1 | KARII | 3-oxoacyl-[acyl-carrier-protein] synthase II, chloroplastic-like | Chloroplast | 1.547 | 4.027E-10 |
| Csa1M077240.2 | TER | Probable trans-2-enoyl-CoA reductase, mitochondrial-like | Mitochondrion | 0.596 | 3.565E-03 |
| Csa7M075590.1 | AOS | Allene oxide synthase, chloroplastic-like | Chloroplast | 2.573 | 4.086E-03 |
| Csa6M490980.1 |  | PI-PLC X domain-containing protein At5g67130-like | Plasma membrane | 0.551 | 2.184E-03 |
| Csa3M843810.1 |  | Short-chain type dehydrogenase/reductase-like | Chloroplast | 0.546 | 5.429E-04 |
| **4. Transport (10)** | | | | | |
| **4.1 Transporter for proteins (7)** | | | | | |
| Csa2M318420.1 | IPO7 | Probable importin-7 homolog | Nucleus | 0.615 | 3.672E-03 |
| Csa3M126070.1 | BIG2 | Brefeldin A-inhibited guanine nucleotide-exchange protein 2-like | Golgi apparatus | 0.458 | 5.852E-03 |
| Csa3M913980.1 | RabD2c | Ras-related protein RABD2c-like | Golgi apparatus | 0.554 | 8.890E-05 |
| Csa4M082460.1 | Rab11D | Ras-related protein Rab11D-like protein | Golgi apparatus | 0.561 | 1.897E-06 |
| Csa3M812150.1 | SEC61 | Protein transport protein Sec61 subunit alpha-like | Endoplasmic reticulum | 0.399 | 6.001E-08 |
| Csa2M171830.1 | TOM22-2 | Mitochondrial import receptor subunit TOM22 homolog 2-like | Mitochondrion | 0.647 | 7.410E-04 |
| Csa4M017080.1 |  | Protein YIF1B-A-like | Golgi apparatus | 1.989 | 8.448E-07 |
| **4.2 Transporter for ions (3)** | | | | | |
| Csa4M006220.1 |  | ATPase 9, plasma membrane-type-like | Plasma membrane | 0.660 | 4.942E-03 |
| Csa2M009310.1 |  | ATPase 8, plasma membrane-type-like | Plasma membrane | 0.648 | 9.448E-05 |
| Csa1M404660.1 |  | Sodium/pyruvate cotransporter BASS2, chloroplastic-like | Chloroplast | 0.279 | 3.880E-07 |
| **5. Secondary metabolism (8)** | | | | | |
| **5.1 Phenylpropanoid pathway (1)** | | | | | |
| Csa5M158580.1 | AOC | Primary amine oxidase-like | Cytoplasm | 0.497 | 3.896E-06 |
| **5.2 Chlorophyll metabolism (2)** | | | | | |
| Csa4M311220.1 |  | Chlorophyll synthase, chloroplastic-like | Chloroplast | 1.746 | 2.041E-03 |
| Csa6M007980.1 |  | Protoporphyrinogen oxidase, chloroplastic-like | Chloroplast/Mitochondrion | 0.629 | 3.684E-03 |
| **5.3 Metabolism of plant hormones (3)** | | | | | |
| Csa3M135700.1 | ACO | 1-aminocyclopropane-1-carboxylate oxidase homolog 3-like | Cytoplasm | 0.291 | 2.086E-04 |
| Csa6M454350.2 |  | IAA-amino acid hydrolase ILR1-like |  | 0.353 | 1.266E-04 |
| Csa1M575150.1 |  | IAA-amino acid hydrolase ILR1-like 4-like |  | 0.494 | 2.047E-07 |
| **5.4 Metabolism of others (2)** | | | | | |
| Csa5M262270.1 |  | Thiamine biosynthetic bifunctional enzyme TH1, chloroplastic-like | Chloroplast | 0.665 | 4.579E-05 |
| Csa2M296070.1 |  | Geranyl diphosphate synthase (GPS), chloroplastic-like | Chloroplast | 0.427 | 1.729E-06 |
| **6. Cellular redox homeostasis (5)** | | | | | |
| Csa4M658590.1 | CAT | Catalase isozyme 1-like | Peroxisome | 2.023 | 2.558E-05 |
| Csa4M025180.1 | SOD | Superoxide dismutase [Fe], chloroplastic-like | Chloroplast | 2.891 | 1.973E-10 |
| Csa6M014850.1 | TrxY1 | Thioredoxin Y1, chloroplastic-like | Chloroplast | 0.533 | 4.516E-04 |
| Csa2M362500.1 | TrxH1 | Thioredoxin H1-like | Cytoplasm | 0.589 | 4.544E-03 |
| Csa6M526460.1 | TrxO1 | Thioredoxin O1 (Trxo1), mitochondrial-like | Mitochondrion | 0.466 | 8.561E-04 |
| **7. Proteolysis (10)** | | | | | |
| Csa2M099450.1 | TCP1-α | T-complex protein 1 subunit alpha-like | Cytoplasm | 1.667 | 3.593E-04 |
| Csa1M600870.1 | TCP1-η | Chaperonin containing T-complex protein 1 subunit eta-like | Cytoplasm | 1.660 | 1.225E-03 |
| Csa3M653410.1 |  | ATP-dependent Clp protease proteolytic subunit 2, mitochondrial-like | Mitochondrion | 0.477 | 1.117E-05 |
| Csa3M177380.1 |  | ATP-dependent Clp protease-related protein At4g12060, chloroplastic-like | Chloroplast | 0.623 | 2.656E-03 |
| Csa3M005040.1 |  | ATP-dependent Clp protease proteolytic subunit 4, chloroplastic-like | Chloroplast | 0.618 | 2.635E-02 |
| Csa6M077440.1 | RPN3 | Probable 26S proteasome non-ATPase regulatory subunit 3-like |  | 3.032 | 1.642E-06 |
| Csa5M636470.1 | Do8 | Protease Do-like 8, chloroplastic-like | Chloroplast | 1.721 | 3.729E-04 |
| Csa2M277660.1 |  | BTB/POZ domain-containing protein At5g67385-like |  | 2.252 | 2.293E-03 |
| Csa2M270180.1 | SBT1 | Subtilisin-like protease-like | Cell wall | 2.432 | 2.501E-07 |
| Csa3M178520.1 | SBT2 | Subtilisin-like protease-like | Cell wall | 2.039 | 8.296E-25 |
| **8. Protein folding (4)** | | | | | |
| Csa1M004910.1 |  | Prohibitin-1, mitochondrial-like isoform 1 | Mitochondrion | 0.529 | 7.456E-13 |
| Csa5M607990.2 |  | Chaperonin 60 subunit beta 4, chloroplastic-like | Chloroplast | 0.502 | 7.350E-03 |
| Csa3M168400.1 |  | Probable prefoldin subunit 3-like | Cytosol | 1.739 | 1.058E-06 |
| **9. Protein modification (2)** | | | | | |
| Csa2M401380.1 |  | Probably inactive leucine-rich repeat receptor-like protein kinase At2g25790-like | Plasma membrane | 1.525 | 6.302E-04 |
| Csa6M116680.1 |  | PHOT2 (PHOTOTROPIN 2)-like; FMN binding / blue light photoreceptor/ kinase/ protein serine/threonine kinase-like | Plasma membrane | 0.513 | 5.928E-03 |
| **10. Cell wall orgnization (2)** | | | | | |
| Csa3M150110.1 |  | Endo-1,3;1,4-beta-D-glucanase-like | Cell wall | 0.518 | 7.666E-03 |
| Csa6M366460.1 |  | Glucan endo-1,3-beta-glucosidase-like protein At1g69295-like | Cell wall | 1.570 | 5.596E-08 |
| **11. Response to stress (8)** | | | | | |
| Csa2M270210.1 | ERD7 | Early-responsive to dehydration 7 (ERD7)-like |  | 0.622 | 5.633E-03 |
| Csa3M733270.1 | USP | Universal stress protein family protein-like |  | 0.648 | 3.079E-04 |
| Csa4M296830.1 | AKR4C9 | Aldo-keto reductase family 4 member C9-like | Cytoplasm | 0.611 | 7.023E-07 |
| Csa2M234530.1 | CXE1 | Probable carboxylesterase 1 (CXE1) -like |  | 1.614 | 6.903E-09 |
| Csa5M148530.1 | LEA | Late embryogenesis abundant protein-like |  | 2.401 | 9.705E-04 |
| Csa6M404210.1 |  | Hypersensitive-induced response protein 1 | Vacuole | 1.513 | 3.800E-04 |
| Csa6M396650.1 |  | LysM domain-containing GPI-anchored protein 1-like | Plasma membrane | 0.468 | 6.517E-05 |
| **12. Transcription (10)** | | | | | |
| Csa4M092980.1 |  | Nipped-B-like protein, cohesin loading factor subunit SCC2 | Nucleus | 0.600 | 2.483E-06 |
| Csa1M418790.1 |  | Small nuclear ribonucleoprotein E-like isoform 1 | Nucleus, Spliceosome | 0.585 | 6.104E-04 |
| Csa7M397040.2 |  | Pre-mRNA-splicing factor SF2-like | Nucleus, Spliceosome | 0.552 | 6.303E-09 |
| Csa6M150550.1 |  | Histone deacetylase HDT1-like | Nucleus | 0.507 | 2.032E-04 |
| Csa4M001840.1 |  | U1 small nuclear ribonucleoprotein A-like | Nucleus | 0.484 | 4.819E-05 |
| Csa4M110070.1 |  | Splicing factor 3B subunit 1-like | Nucleus, Spliceosome | 4.949 | 1.563E-03 |
| Csa2M437070.1 |  | Transcription factor VIP1-like | Nucleus | 1.512 | 1.019E-07 |
| Csa6M426360.1 |  | BEL1-like homeodomain protein 1-like protein | Nucleus | 3.635 | 1.937E-06 |
| Csa1M408710.1 |  | Protein argonaute PNH1-like | Nucleus | 0.428 | 1.716E-04 |
| Csa7M407730.1 |  | INO80 complex subunit B isoform-like | Nucleus | 2.402 | 1.072E-14 |
| **13. Translation (16)** | | | | | |
| Csa5M589950.1 |  | 60S ribosomal protein L17-2-like | Cytosolic ribosome | 0.376 | 1.327E-04 |
| Csa3M739040.1 |  | 60S ribosomal protein L12-1-like | Cytosolic ribosome | 1.693 | 5.382E-04 |
| Csa7M397560.1 |  | 60S ribosomal protein L22-2-like | Cytosolic ribosome | 1.530 | 8.810E-03 |
| Csa2M193310.1 |  | 40S ribosomal protein S15-like | Cytosolic ribosome | 0.593 | 6.567E-06 |
| Csa4M166960.1 |  | 40S ribosomal protein S2-4-like | Cytosolic ribosome | 1.803 | 9.077E-03 |
| Csa1M042360.1 |  | 40S ribosomal protein S7-like | Cytosolic ribosome | 2.170 | 3.064E-12 |
| Csa3M271370.1 |  | 30S ribosomal protein 3, chloroplastic-like | Chloroplastic ribosome | 0.599 | 1.095E-03 |
| Csa3M837570.1 |  | 50S ribosomal protein L12, chloroplastic-like | Chloroplastic ribosome | 0.565 | 3.792E-03 |
| Csa5M524750.1 |  | 50S ribosomal protein L12, chloroplastic-like | Chloroplastic ribosome | 0.486 | 5.360E-04 |
| Csa5M174580.1 |  | Valine--tRNA ligase-like, Valyl-tRNA synthetase-like | Cytoplasm | 1.873 | 1.123E-05 |
| Csa7M033430.2 |  | Threonine--tRNA ligase-like | Cytoplasm | 0.591 | 5.124E-10 |
| Csa2M263980.1 |  | Translation initiation factor-like | Cytoplasm | 1.860 | 2.740E-05 |
| Csa1M537630.1 |  | Eukaryotic translation initiation factor 3 subunit A-like | Cytoplasm | 0.656 | 4.679E-05 |
| Csa1M597810.1 |  | Eukaryotic translation initiation factor 3 subunit K-like | Cytoplasm | 0.598 | 3.881E-03 |
| Csa3M728090.1 |  | Elongation factor P-like | Cytoplasm | 0.592 | 2.326E-03 |
| Csa3M134680.1 |  | U-box domain-containing protein 72-like | Nucleus | 1.519 | 2.748E-05 |
| **14. Nucleotide metabolism (4)** | | | | | |
| Csa1M574840.1 | GDA | Guanine deaminase-like | Cytosol? | 0.303 | 5.562E-04 |
| Csa3M110660.1 | PUR2 | Phosphoribosylamine--glycine ligase, chloroplastic-like | Chloroplast | 0.604 | 2.918E-25 |
| Csa1M386580.1 | NUDT20 | Nudix (nucleoside diphosphates linked to some moiety X) hydrolase 20, chloroplastic-like | Chloroplast | 0.262 | 1.586E-12 |
| Csa6M079760.1 | UCK1 | UMP/CMP kinase-like isoform 1 |  | 1.713 | 5.618E-08 |
| **15. Amino acid metabolism (4)** | | | | | |
| Csa4M563190.2 | ASI-2 | Anthranilate synthase component I-2, chloroplastic-like | Chloroplast | 2.446 | 2.180E-03 |
| Csa7M234130.1 | GLX1 | Lactoylglutathione lyase / glyoxalase I-like protein |  | 0.407 | 6.855E-05 |
| Csa7M234700.1 |  | Imidazoleglycerol-phosphate dehydratase (IGPD)-like | Chloroplast | 0.660 | 8.498E-04 |
| Csa4M507980.1 |  | Farnesylcysteine lyase-like | Lysosome | 1.862 | 1.126E-03 |
| **16. Structural molecules (1)** | | | | | |
| Csa4M026900.1 |  | Histone H3.3-like | Nucleus | 0.644 | 1.449E-18 |
| **17. Cell cycle (1)** | | | | | |
| Csa1M172630.1 |  | Sister chromatid cohesion protein PDS5 homolog B-like | Nucleus | 0.548 | 1.292E-04 |
| **18. Unclassified proteins (5)** | | | | | |
| Csa3M776950.1 |  | Heme-binding-like protein (similar to At3g10130), chloroplastic-like | Chloroplast | 0.658 | 0.01967 |
| Csa5M162620.1 |  | Inositol 2-dehydrogenase like protein |  | 1.830 | 1.924E-05 |
| Csa4M010990.1 |  | GEM-like protein 5-like |  | 1.918 | 4.646E-06 |
| Csa3M563290.1 |  | Major pollen allergen-like |  | 1.871 | 3.683E-03 |
| Csa4M617330.1 |  | Neuferricin-like, Cytochrome b5 domain-containing protein 2-like |  | 0.496 | 5.066E-03 |
| **19. Uncharacterized proteins (20)** | | | | | |
| Csa6M151650.1 |  | Hypothetical protein |  | 0.197 | 1.315E-03 |
| Csa6M151660.1 |  | Uncharacterized protein LOC101208002 isoform 2 |  | 0.287 | 5.954E-05 |
| Csa6M046350.1 |  | Uncharacterized protein At2g39795, mitochondrial-like | Mitochondrion | 0.291 | 5.600E-06 |
| Csa7M069190.1 |  | Uncharacterized protein LOC101210086 |  | 0.343 | 1.905E-19 |
| Csa3M159420.1 |  | Uncharacterized protein LOC101205403 |  | 0.428 | 1.544E-05 |
| Csa1M665900.1 |  | Uncharacterized protein LOC101217511 |  | 0.444 | 8.685E-03 |
| Csa3M638530.1 |  | Uncharacterized protein LOC101212883 |  | 0.495 | 7.589E-07 |
| Csa5M638300.1 |  | Uncharacterized protein LOC101202842 |  | 0.584 | 1.962E-03 |
| Csa3M686720.1 |  | Uncharacterized protein LOC101221174 |  | 0.597 | 1.111E-05 |
| Csa3M002600.1 |  | Uncharacterized protein LOC101219661 |  | 0.605 | 2.474E-29 |
| Csa7M447880.1 |  | Uncharacterized protein LOC101213828 |  | 0.641 | 1.246E-05 |
| Csa2M297210.2 |  | Uncharacterized mitochondrial carrier C12B10.09-like | Mitochondrion | 0.657 | 1.889E-05 |
| Csa4M110050.1 |  | Uncharacterized protein LOC101213735 |  | 0.657 | 0.02559 |
| Csa3M878190.1 |  | Uncharacterized protein LOC101207396 |  | 1.534 | 4.629E-03 |
| Csa3M047750.1 |  | Hypothetical protein |  | 1.543 | 4.422E-03 |
| Csa2M072460.1 |  | Uncharacterized protein LOC101212517 |  | 1.755 | 2.772E-07 |
| Csa6M486890.1 |  | Uncharacterized protein LOC101206379 |  | 1.781 | 1.546E-03 |
| Csa6M103010.1 |  | Uncharacterized protein LOC101213029 |  | 1.898 | 2.030E-19 |
| Csa7M013950.1 |  | Uncharacterized protein LOC101228360 |  | 2.329 | 9.386E-04 |
| Csa5M505130.1 |  | Uncharacterized protein LOC101221723 |  | 3.513 | 3.251E-03 |
