# Supplementary material for: Quantitative Proteomic Profiling of Early and Late Responses to Salicylic Acid in Cucumber Leaves
Source: PLoS One. 2016 Aug 23;11(8):e0161395. doi: 10.1371/journal.pone.0161395 (PMC4995040; doi:10.1371/journal.pone.0161395)
Supplement: S4 Table — (DOCX) [file pone.0161395.s011.docx]

**Supporting Information**

**S3 Table. The 301 SA-responsive DEPs identified at 72 hpt in the iTRAQ assay and their functional classification.**

| **ICuGI Acc. No.** | **Abbreviation** | **Protein Description** | **Subcellular Loc.** | **Fold** | ***P*-Value** |
| --- | --- | --- | --- | --- | --- |
| **1. Photosynthesis (22)** | | | | | |
| **1.1 Light harvesting and electron transfer (17)** | | | | | |
| Csa6M133810.1 | NDHN | NAD(P)H-quinone oxidoreductase subunit N (ndhN)-like | Chloroplast | 1.552 | 2.244E-13 |
| Csa3M119840.1 | TMP14 | Thylakoid membrane phosphoprotein 14 kDa, chloroplast precursor, putative | Chloroplast | 1.596 | 7.324E-03 |
| Csa5M589930.1 | PsbB | Photosystem II 47 kDa protein | Chloroplast | 1.736 | 1.117E-06 |
| Csa3M119660.1 | PsbQ | PsbQ-like protein 1, chloroplastic-like | Chloroplast | 1.822 | 5.755E-19 |
| Csa1M009810.1 |  | Chlorophyll a-b binding protein P4, chloroplastic-like | Chloroplast | 1.572 | 0.02808 |
| Csa7M033300.1 | CAB151 | Chlorophyll a-b binding protein 151, chloroplastic-like | Chloroplast | 1.584 | 3.003E-03 |
| Csa3M099680.1 | CAB13 | Chlorophyll a-b binding protein 13, chloroplastic-like | Chloroplast | 1.562 | 8.393E-03 |
| Csa6M139750.1 |  | Chlorophyll a-b binding protein, chloroplastic-like | Chloroplast | 1.557 | 6.380E-05 |
| Csa6M057170.1 |  | Chlorophyll a-b binding protein CP24 10A, chloroplastic-like | Chloroplast | 1.550 | 1.068E-24 |
| Csa2M074240.1 | PsaL | Photosystem I reaction center subunit XI, chloroplastic | Chloroplast | 1.557 | 0.00633 |
| Csa7M447010.1 | TMP14-2 | Thylakoid membrane phosphoprotein 14 kDa, chloroplastic-like isoform 2 | Chloroplast | 1.935 | 7.756E-07 |
| Csa4M063440.1 | OEE2 | Oxygen-evolving enhancer protein 2 (OEE2), 23 kDa subunit of oxygen evolving system of photosystem II (OEC23) | Chloroplast | 1.512 | 2.255E-16 |
| Csa1M066480.1 | OEE3-2 | Oxygen-evolving enhancer protein 3-2, chloroplastic-like | Chloroplast | 0.661 | 1.497E-03 |
| Csa6M488340.1 | OEE1 | Oxygen-evolving enhancer protein 1, chloroplastic-like | Chloroplast | 1.552 | 0.00889 |
| Csa7M046100.1 | Fe-S | Cytochrome b6-f complex iron-sulfur subunit, chloroplastic-like | Chloroplast | 1.586 | 5.290E-03 |
| Csa3M483830.1 | PsaH | Photosystem I reaction center subunit VI, chloroplastic-like | Chloroplast | 1.587 | 4.113E-07 |
| Csa5M631540.1 | FTR | Ferredoxin-thioredoxin reductase catalytic chain, chloroplastic-like | Chloroplast | 1.613 | 0.04410 |
| **1.2 CO_2_ fixation (5)** | | | | | |
| Csa2M110250.1 | LSMT | Ribulose-1,5 bisphosphate carboxylase/oxygenase large subunit N-methyltransferase, chloroplastic-like | Chloroplast | 2.061 | 6.756E-05 |
| Csa5M182730.1 | RA1 | Ribulose bisphosphate carboxylase/oxygenase activase 1 (RA 1), chloroplastic-like | Chloroplast | 2.004 | 0.00366 |
| Csa5M609710.1 | RbcS | Ribulose bisphosphate carboxylase small chain, chloroplastic | Chloroplast | 1.871 | 0.04010 |
| Csa4M003670.1 | CP12-2 | Calvin cycle protein CP12-2, chloroplastic-like | Chloroplast | 1.846 | 0.02134 |
| Csa4M307360.1 | FBP | Fructose-1,6-bisphosphatase, chloroplastic-like | Chloroplast | 1.724 | 4.264E-14 |
| **2. Carbohydrate metabolic process (27)** | | | | | |
| **2.1 Carbohydrate metabolism (10)** | | | | | |
| Csa3M838720.1 | RFS | Raffinose synthase | Cytoplasm | 2.670 | 0.03395 |
| Csa1M002900.1 | RFS2 | Probable galactinol--sucrose galactosyltransferase 2-like, raffinose synthase-like | Cytoplasm | 2.687 | 8.295E-03 |
| Csa2M121970.1 | SPS2 | Sucrose-phosphate synthase 2-like | Cytoplasm | 3.212 | 0.02484 |
| Csa3M751970.1 | SBE2.2 | 1,4-alpha-glucan-branching enzyme 2-2, chloroplastic/amyloplastic-like | Chloroplast | 2.011 | 4.776E-04 |
| Csa2M296010.1 | DPE | 4-alpha-glucanotransferase, chloroplastic/amyloplastic; Amylomaltase; Disproportionating enzyme; D-enzyme; | Chloroplast | 2.689 | 1.566E-12 |
| Csa5M622510.1 | PU1 | Pullulanase 1, chloroplastic-like | Chloroplast | 2.505 | 2.487E-05 |
| Csa7M030510.1 | AGPS | Glucose-1-phosphate adenylyltransferase small subunit, chloroplastic-like | Chloroplast | 1.505 | 0.01774 |
| Csa5M606600.1 | GP | Alpha-1,4 glucan phosphorylase L-2 isozyme, chloroplastic/amyloplastic-like | Chloroplast | 0.617 | 2.584E-05 |
| Csa5M616890.1 | DSP4 | Phosphoglucan phosphatase, Dual specificity protein phosphatase 4 (DSP4), chloroplastic-like | Chloroplast | 1.675 | 0.03757 |
| Csa7M007830.1 | S6PDH | NADP-dependent D-sorbitol-6-phosphate dehydrogenase-like | Cytoplasm | 2.717 | 0.00782 |
| **2.2 Glycolysis and TCA cycle (11)** | | | | | |
| Csa6M151120.1 | chPGM | Putative phosphoglycerate mutase | Chloroplast | 1.862 | 1.035E-07 |
| Csa1M014400.1 | chENO1 | Enolase, phosphopyruvate hydratase, putative; Enolase 1, chloroplastic-like | Chloroplast | 0.420 | 6.273E-07 |
| Csa2M372130.1 | chGPI1 | Glucose-6-phosphate isomerase (GPI) 1, chloroplastic-like | Chloroplast | 1.749 | 8.395E-21 |
| Csa4M664520.1 | PFP-α | Pyrophosphate--fructose 6-phosphate 1-phosphotransferase subunit alpha-like | Cytoplasm | 2.284 | 4.947E-03 |
| Csa2M000830.2 | HK1 | Hexokinase-1-like | Cytoplasm | 2.112 | 2.909E-03 |
| Csa1M050240.1 | GAPDH | Glyceraldehyde-3-phosphate dehydrogenase | Cytoplasm | 2.064 | 1.905E-06 |
| Csa4M627210.1 | PEPC | Phosphoenolpyruvate carboxylase, housekeeping isozyme-like | Cytoplasm | 2.016 | 4.187E-04 |
| Csa5M577360.1 | PEPC4 | Phosphoenolpyruvate carboxylase 4-like | Cytoplasm | 2.504 | 0.02836 |
| Csa1M574870.1 | NME | NADP-dependent malic enzyme-like | Cytoplasm | 2.086 | 3.665E-04 |
| Csa2M174150.1 | MDH | Malate dehydrogenase, cytoplasmic-like | Cytoplasm | 0.491 | 0.02408 |
| Csa6M446300.1 | ICDH | Isocitrate dehydrogenase [NAD] catalytic subunit 5, mitochondrial-like | Mitochondrion | 0.555 | 2.439E-09 |
| **2.3 Oxidative phosphorylation (4)** | | | | | |
| Csa5M166990.1 | QOR | Quinone oxidoreductase, NADPH2:quinone reductase | Mitochondrion | 0.550 | 2.293E-05 |
| Csa7M407690.1 | COX5C | Cytochrome c oxidase subunit 5C-like isoform 1 | Mitochondrion | 1.682 | 2.108E-08 |
| Csa6M295960.1 | ATPα | ATP synthase subunit alpha | Mitochondrion | 2.271 | 0.02141 |
| Csa5M321480.1 | ATPase-α2 | ATP synthase subunit alpha 2, mitochondrial | Mitochondrion | 0.349 | 1.857E-05 |
| **2.4 Pentose-phosphate shunt and Glucuronate pathway (2)** | | | | | |
| Csa6M404190.1 | XK | Xylulose kinase-like | Cytoplasm | 1.717 | 2.777E-28 |
| Csa2M146400.1 | GAE6 | UDP-glucuronate 4-epimerase 6-like | Golgi apparatus | 2.085 | 1.745E-17 |
| **3. Lipid metablism (16)** | | | | | |
| Csa6M014730.1 | LCAT | Lecithin-cholesterol acyltransferase 1-like | Vacuole | 0.338 | 7.687E-03 |
| Csa5M623380.1 | MGL | Monoglyceride lipase-like protein |  | 0.413 | 5.198E-13 |
| Csa3M004510.1 | ACBP1 | 10-kDa acyl-CoA-binding protein-like isoform 1 | Chloroplast | 2.660 | 3.039E-03 |
| Csa3M732460.1 | SQD2 | GDP-mannose-dependent alpha-mannosyltransferase-like (sulfoquinovosyltransferase, SQD2) | Chloroplast | 0.378 | 0.02148 |
| Csa3M843810.1 |  | Short-chain type dehydrogenase/reductase-like | Chloroplast | 0.555 | 9.018E-03 |
| Csa2M350440.1 | KAR | 3-oxoacyl-[acyl-carrier-protein] reductase, chloroplastic-like | Chloroplast | 0.630 | 0.01175 |
| Csa1M605670.1 | ENR | Enoyl-[acyl-carrier-protein] reductase [NADH], chloroplastic-like | Chloroplast | 2.204 | 3.973E-03 |
| Csa1M597750.1 | KARII | 3-oxoacyl-[acyl-carrier-protein] synthase II, chloroplastic-like | Chloroplast | 2.453 | 1.443E-09 |
| Csa6M067970.1 | ACP1 | Acyl carrier protein 1 (ACP1), chloroplastic precursor-like | Chloroplast | 2.887 | 0.01916 |
| Csa4M288610.1 | LOX2.1 | Linoleate 13S-lipoxygenase 2-1, chloroplastic-like | Chloroplast | 2.064 | 8.930E-11 |
| Csa7M075590.1 | AOS | Allene oxide synthase, chloroplastic-like | Chloroplast | 0.414 | 3.682E-10 |
| Csa3M128880.1 | TGDG4 | Protein TRIGALACTOSYLDIACYLGLYCEROL 4, chloroplastic-like | Chloroplast | 1.539 | 4.145E-22 |
| Csa2M415540.1 | ACX | Acyl-coenzyme A oxidase 3, peroxisomal-like | Peroxisome | 2.037 | 8.359E-04 |
| Csa2M003610.1 | MTF | Peroxisomal fatty acid beta-oxidation multifunctional protein AIM1-like | Peroxisome | 2.087 | 7.549E-05 |
| Csa1M050100.1 | GDSL | GDSL esterase/lipase At3g48460-like | Extracellular region | 3.058 | 1.791E-05 |
| Csa5M203390.1 | PI-PLC2 | Phosphoinositide phospholipase C 2-like | Plasma membrane | 1.881 | 0.02084 |
| **4. Transport (33)** | | | | | |
| **4.1 Transporter for proteins (25)** | | | | | |
| Csa6M312550.1 |  | Importin subunit alpha-1-like, Karyopherin subunit alpha-1-like | Nucleus | 2.000 | 5.729E-10 |
| Csa2M034640.1 |  | Importin subunit alpha-1-like, Karyopherin subunit alpha-2-like | Nucleus | 1.838 | 7.386E-07 |
| Csa3M119490.1 |  | Exportin-2-like | Nucleus | 1.779 | 0.01982 |
| Csa4M012400.1 |  | Nuclear transport factor 2-like | Nucleus | 0.641 | 0.03617 |
| Csa3M516570.1 |  | Coatomer subunit beta'-2-like | Golgi apparatus | 1.774 | 2.574E-03 |
| Csa2M169730.1 |  | Protein transport protein Sec24-like At4g32640-like | Golgi apparatus | 0.567 | 4.272E-04 |
| Csa3M126070.1 |  | Brefeldin A-inhibited guanine nucleotide-exchange protein 2-like | Golgi apparatus | 0.462 | 3.345E-09 |
| Csa3M734360.1 |  | Probable ADP-ribosylation factor GTPase-activating protein AGD6-like | Golgi apparatus | 1.950 | 2.609E-06 |
| Csa3M910690.1 |  | Ras-related protein RABH1b-like | Golgi apparatus | 1.562 | 2.207E-08 |
| Csa1M181560.1 |  | Vesicle-fusing ATPase-like | Cytoplasmic vesicle | 0.415 | 1.296E-04 |
| Csa2M352400.1 |  | Clathrin interactor EPSIN 1-like | Cytoplasmic vesicle | 0.572 | 3.444E-06 |
| Csa1M533450.1 |  | Exocyst complex component EXO70B1-like | Cytoplasmic vesicle | 2.205 | 4.390E-03 |
| Csa3M462670.1 |  | Protein SEC13 homolog | Endoplasmic reticulum | 3.828 | 9.414E-03 |
| Csa3M812150.1 |  | Protein transport protein Sec61 subunit alpha-like | Endoplasmic reticulum | 2.469 | 3.008E-08 |
| Csa6M404150.1 |  | Mitochondrial outer membrane protein porin of 36 kDa-like | Mitochondrion | 0.660 | 0.04171 |
| Csa5M146860.1 |  | Mitochondrial import inner membrane translocase subunit Tim13-like | Mitochondrion | 0.639 | 4.722E-04 |
| Csa2M351590.1 |  | Mitochondrial import receptor subunit TOM20-like | Mitochondrion | 0.662 | 2.909E-10 |
| Csa2M171830.1 |  | Mitochondrial import receptor subunit TOM22 homolog 2-like | Mitochondrion | 0.635 | 0.01479 |
| Csa3M002790.1 |  | Translocase of chloroplast 34, chloroplastic-like | Chloroplast | 1.711 | 9.115E-12 |
| Csa1M573670.1 |  | Sec-independent protein translocase protein TatA, chloroplastic-like | Chloroplast | 0.653 | 0.03028 |
| Csa4M651770.1 |  | Protein TIC 55, chloroplastic-like | Chloroplast | 1.635 | 1.401E-04 |
| Csa4M310190.1 |  | LIMR family protein At3g08930-like | Plasma membrane | 0.477 | 8.836E-03 |
| Csa3M819890.1 |  | Pentatricopeptide repeat-containing protein At1g80270, mitochondrial-like | Mitochondrion | 2.013 | 5.046E-05 |
| Csa3M002900.1 |  | Tetratricopeptide repeat domain-containing protein-like |  | 1.521 | 7.077E-15 |
| Csa1M266160.1 |  | Transmembrane 9 superfamily member 4-like |  | 1.824 | 0.02478 |
| **4.2 Transporter for H_2_O (2)** | | | | | |
| Csa5M153020.1 |  | Probable aquaporin PIP1-2-like | Plasma membrane | 1.753 | 3.631E-13 |
| Csa5M198770.1 |  | Probable aquaporin PIP1-4-like | Plasma membrane | 1.766 | 4.131E-08 |
| **4.3 Transporter for ions (6)** | | | | | |
| Csa5M457760.1 |  | Phosphate carrier protein, mitochondrial-like | Mitochondrion | 1.532 | 1.453E-08 |
| Csa2M370360.1 |  | Protein CutA, chloroplastic-like | Chloroplast | 1.611 | 2.809E-08 |
| Csa5M615220.1 |  | Copper transport protein ATOX1-like isoform 3 | Cytoplasm | 0.664 | 5.742E-04 |
| Csa2M301480.1 |  | Auxin-induced protein 5NG4-like | Plasma membrane | 0.404 | 8.093E-28 |
| Csa4M146240.1 |  | Putative lipid transfer protein family protein |  | 0.507 | 6.298E-06 |
| Csa7M061200.1 |  | Non-specific lipid-transfer protein-like protein At5g64080-like | Plasma membrane | 0.588 | 2.878E-03 |
| **5. Secondary metabolism (16)** | | | | | |
| **5.1 Phenylpropanoid pathway (7)** | | | | | |
| Csa4M664500.1 | CCR2 | Cinnamoyl-CoA reductase 2-like | Cytoplasm | 2.069 | 5.292E-05 |
| Csa3M747630.1 | COMT | Caffeic acid 3-O-methyltransferase-like | Cytoplasm | 2.293 | 1.950E-04 |
| Csa7M073660.1 | CCOMT | Caffeoyl-CoA O-methyltransferase 1-like | Cytoplasm | 2.090 | 1.826E-03 |
| Csa1M372010.1 | ALDH2C4 | Aldehyde dehydrogenase family 2 member C4-like | Cytoplasm | 2.432 | 2.420E-03 |
| Csa4M622760.1 | CHI2 | Chalcone--flavonone isomerase 2-like | Cytoplasm | 2.118 | 2.575E-08 |
| Csa5M167150.1 | PCD | Putative pterin-4-alpha-carbinolamine dehydratase-like | Cytoplasm | 0.630 | 4.410E-08 |
| Csa6M014690.1 | HMSH | 2-hydroxymuconate semialdehyde hydrolase-like | Cytoplasm | 6.524 | 4.006E-05 |
| **5.2 Chlorophyll metabolism (3)** | | | | | |
| Csa6M448700.1 | LUT1 | Carotene epsilon-monooxygenase, chloroplastic-like | Chloroplast | 2.439 | 3.112E-03 |
| Csa6M512870.1 | CHRC | Chromoplast-specific carotenoid-associated protein, chromoplastic precursor | Chloroplast | 3.077 | 2.210E-05 |
| Csa2M285300.1 | RCCR | Red chlorophyll catabolite reductase, chloroplastic-like | Chloroplast | 1.548 | 4.065E-04 |
| **5.3 Metabolism of plant hormones (4)** | | | | | |
| Csa7M058650.1 |  | UDP-glycosyltransferase 85A7-like |  | 1.537 | 3.342E-14 |
| Csa4M017150.1 |  | UDP-glycosyltransferase 92A1-like |  | 1.586 | 0.01675 |
| Csa6M454350.2 |  | IAA-amino acid hydrolase ILR1-like |  | 0.359 | 7.286E-11 |
| Csa1M575150.1 |  | IAA-amino acid hydrolase ILR1-like 4-like |  | 0.546 | 7.429E-03 |
| **5.4 Metabolism of others (2)** | | | | | |
| Csa2M437130.1 |  | S-norcoclaurine synthase-like isoform 1 |  | 1.953 | 7.211E-03 |
| Csa4M049620.1 |  | 2-C-methyl-D-erythritol 2,4-cyclodiphosphate synthase, chloroplastic-like | Chloroplast | 0.629 | 0.01306 |
| **6. Cellular redox homeostasis (7)** | | | | | |
| Csa4M285760.1 | POD2 | Peroxidase 2 | Chloroplast | 2.020 | 2.188E-04 |
| Csa4M025180.1 | SOD | Superoxide dismutase [Fe], chloroplastic-like | Chloroplast | 2.009 | 0.01544 |
| Csa3M104920.1 | TrxM4 | Thioredoxin M4, chloroplastic-like | Chloroplast | 1.680 | 3.199E-17 |
| Csa6M014850.1 | TrxY1 | Thioredoxin Y1, chloroplastic-like | Chloroplast | 1.680 | 0.02741 |
| Csa3M133380.1 | GST | Probable glutathione S-transferase-like | Plasma membrane | 3.578 | 5.531E-12 |
| Csa4M064630.1 | GST-U19 | Glutathione S-transferase U19-like | Plasma membrane | 1.520 | 1.447E-10 |
| Csa3M731110.1 | SelT | SelT (selenoprotein T)-like protein | Plasma membrane | 2.049 | 3.881E-32 |
| **7. Proteolysis (12)** | | | | | |
| Csa5M644550.1 |  | T-complex protein 1 subunit zeta-like | Cytoplasm | 1.782 | 0.01197 |
| Csa2M099450.1 |  | T-complex protein 1 subunit alpha-like | Cytoplasm | 1.534 | 0.04967 |
| Csa6M290910.1 |  | Proteasome subunit beta type-4-like | Cytoplasm | 1.578 | 6.397E-03 |
| Csa5M579570.1 |  | Protease 2-like | Mitochondrion | 1.513 | 8.002E-06 |
| Csa7M453520.1 |  | ATP-dependent zinc metalloprotease FtsH 3-like | Chloroplast | 0.401 | 1.619E-05 |
| Csa3M017170.1 |  | ATP-dependent Clp protease proteolytic subunit-related protein 4, chloroplastic-like | Chloroplast | 1.573 | 2.227E-25 |
| Csa1M528580.1 |  | ATP-dependent zinc metalloprotease FTSH 9, chloroplastic-like | Chloroplast | 1.523 | 1.719E-03 |
| Csa6M124220.1 |  | Metacaspase-5-like | Chloroplast | 0.652 | 1.029E-05 |
| Csa6M493920.1 |  | Subtilisin-like protease | Cell wall | 0.664 | 6.810E-03 |
| Csa3M178520.1 |  | Subtilisin-like protease-like | Cell wall | 3.801 | 1.717E-04 |
| Csa7M291690.1 |  | Xylem serine proteinase 1-like isoform 1 | Cell wall | 0.662 | 3.254E-10 |
| Csa7M276180.1 |  | Xylem serine proteinase 1-like isoform 1 | Cell wall | 0.607 | 5.124E-04 |
| **8. Protein folding (5)** | | | | | |
| Csa1M597740.1 |  | 20 kDa chaperonin, chloroplastic-like isoform 2 | Chloroplast | 0.624 | 4.117E-04 |
| Csa5M638310.1 |  | Probable prefoldin subunit 2-like | Cytosol | 0.309 | 1.296E-11 |
| Csa2M010310.1 |  | Probable prefoldin subunit 4-like | Cytosol | 0.642 | 6.460E-07 |
| Csa4M417510.1 |  | Protein GrpE (HSP-70 cofactor)-like | Chloroplast | 1.603 | 4.370E-03 |
| Csa2M009390.1 |  | Large proline-rich protein BAG6-like |  | 0.639 | 2.445E-03 |
| **9. Protein modification (7)** | | | | | |
| Csa6M525410.1 |  | Serine/threonine-protein phosphatase 6 regulatory subunit 3-like | Nucleus | 1.836 | 0.01444 |
| Csa1M181440.1 |  | Dolichyl-diphosphooligosaccharide--protein glycosyltransferase subunit 2-like | Endoplasmic reticulum | 1.602 | 8.920E-06 |
| Csa6M141380.1 |  | Serine/threonine-protein phosphatase 6 regulatory subunit 3-like | Plasma membrane | 0.649 | 1.294E-07 |
| Csa7M451930.3 |  | Serine/threonine protein phosphatase 2A 55 kDa regulatory subunit B beta isoform-like |  | 1.685 | 6.565E-03 |
| Csa6M358660.1 |  | Probable serine/threonine-protein kinase At4g35230-like | Chloroplast | 1.612 | 0.02816 |
| Csa2M428410.1 |  | SUMO-conjugating enzyme SCE1-like |  | 0.339 | 4.337E-05 |
| Csa1M568550.1 |  | Probable protein phosphatase 2C 5-like | Plasma membrane | 0.540 | 3.282E-07 |
| **10. Cell wall orgnization (6)** | | | | | |
| Csa7M019900.1 | EXLA1 | Expansin-like A1-like | Cell wall | 0.612 | 0.04565 |
| Csa6M309980.1 | BXL1 | Beta-D-xylosidase 1-like | Cell wall | 0.655 | 7.984E-04 |
| Csa2M348790.1 | PG | Polygalacturonase At1g48100-like | Cell wall | 0.600 | 0.01737 |
| Csa6M366530.1 | UPTG | Alpha-1,4-glucan-protein synthase [UDP-forming]-like | Cell wall | 1.841 | 0.01132 |
| Csa1M611290.1 | βGLU24 | Beta-glucosidase 24-like | Cell wall | 2.425 | 9.477E-04 |
| Csa1M605100.1 | CALS9 | Callose synthase 9-like | Plasma membrane | 1.857 | 0.04119 |
| **11. Response to stress (16)** | | | | | |
| Csa6M358710.1 | COR47 | Dehydrin COR47-like isoform 1 |  | 1.524 | 2.337E-05 |
| Csa5M524760.1 | USPA | Universal stress protein A-like protein |  | 2.017 | 1.291E-11 |
| Csa6M115630.1 |  | Ankyrin repeat domain-containing protein 2-like |  | 0.662 | 0.02091 |
| Csa7M010300.1 |  | Glycine-rich protein 2-like | Nucleus | 0.664 | 2.861E-04 |
| Csa1M031740.1 |  | Aldo-keto reductase 2 subfamily protein, protein tas-like | Cytoplasm | 1.508 | 1.420E-06 |
| Csa4M354630.1 | P5CR | Pyrroline-5-carboxylate reductase-like | Cytoplasm | 2.261 | 0.01892 |
| Csa2M249900.1 | RD19a | Cysteine proteinase RD19a-like |  | 0.601 | 1.369E-07 |
| Csa7M398090.1 |  | Indole-3-acetic acid-induced protein ARG2-like (LEA protein) |  | 0.546 | 1.356E-06 |
| Csa7M450510.1 |  | Auxin-binding protein ABP19a-like | Apoplast | 0.488 | 0.01008 |
| Csa6M084580.1 |  | Disease resistance response protein 206-like | Apoplast | 1.707 | 2.263E-04 |
| Csa3M828940.1 |  | Thaumatin-like protein-like | Extracellular region | 1.588 | 7.404E-05 |
| Csa6M404210.1 | HIR1 | Hypersensitive-induced response protein 1 | Plasma membrane | 1.789 | 2.077E-04 |
| Csa2M234530.1 | CXE1 | Probable carboxylesterase 1 (CXE1) -like |  | 1.837 | 1.595E-03 |
| Csa2M196900.1 | SEO1 | Sieve element occlusion protein 1-like |  | 1.744 | 7.655E-20 |
| Csa6M396650.1 |  | LysM domain-containing GPI-anchored protein 1-like | Plasma membrane | 0.291 | 1.322E-03 |
| Csa7M024010.1 |  | LysM domain-containing GPI-anchored protein 2-like | Plasma membrane | 0.332 | 1.438E-04 |
| **12. Transcription (16)** | | | | | |
| Csa5M177110.1 |  | Serine/arginine-rich splicing factor RS2Z32-like isoform 1 | Nucleus, Spliceosome | 0.632 | 8.467E-05 |
| Csa7M397040.2 |  | Pre-mRNA-splicing factor SF2-like | Nucleus, Spliceosome | 0.591 | 4.298E-04 |
| Csa6M150550.1 |  | Histone deacetylase HDT1-like | Nucleus | 0.621 | 0.03122 |
| Csa1M701970.1 |  | Mediator of RNA polymerase II transcription subunit 22b-like | Nucleus | 0.530 | 5.080E-05 |
| Csa3M168950.1 |  | U5 small nuclear ribonucleoprotein 200 kDa helicase-like | Nucleus | 0.448 | 5.507E-03 |
| Csa1M025950.1 |  | U2 small nuclear ribonucleoprotein B'' 2-like | Nucleus | 2.160 | 2.257E-08 |
| Csa5M266840.1 |  | U6 snRNA-associated Sm-like protein LSm6-like | Nucleus | 1.700 | 5.340E-06 |
| Csa4M001840.1 |  | U1 small nuclear ribonucleoprotein A-like | Nucleus | 3.088 | 3.602E-04 |
| Csa7M324150.1 |  | Transcription factor VIP1-like | Nucleus | 0.620 | 1.497E-03 |
| Csa6M426360.1 |  | BEL1-like homeodomain protein 1-like protein | Nucleus | 2.771 | 5.915E-08 |
| Csa4M269720.1 |  | Protein argonaute 4-like | Nucleus | 2.805 | 0.01648 |
| Csa1M408710.1 |  | Protein argonaute PNH1-like | Nucleus | 1.789 | 0.02602 |
| Csa2M093850.1 |  | DNA-directed RNA polymerase II subunit RPB3-A-like | Nucleus | 1.598 | 1.312E-15 |
| Csa6M008810.1 |  | GRF1-interacting factor 3-like | Nucleus, Cytoskeleton | 0.644 | 2.583E-04 |
| Csa1M039140.1 |  | Activating signal cointegrator 1 complex subunit 3-like | Nucleus | 1.587 | 1.171E-39 |
| Csa1M673530.1 |  | RNA polymerase beta subunit, chloroplastic | Chloroplast | 0.555 | 0.04742 |
| **13. Translation (32)** | | | | | |
| Csa2M006160.1 |  | NHP2-like protein 1-like | Cytosolic ribosome | 0.609 | 0.01524 |
| Csa3M889750.1 |  | DAZ-associated protein 1-like |  | 0.592 | 0.03636 |
| Csa2M060400.1 |  | 60S acidic ribosomal protein P3-2-like isoform 1 | Cytosolic ribosome | 0.474 | 5.918E-05 |
| Csa3M739040.1 |  | 60S ribosomal protein L12-1-like | Cytosolic ribosome | 1.517 | 6.553E-03 |
| Csa5M198130.1 |  | 60S ribosomal protein L35a-1-like | Cytosolic ribosome | 1.504 | 8.556E-05 |
| Csa4M292970.1 |  | 60S ribosomal protein L4-like | Cytosolic ribosome | 1.573 | 1.278E-04 |
| Csa7M432030.1 |  | 40S ribosomal protein S12-like | Cytosolic ribosome | 0.628 | 2.595E-07 |
| Csa1M042360.1 |  | 40S ribosomal protein S7-like | Cytosolic ribosome | 2.134 | 4.084E-08 |
| Csa7M428960.1 |  | 30S ribosomal protein 2, chloroplastic-like | Chloroplastic ribosome | 0.620 | 6.235E-03 |
| Csa3M769090.1 |  | 30S ribosomal protein S10, chloroplastic-like | Chloroplastic ribosome | 0.657 | 1.756E-03 |
| Csa4M630000.1 |  | 30S ribosomal protein S20, chloroplastic-like | Chloroplastic ribosome | 2.113 | 6.753E-14 |
| Csa3M837570.1 |  | 50S ribosomal protein L12, chloroplastic-like | Chloroplastic ribosome | 0.596 | 1.651E-04 |
| Csa7M274150.1 |  | 50S ribosomal protein L21, chloroplastic-like | Chloroplastic ribosome | 1.651 | 2.110E-03 |
| Csa3M121660.1 |  | 50S ribosomal protein L5, chloroplastic-like | Chloroplastic ribosome | 1.551 | 0.02040 |
| Csa2M337790.1 |  | Ribosome-binding factor PSRP1, chloroplastic-like | Chloroplastic ribosome | 2.720 | 5.459E-08 |
| Csa3M625080.1 |  | Ribonuclease P protein subunit p25-like protein-like | Nucleus | 2.017 | 1.958E-07 |
| Csa3M740070.1 |  | 29 kDa ribonucleoprotein, chloroplastic-like | Chloroplast | 0.592 | 4.930E-05 |
| Csa1M575050.1 |  | Arginyl-tRNA synthetase, cytoplasmic-like | Cytoplasm | 1.589 | 4.411E-05 |
| Csa3M185160.1 |  | Methionyl-tRNA formyltransferase-like | Cytoplasm | 1.612 | 1.739E-16 |
| Csa6M446550.1 |  | Histidine--tRNA ligase-like | Cytoplasm | 2.395 | 5.083E-13 |
| Csa7M033430.2 |  | Threonine--tRNA ligase-like | Cytoplasm | 0.502 | 0.01701 |
| Csa2M377990.1 |  | Putative peptidyl-tRNA hydrolase PTRHD1-like | Cytoplasm | 0.622 | 0.02738 |
| Csa7M394660.1 |  | Peptide chain release factor 1-like | Cytoplasm | 2.068 | 5.163E-06 |
| Csa2M079620.1 |  | Translational activator GCN1-like | Cytosolic ribosome | 1.909 | 2.862E-04 |
| Csa2M310390.1 |  | Eukaryotic translation initiation factor 5-like | Cytoplasm | 3.325 | 2.439E-03 |
| Csa2M263980.1 |  | Translation initiation factor-like | Cytoplasm | 0.561 | 6.305E-05 |
| Csa2M036070.1 |  | Translation initiation factor IF-3-like | Cytoplasm | 1.569 | 6.040E-04 |
| Csa7M209580.1 |  | Protein translation factor SUI1 homolog 1-like | Cytoplasm | 0.663 | 9.498E-12 |
| Csa3M728090.1 |  | Elongation factor P-like | Cytoplasm | 2.930 | 6.757E-03 |
| Csa3M588450.1 |  | Chain U For The Spinach Chloroplast 30S Subunit | Chloroplastic ribosome | 0.652 | 9.166E-03 |
| Csa1M063550.1 |  | Polyadenylate-binding protein RBP45-like | Cytosol | 0.613 | 3.114E-03 |
| Csa3M134680.1 |  | U-box domain-containing protein 72-like | Nucleus | 2.202 | 1.833E-03 |
| **14. Nucleotide metabolism (5)** | | | | | |
| Csa5M613530.1 | NUDT26 | Nudix hydrolase 26, chloroplastic-like | Chloroplast | 0.616 | 2.709E-17 |
| Csa2M354800.2 | APRT1 | Adenine phosphoribosyltransferase 1 (APRT1), chloroplastic-like | Chloroplast | 0.515 | 4.277E-04 |
| Csa4M297440.2 | UPRT | Uracil phosphoribosyltransferase-like, chloroplastic precursor | Chloroplast | 0.658 | 0.01407 |
| Csa3M002820.1 |  | FHIT, bis(5'-adenosyl)-triphosphatase-like | Chloroplast | 2.926 | 0.02343 |
| Csa2M008770.1 | AMK | Adenylate kinase family protein-like | Chloroplast | 2.324 | 1.375E-03 |
| **15. Amino acid metabolism (14)** | | | | | |
| Csa4M563190.2 | ASI-2 | Anthranilate synthase component I-2, chloroplastic-like | Chloroplast | 0.308 | 3.637E-05 |
| Csa3M891650.1 | ASL | Argininosuccinate lyase-like | Chloroplast | 1.544 | 0 |
| Csa5M153150.1 | GCS | Glutamate--cysteine ligase, Gamma-glutamylcysteine synthetase (GCS), chloroplastic-like | Chloroplast | 2.081 | 6.464E-19 |
| Csa3M824870.1 | CGS | Cystathionine gamma-synthase, chloroplastic-like | Chloroplast | 1.512 | 0.01224 |
| Csa7M064020.1 |  | Phospho-2-dehydro-3-deoxyheptonate aldolase 2, chloroplastic-like | Chloroplast | 1.503 | 0.01407 |
| Csa1M599580.1 | MS | 5-methyltetrahydropteroyltriglutamate--homocysteine methyltransferase-like isoform 1 | Chloroplast | 1.757 | 1.355E-07 |
| Csa3M816040.1 | CS | Probable cysteine synthase, chloroplastic-like | Chloroplast | 2.352 | 1.132E-05 |
| Csa7M209030.1 |  | Dihydrodipicolinate reductase 2, chloroplastic-like | Chloroplast | 0.654 | 5.079E-09 |
| Csa5M177080.1 |  | Nitrilase homolog 1-like | Chloroplast | 0.529 | 6.856E-09 |
| Csa6M291910.1 |  | Isovaleryl-CoA dehydrogenase, mitochondrial-like | Mitochondiron | 0.652 | 0.00867 |
| Csa6M004600.1 | CS2 | L-3-cyanoalanine synthase 2, Cysteine synthase 2, mitochondrial-like isoform 1 | Mitochondiron | 1.635 | 1.271E-04 |
| Csa7M073600.1 | MCC-β | Methylcrotonoyl-CoA carboxylase (MCC) beta chain, mitochondrial-like | Mitochondiron | 0.537 | 3.288E-06 |
| Csa2M145880.1 | SHMT | Serine hydroxymethyltransferase 1-like | Mitochondiron | 1.594 | 3.665E-08 |
| Csa1M701910.1 | GLDP | Glycine cleavage system H protein, mitochondrial-like | Mitochondiron | 0.611 | 0.00765 |
| **16. Structural molecules (5)** | | | | | |
| Csa3M159460.1 |  | Dynein light chain 1, cytoplasmic-like | Cytoskeleton | 0.607 | 4.213E-04 |
| Csa7M056470.1 |  | Actin-like | Cytoskeleton | 1.520 | 2.069E-12 |
| Csa3M842710.1 |  | Tubulin alpha-3 chain-like | Cytoskeleton | 1.525 | 1.747E-15 |
| Csa5M166470.1 |  | Kinesin-like protein KIF22-like | Cytoskeleton | 1.589 | 2.186E-04 |
| Csa4M026900.1 |  | Histone H3.3-like | Nucleus | 3.868 | 2.623E-11 |
| **17. Cell cycle (3)** | | | | | |
| Csa1M172630.1 |  | Sister chromatid cohesion protein PDS5 homolog B-like | Nucleus | 2.568 | 5.054E-09 |
| Csa3M740140.1 |  | Cell division cycle protein 48 homolog | Nucleus | 0.667 | 1.007E-03 |
| Csa6M188070.1 |  | Proliferating cell nuclear antigen-like | Nucleus | 1.557 | 2.421E-24 |
| **18. Unclassified proteins (19)** | | | | | |
| Csa3M776950.1 |  | Heme-binding-like protein (similar to At3g10130), chloroplastic-like | Chloroplast | 4.364 | 4.012E-03 |
| Csa5M148600.1 |  | Metal-dependent protein hydrolase-like | Mitochondrion | 0.627 | 0.01268 |
| Csa1M228960.1 |  | Extensin-like protein | Cell wall | 0.553 | 3.552E-05 |
| Csa2M372150.1 |  | 14-3-3 protein 10-like |  | 1.632 | 4.123E-15 |
| Csa5M635360.1 |  | Inositol hexakisphosphate and diphosphoinositol-pentakisphosphate kinase 1-like |  | 0.530 | 1.080E-03 |
| Csa5M162620.1 |  | Inositol 2-dehydrogenase like protein |  | 2.163 | 1.553E-05 |
| Csa1M039010.1 |  | LYR motif-containing protein 4-like |  | 0.651 | 5.840E-04 |
| Csa6M538630.1 |  | MOSC domain-containing protein 2, mitochondrial-like | Mitochondrion | 0.586 | 0.03842 |
| Csa3M912300.1 |  | Pentatricopeptide repeat-containing protein At4g17616-like | Mitochondrion | 1.605 | 4.986E-14 |
| Csa2M381890.1 |  | 14 kDa proline-rich protein DC2.15-like |  | 0.447 | 2.231E-15 |
| Csa3M021150.1 |  | 14 kDa proline-rich protein DC2.15-like |  | 0.540 | 6.106E-04 |
| Csa4M641650.1 |  | Protein DCL (defective chloroplasts and leaves), chloroplastic-like | Chloroplast | 0.463 | 4.000E-07 |
| Csa4M663680.1 |  | High mobility group 1/2 (HMG1/2)-like protein | Nucleus | 0.322 | 7.715E-06 |
| Csa3M563290.1 |  | Major pollen allergen-like |  | 1.747 | 0.03130 |
| Csa2M174120.1 |  | Probable methyltransferase PMT8-like |  | 1.600 | 6.615E-09 |
| Csa2M108610.1 |  | Probable methyltransferase PMT2-like |  | 1.847 | 0.01078 |
| Csa3M636940.1 |  | Probable methyltransferase PMT21-like |  | 2.213 | 1.168E-05 |
| Csa6M510920.1 |  | 4-nitrophenylphosphatase-like |  | 4.709 | 6.747E-04 |
| Csa1M152500.1 |  | CBS (Cystathionine beta-synthase) domain-containing protein CBSX1, chloroplastic-like | Chloroplast | 1.646 | 2.709E-37 |
| **19. Uncharacterized proteins (40)** | | | | | |
| Csa6M046350.1 |  | Uncharacterized protein At2g39795, mitochondrial-like | Mitochondrion | 2.903 | 2.705E-45 |
| Csa3M159420.1 |  | Uncharacterized protein LOC101205403 |  | 0.656 | 0.01984 |
| Csa1M665900.1 |  | Uncharacterized protein LOC101217511 |  | 3.570 | 3.933E-04 |
| Csa5M638300.1 |  | Uncharacterized protein LOC101202842 |  | 1.540 | 0.03342 |
| Csa2M297210.2 |  | Uncharacterized mitochondrial carrier C12B10.09-like | Mitochondrion | 1.501 | 0.03129 |
| Csa4M110050.1 |  | Uncharacterized protein LOC101213735 |  | 2.905 | 3.305E-03 |
| Csa3M047750.1 |  | Hypothetical protein |  | 1.573 | 6.758E-08 |
| Csa2M072460.1 |  | Uncharacterized protein LOC101212517 |  | 1.819 | 4.319E-04 |
| Csa6M486890.1 |  | Uncharacterized protein LOC101206379 |  | 4.713 | 9.509E-07 |
| Csa7M013950.1 |  | Uncharacterized protein LOC101228360 |  | 3.201 | 6.164E-11 |
| Csa5M505130.1 |  | Uncharacterized protein LOC101221723 |  | 0.664 | 1.281E-03 |
| Csa6M511710.1 |  | Uncharacterized protein LOC101206738 isoform 1 |  | 0.558 | 0.01277 |
| Csa3M824890.1 |  | Uncharacterized protein At5g39570-like |  | 0.501 | 9.724E-07 |
| Csa3M119290.1 |  | Uncharacterized protein LOC101205715 |  | 0.567 | 0.02153 |
| Csa2M049880.1 |  | Uncharacterized protein LOC101215475 |  | 0.567 | 2.053E-05 |
| Csa5M652290.1 |  | Uncharacterized protein LOC101228524 |  | 0.583 | 2.194E-06 |
| Csa3M186700.1 |  | Uncharacterized protein LOC101206373 isoform 1 |  | 0.595 | 4.177E-08 |
| Csa6M522110.1 |  | Uncharacterized protein LOC101211388 |  | 0.607 | 1.216E-05 |
| Csa6M187910.1 |  | Uncharacterized protein LOC101205268 |  | 0.610 | 4.717E-11 |
| Csa6M500430.1 |  | Uncharacterized protein LOC101209377 |  | 0.616 | 1.112E-04 |
| Csa4M615230.1 |  | Uncharacterized oxidoreductase At1g06690, chloroplastic-like | Chloroplast | 0.616 | 5.073E-04 |
| Csa4M664320.1 |  | Uncharacterized protein LOC101206784 |  | 0.641 | 0.02156 |
| Csa5M139490.1 |  | Uncharacterized protein LOC101227735 isoform 1 |  | 0.649 | 7.782E-11 |
| Csa3M426340.1 |  | Uncharacterized protein LOC101212269 |  | 0.650 | 1.657E-07 |
| Csa5M606550.1 |  | Uncharacterized protein LOC101213972 |  | 0.652 | 2.437E-06 |
| Csa3M027190.1 |  | Uncharacterized protein LOC101209814 |  | 0.663 | 1.217E-05 |
| Csa7M446650.1 |  | Uncharacterized protein LOC101224991 |  | 0.634 | 2.980E-05 |
| Csa4M173560.1 |  | Uncharacterized protein LOC101209235 |  | 1.540 | 1.204E-22 |
| Csa6M045110.1 |  | Unknown protein DS12 from 2D-PAGE of leaf, chloroplastic-like | Chloroplast | 1.554 | 4.680E-07 |
| Csa6M082530.1 |  | Uncharacterized protein LOC101207511 |  | 1.583 | 5.945E-05 |
| Csa5M588750.1 |  | Uncharacterized protein LOC101217329 |  | 1.772 | 0.03153 |
| Csa3M888540.1 |  | Uncharacterized protein LOC101216465 |  | 1.784 | 8.339E-04 |
| Csa3M585350.2 |  | Uncharacterized protein HI_1198-like |  | 1.800 | 5.297E-04 |
| Csa5M155600.1 |  | Uncharacterized protein LOC101205107 |  | 2.175 | 4.276E-03 |
| Csa2M020900.1 |  | Uncharacterized protein LOC101214471 isoform 2 |  | 2.278 | 3.519E-07 |
| Csa4M197820.1 |  | Uncharacterized protein LOC101218161 |  | 2.630 | 4.019E-05 |
| Csa3M132000.1 |  | Uncharacterized protein LOC101215886 |  | 2.976 | 0.02249 |
| Csa1M476000.1 |  | Uncharacterized protein LOC101204733 |  | 3.108 | 6.566E-04 |
| Csa5M464810.1 |  | Uncharacterized protein LOC101223187 |  | 3.763 | 5.751E-04 |
| Csa7M026250.1 |  | Uncharacterized protein LOC101220371 |  | 2.786 | 2.495E-03 |
