# Supplementary material for: Quantitative Proteomic Profiling of Early and Late Responses to Salicylic Acid in Cucumber Leaves
Source: PLoS One. 2016 Aug 23;11(8):e0161395. doi: 10.1371/journal.pone.0161395 (PMC4995040; doi:10.1371/journal.pone.0161395)
Supplement: S1 Text — (DOCX) [file pone.0161395.s012.docx]

**Supporting Information**

**Transporters**

A subset of DEPs that are involved in cellular transport system was identified, including the transporters responsible for ions, water and proteins. At 12 hpt, two putative plasma-membrane-type ATPases (ATPase 8 and 9) were down-regulated. In plant cells, these H^+^-ATPases could generate an H^+^-gradient and result in external acidification. In cucumber seedlings, when SA was applied to the roots, exogenous SA could be transported to the leaves (Figure 1). In leaves, the down-regulated expression of plasma-membrane-type ATPases might generate an external alkalized environment and facilitate the unloading of SA. Another transporter that was down-regulated at 12 hpt was the probable chloroplasticsodium/pyruvate cotransporter BASS2. BASS2 was required for Na^+^-coupled pyruvate import into chloroplasts [1]. Pyruvate is a metabolic precursor for many plastid-localized biosynthetic pathways, such as those for fatty acids, terpenoids and branched-chainamino acids. In spite of the importance of pyruvate uptake into plastids, the resulting high Na^+^ levels in chloroplasts impaired the photosynthetic performance [2]. In cucumber leaves, the decreased level of BASS2 by SA during the early responses would ensure the elevated photosynthesis, after which the repression was released, and pyruvate was taken into the chloroplast as a biosynthetic precursor. At 72 hpt, two probable aquaporin PIP1s were induced by SA treatment. Aquaporin can facilitate the transport of water and small neutral solutes (urea, boric acid, and silicic acid) and gases (NH_3_ and CO_2_) [3]. The promoted uptake of these solutes would provide material for enhanced plant growth after SA application. Furthermore, a probable chloroplastic Cu^2+^-binding protein CutA and a mitochondrial phosphate carrier protein were up-regulated by SA treatment. The imported Cu^2+^ and phosphate could be used as coenzymes for photosynthetic enzymes or ATP production by oxidative phosphorylation [4].

In addition, a series of transporters that are responsible for exporting/importing proteins from/to their target subcellular organelles were regulated after SA treatment, suggesting that SA might regulate protein localization at the post-translational level, in addition to the protein modifications described below.

**Proteolysis, protein folding and modification**

Upon SA application, ten and twelve DEPs that are involved in proteolysis were identified at 12 hpt and 72 hpt, respectively. Most of these DEPs were up-regulated, including the subunits of cytosol T-complex protein 1 and 26S proteasome, the mitochondrial protease 2, and the subunits of ATP-dependent Clp protease and zinc metalloprotease FTSH localized in chloroplasts, as well as the subtilisin-like protease in the cell wall. However, a putative chloroplastic metacaspase 5 (MCP5) was down-regulated. In Arabidopsis, MCP5 is a cysteine protease that is involved in the modulation of ROS-activated programmed cell death [5]. The down-regulation of MCP5 by SA indicated that at low concentrations, SA might not induce cell death, although an ROS burst following SA treatment.

In the context of protein folding, nine DEPs were identified after SA treatment. Three probable prefoldin subunits were up-regulated during the early phase and down-regulated during the late phase. Prefoldinbinds to the cytosolic chaperonin and transfers the nascent polypeptide chains to it, thereby allowing these proteins to be folded correctly [6]. Additionally, two probable chloroplastic chaperonin subunits and one mitochondrial prohibitin-1 were also down-regulated by SA at 72 hpt. The changes in these proteins indicate that chaperonins might be involved in protecting the nascent proteins in multiple subcellular organelles during the early SA-responsive phase.

Two and seven DEPs that are involved in protein post-transcriptional modification were also identified at 12 hpt and 72 hpt, respectively. Most of these proteins are kinases or phosphatases, indicating the potential roles of protein phosphorylation and dephosphorylation in SA-mediated signalling. A putative dolichyl-diphosphooligosaccharide-protein glycosyltransferase subunit 2, which could catalyze the transfer of high-mannose oligosaccharides from a lipid-linked oligosaccharide donor to an asparagine residue, was up-regulated at 72 hpt. The protein *N*-glycosylation that is mediated by this complex is involved in the adaptive responses to salt/osmatic stress and in plant innate immunity responses [7, 8]. Additionally, upon SA treatment, the SUMO-conjugating enzyme SCE1-like protein increased slightly at 12 hpt (1.38-fold) and decreased at 72 hpt. During viral infection, SCE1 can interact with the geminivirus replication protein or the RNA-dependent RNA polymerase from the virus, facilitating virus infection [9, 10]. The negative relationship between sumoylation and SA signalling was also substantiated by studies of the SUMO E3 ligase SIZ1. In a previous report, the SIZ1-mediated sumoylation of histone or chromatin modifiers might have played a role in the transcriptional repression of SA-responsive genes in plant cells [11].

**Cell wall organization**

Upon SA treatment, a series of proteins that are involved in cell wall organization were differentially expressed. For example, a putative endo-1,3;1,4-β-D-glucanase was down-regulated at 12 hpt, and two proteins, expansin-like A1 (EXLA1) and polygalacturonase At1g48100-like protein (PG), were down-regulated at 72 hpt. 1,3;1,4-β-Glucan interacts with xylan and xyloglucan, and its composites with hemicellulosic polysaccharides form inter-molecular bridges. The degradation of these bridges by 1,3;1,4-β-glucanase decreased the physical properties, resulting in an increased extensibility of the cell walls [12]. EXLA1 is expressed in very young vascular bundles and might cause the loosening and extension of cell walls by disrupting the non-covalent bonding between cellulose microfibrils and matrix glucans [13]. Moreover, a putative β-D-xylosidase 1 (BXL1) was down-regulated by SA at 72 hpt. In Arabidopsis, BXL1 was responsible for xylan degradation by cleaving the xylan backbone. Xylan is the major component of hemicelluloses and can form cross-linkages with lignin in cell walls [14, 15]. The down-regulation of BXL1 by SA in cucumber seedlings indicates the potentially negative roles of SA in xylan remodelling.

Furthermore, four DEPs were up-regulated upon SA treatment, of which one DEP was up-regulated at 12 hpt, and the other three at 72 hpt. At 12 hpt, the level of the glucan endo-1,3-β-glucosidase-like protein At1g69295-like protein was 1.57-fold over the control. In Arabidopsis, At1g69295 is also as plasmodesmatacallose-binding protein 4 (PDCB4). The increased expression of PDCBs leads to an increase in callose accumulation in cell walls surrounding the plasmodesmata and provides a physical constriction for the symplastic channel. The reduced cell-to-cell trafficking would decrease the susceptibility to virus invasion [16]. Coincidentally, a putative callose synthase 9 (CALS9) was up-regulated after SA treatment. In addition, at 72 hpt, an α-1,4-glucan-protein synthase [UDP-forming]-like protein (UPTG) and a putative β-glucosidase 24 (βGLU24) localized at the cell wall were up-regulated at 72 hpt with SA. These proteins are involved in lignification and cell wall reorganization by the hydrolysis of cell-wall-derived polysaccharides [17, 18]. These results suggest that SA might induce cell wall reorganization and thickeningand thereby enhance plant growth and stress resistance.

**Stress responses**

Another group of DEPs included stress-responsive proteins. At 12 hpt, six DEPs were identified, of which three were down-regulated, and the other three were up-regulated. At 72 hpt, 14 DEPs were identified, nine of which were up-regulated. A putative aldo-keto reductase family 4 member C9 (AKR4C9) was repressed (0.61-fold) at 12 hpt, while another Tas-like AKR protein was up-regulated (1.51-fold) at 72 hpt. AKR can function as a detoxifying enzyme by reducing a range of toxic aldehydes and ketones that are produced during various stresses [19, 20]. For the universal stress proteins (USPs), albeit a transient repression at 12 hpt, their expression increased at 72 hpt. In addition todirect induction by SA, the SA-induced uncoupling of oxidative phosphorylation might be an internal stimulus for the enhanced expression of USPs [21]. After SA treatment, the levels of hypersensitive-induced response protein 1 (HIR1) and probable carboxylesterase 1 (CXE1) constitutively increased. Consistent with this, HIR1 is involved in the rapid and localized hypersensitive responses against the pathogen-induced SA accumulation in rice [22]. CXE1 functions as a detoxification enzyme; its esterase activity can detoxify the phytotoxic esters that are produced by pathogens and release the antimicrobial isoflavones from their inactive glycosidic conjugates [23]. Additionally, two LysM domain-containing GPI-anchored proteins (LYM1 and LYM2) were down-regulated by SA treatment. LYM proteins constitute a vascular plant-specific family and are positioned to the outer face of the plasma membranes by their GPI-anchors. During plant immunity responses, LYMs are required as perception sites for peptidoglycan and chitin elicitors and restrict growth of infected pathogens [24, 25]. However, without pathogen attack, the levels of LYM proteins decreased upon SA treatment, indicating the different fates of immunity proteins upon pathogen infection and treatment with SA alone. Another interesting SA-responsive protein is pyrroline-5-carboxylate reductase (P5CR), which was dramatically up-regulated (2.26-fold) at 72 hpt. P5CR is involved in proline biosynthesis, although it is not the rate-limiting factor. Proline plays important roles in cellular osmotic regulation, redox buffering and energy transfer [26]. SA-induced P5CR is consistent with our previous finding that SA pretreatment can induce the expression of the *P5CS* gene under chilling conditions in the leaves of cucumber seedlings [27] and indicates that SA might enhance proline biosynthesis in cucumber seedlings.

**Nucleotide and amino acid metabolism**

In the context of nucleotide metabolism, four SA-responsive DEPs were identified at 12 hpt, of which three proteins were down-regulated (Additional file 2). These down-regulated DEPs were guanine deaminase-like protein, chloroplastic phosphoribosylamine-glycine ligase, and chloroplastic Nudix hydrolase 20. All of these proteins are predicted to be involved in the catabolism of nucleosides [28-30]. The only one up-regulated protein during the early phage of SA treatment was UMP/CMP kinase 1, which plays an important role in *de novo* pyrimidine nucleotide biosynthesis [31].

At 72 hpt, five DEPs were identified, of which three proteins were down-regulated, and the other two were up-regulated (Additional file 2). Two down-regulated proteins, chloroplastic adenine phosphoribosyltransferase 1 (APRT1) and uracil phosphoribosyltransferase protein (UPRT), catalyze the salvage reaction, resulting in the formation of AMP and UMP from adenine and uracil, respectively. The salvage pathway is an energy-saving pathway that utilizes the pre-formed nucleosides from the catabolism of nucleotides [32, 33]. Additionally, in Arabidopsis, APRT1 catalyzesthe conversion of cytokinins (CKs) from free bases (active form) to the corresponding nucleotides (inactive form), implying a potential APRT1-mediated synergistic interaction between SA and CKs. A similar synergistic interaction was also reported previously [34]. One of the up-regulated DEPs was a bis(5'-adenosyl)-triphosphatase-like protein that can cleave bis(5'-adenosyl) triphosphate to yield AMP and ADP. Another up-regulated DEP was a putative chloroplastic adenylate kinase family protein (AMK). AMK can catalyze the reversible formation of ADP by transferring one phosphate group from ATP to AMP [35]. The changes in these proteins indicate that SA might play an important role in adenylate equilibration and cellular energy homeostasis.

After SA treatment, four and fourteen DEPs that are involved in amino acid metabolism were identified at 12 hpt and 72 hpt, respectively. The chloroplastic anthranilate synthase component I-2 (ASI-2) was up-regulated (2.45-fold) at 12 hpt and down-regulated (0.31-fold) at 72 hpt. ASI-2 can catalyze the biosynthesis of anthranilate, an intermediate in tryptophan biosynthesis [36, 37]. Tryptophan serves as the precursor for auxin synthesis, indicating a potential crosslink between SA and auxin. Regarding glutathione metabolism, a putative lactoylglutathione lyase (glyoxalase I, GLX1) that is involved in glutathione degradation [38] was repressed at 12 hpt. γ-glutamylcysteine synthetase (GCS), a putative protein that is involved in glutathione biosynthesis [39], was induced by 2.08-fold at 72 hpt. These results suggest a decreasing trend after the first increases after SA treatment. The changing patterns of glutathione-metabolic proteins are consistent with the SA-induced changing profiles of H_2_O_2_ (Figure 3). In total, at 72 hpt, proteins that are involved in amino acids biosynthesis in chloroplast increased, while proteins that are involved in amino acids degradation in mitochondria decreased, suggesting the positive effects of SA on the amino acid contents during the late-responsive phase.

**Unclassified and uncharacterized proteins**

The group of unclassified proteins contained 19 DEPs whose functional prediction was not included in the functional classification mentioned in this study. And the uncharacterized proteins contained the DEPs who have no blast hit with functional description to be matched. The unclassified and uncharacterized proteins would be an interesting group because it would be used as protein biomarker for the early and late SA responsiveness. Indeed some DEPs displayed strong time-dependent expression changes. For example, the abundance of proteins “Csa6M046350.1” and “Csa1M665900.1” underwent substantial reduction at 12 hpt but dramatic induction at 72 hpt, although more efforts would be made to confirm the level changes.

**References:**

1. Furumoto T, Yamaguchi T, Ohshima-Ichie Y, Nakamura M, Tsuchida-Iwata Y, Shimamura M, et al. A plastidial sodium-dependent pyruvate transporter. Nature 2011; 476: 472-475.
2. Müller M, Kunz HH, Schroeder JI, Kemp G, Young HS, Neuhaus HE. Decreased capacity for sodium export out of Arabidopsis chloroplasts impairs salt tolerance, photosynthesis and plant performance. Plant J 2014; 78: 646-658.
3. Maurel C, Verdoucq L, Luu DT, Santoni V. Plant aquaporins: membrane channels with multiple integrated functions. *Ann Rev Plant Biol* 2008, 59:595-624.
4. Rausch C, Bucher M. Molecular mechanisms of phosphate transport in plants. Planta 2002; 216: 23-37.
5. Watanabe N, Lam E. Two Arabidopsis metacaspases AtMCP1b and AtMCP2b are arginine/lysine-specific cysteine proteases and activate apoptosis-like cell death in yeast. J Biol Chem 2005; 280: 14691-14699.
6. Rodríguez-Milla MA, Salinas J. Prefoldins 3 and 5 play an essential role in Arabidopsis tolerance to salt stress. Mol Plant 2009; 2: 526-534.
7. Koiwa H, Li F, McCully MG, Mendoza I, Koizumi N, Manabe Y, **et al.** The STT3a subunit isoform of the Arabidopsis oligosaccharyltransferase controls adaptive responses to salt/osmotic stress. Plant Cell 2003**;** 15: 2273-2284.
8. Nekrasov V, Li J, Batoux M, Roux M, Chu ZH, Lacombe S, et al. Control of the pattern-recognition receptor EFR by an ER protein complex in plant immunity. EMBO J 2009; 28: 3428-3438.
9. Sánchez-Durán MA, Dallas MB, Ascencio-Ibañez JT, Reyes MI, Arroyo-Mateos M, Ruiz-Albert J, et al. Interaction between geminivirus replication protein and the SUMO-conjugating enzyme is required for viral infection. J Virol 2011; 85: 9789-9800.
10. Xiong R, Wang A. SCE1, the SUMO-conjugating enzyme in plants that interacts with NIb, the RNA-dependent RNA polymerase of Turnip mosaic virus, is required for viral infection. J Virol 2013; 87: 4704-4715.
11. van den Burg HA, Kini RK, Schuurink RC, Takken FLW. Arabidopsis small ubiquitin-like modifier paralogs have distinct functions in development and defense. Plant Cell 2010; 22: 1998-2016.
12. Takahashi M, Yamamoto R, Sakurai N, Nakano Y, Takeda T. Fungal hemicellulose-degrading enzymes cause physical property changes concomitant with solubilization of cell wall polysaccharides. Planta 2015; 241: 359-370.
13. Cosgrove DJ. Plant expansins: diversity and interactions with plant cell walls. Curr Opin Plant Biol 2015; 25: 162-172.
14. Goujon T, Minic Z, El Amrani A, Lerouxel O, Aletti E, Lapierre C, et al. AtBXL1, a novel higher plant (*Arabidopsis thaliana*) putative beta-xylosidase gene, is involved in secondary cell wall metabolism and plant development. Plant J 2003; 33: 677-690.
15. Minic Z, Rihouey C, Do CT, Lerouge P, Jouanin L. Purification and characterization of enzymes exhibiting beta-D-xylosidase activities in stem tissues of Arabidopsis. Plant Physiol 2004; 135: 867-878.
16. Simpson C, Thomas C, Findlay K, Bayer E, Maule AJ. An Arabidopsis GPI-anchor plasmodesmal neck protein with callose binding activity and potential to regulate cell-to-cell trafficking. Plant Cell 2009; 21: 581-594.
17. Opassiri R, Pomthong B, Onkoksoong T, Akiyama T, Esen A, Ketudat Cairns JR. Analysis of rice glycosyl hydrolase family 1 and expression of Os4bglu12 beta-glucosidase. BMC Plant Biol 2006; 6: 33.
18. Bocca SN, Kissen R, Rojas-Beltrán JA, Noël F, Gebhardt C, Moreno S, et al. Molecular cloning and characterization of the enzyme UDP-glucose: protein transglucosylase from potato. Plant Physiol Biochem 1999; 37: 809-819.
19. Simpson PJ, Tantitadapitak C, Reed AM, Mather OC, Bunce CM, White SA, et al. Characterization of two novel aldo-keto reductases from Arabidopsis: expression patterns, broad substrate specificity, and an open active-site structure suggest a role in toxicant metabolism following stress. J Mol Biol 2009; 392: 465-480.
20. Obmolova G, Teplyakov A, Khil PP, Howard AJ, Camerini-Otero RD, Gilliland GL. Crystal structure of the *Escherichia coli* Tas protein, an NADP(H)-dependent aldo-keto reductase. Proteins 2003; 53: 323-325.
21. Kim DJ, Bitto E, Bingman C, Kim HJ. Crystal structure of the protein At3g01520, a eukaryotic universal stress protein-like protein from *Arabidopsis thaliana* in complex with AMP. Proteins 2015; 83: 1368-1373.
22. Zhou L, Cheung MY, Li MW, Fu Y, Sun Z, Sun SM, et al. Rice hypersensitive induced reaction protein 1 (OsHIR1) associates with plasma membrane and triggers hypersensitive cell death. BMC Plant Biol 2010; 10: 290.
23. Gershater MC, Edwards R. Regulating biological activity in plants with carboxylesterases. Plant Sci 2007; 173: 579-588.
24. Willmann R, Lajunen HM, Erbs G, Newman MA, Kolb D, Tsuda K, et al. Arabidopsis lysin-motif proteins LYM1 LYM3 CERK1 mediate bacterial peptidoglycan sensing and immunity to bacterial infection. Proc Natl Acad Sci USA 2011; 108: 19824-19829.
25. Shinya T, Motoyama N, Ikeda A, Wada M, Kamiya K, Hayafune M, et al. Functional characterization of CEBiP and CERK1 homologs in Arabidopsis and rice reveals the presence of different chitin receptor systems in plants. Plant Cell Physiol 2012; 53: 1696-1706.
26. Verslues PE, Sharma S. Proline metabolism and its implications for plant-environment interaction. Arabidopsis Book 2010; 8: e0140.
27. Dong CJ, Li L, Shang QM, Liu XY, Zhang ZG. Endogenous salicylic acid accumulation is required for chilling tolerance in cucumber (*Cucumis sativus* L.) seedlings. Planta 2014; 240: 687-700.
28. Dahncke K, Witte CP. Plant purine nucleoside catabolism employs a guanosine deaminase required for the generation of xanthosine in Arabidopsis. Plant Cell 2013; 25: 4101-4109.
29. Stasolla C, Katahira R, Thorpe TA, Ashihara H. Purine and pyrimidine nucleotide metabolism in higher plants. J Plant Physiol 2003; 160: 1271-1295.
30. Ogawa T, Yoshimura K, Miyake H, Ishikawa K, Ito D, Tanabe N, et al. Molecular characterization of organelle-type Nudix hydrolases in Arabidopsis. Plant Physiol 2008; 148: 1412-1424.
31. Zhou L, Lacroute F, Thornburg R. Cloning, expression in *Escherichia coli*, and characterization of *Arabidopsis thaliana* UMP/CMP kinase. Plant Physiol 1998; 117: 245-254.
32. Zhang X, Chen Y, Lin X, Hong X, Zhu Y, Li W, et al. Adenine phosphoribosyl transferase 1 is a key enzyme catalyzing cytokinin conversion from nucleobases to nucleotides in Arabidopsis. Mol Plant 2013; 6: 1661-1672.
33. Mainguet SE, Gakière B, Majira A, Pelletier S, Bringel F, Guérard F, et al. Uracil salvage is necessary for early Arabidopsis development. Plant J 2009; 60: 280-291.
34. Naseem M, Kaltdorf M, Dandekar T: The nexus between growth and defence signaling: auxin and cytokinin modulate plant immune response pathways. J Exp Bot 2015; 66: 4885-4896.
35. Lange PR, [Geserick C](http://www.ncbi.nlm.nih.gov/pubmed/?term=Geserick%20C%5BAuthor%5D&cauthor=true&cauthor_uid=18162585), Tischendorf G, Zrenner R. Functions of chloroplastic adenylate kinases in Arabidopsis. Plant Physiol 2008; 146: 492-504.
36. Tozawa Y, Hasegawa H, Terakawa T, Wakasa K. Characterization of rice anthranilate synthase alpha-subunit genes *OASA1* and *OASA2*. Tryptophan accumulation in transgenic rice expressing a feedback-insensitive mutant of OASA1. Plant Physiol 2001; 126: 1493-1506.
37. Ishihara A, Asada Y, Takahashi Y, Yabe N, Komeda Y, Nishioka T, et al. Metabolic changes in *Arabidopsis thaliana* expressing the feedback-resistant anthranilate synthase alpha subunit gene *OASA1D*. Phytochem 2006; 67: 2349-2362.
38. Usui Y, Nakase M, Hotta H, Urisu A, Kitajima K, Matsuda T. A 33-kDa allergen from rice (*Oryza sativa* L. Japonica). cDNA cloning, expression, and identification as a novel glyoxalase I. J Biol Chem 2001; 276: 11376-11381.
39. Parisy V, Poinssot B, Owsianowski L, Buchala A, Glazebrook J, Mauch F. Identification of PAD2 as a gamma-glutamylcysteine synthetase highlights the importance of glutathione in disease resistance of Arabidopsis. Plant J 2007; 49: 159-172.
